# Identifying the effect of retail brands on private residential rental prices in Great Britain

**DOI:** 10.1007/s10901-021-09904-2

**Published:** 2021-10-05

**Authors:** Stephen Clark, Nick Hood, Mark Birkin

**Affiliations:** 1 School of Geography, University of Leeds, LEEDS, LS2 9JT UK; 2 Leeds Institute for Data Analytics and School of Geography, University of Leeds, LEEDS, LS2 9JT UK

**Keywords:** Retail, Housing, Private Rental, Great Britain, Propensity Score Matching

## Abstract

This study extends our understanding of the influence of proximity to retail grocery provision on housing rental prices. To achieve this, extensive data on the size and location of retail outlets are combined with neighbourhood rental values for small areas across a two year period, together with varied contextual data for each area. In order to control the influence of many confounding variables in the determination of housing rentals, the technique of propensity score matching is applied. This provides a sophisticated means for the comparison between areas where there is substantial natural variation, rather than manageable controls. For a variety of types of retail brands, only a significant relationship is found between the proximity of a Premium retail outlet and the housing rental value. The findings of this research allow local planning officers to further understand the impact of planning applications on the potential for gentrification and the affordability of neighbouring housing.

**Supplementary Information:**

The online version contains supplementary material available at 10.1007/s10901-021-09904-2.

## Introduction

The retail sector is an important component of the built environment in many towns and cities, creating jobs, economic activity and leisure opportunities (Wrigley et al., 2002), but also bringing with it issues around traffic, air pollution and crime (Black et al., 2007).

One aspect of the built environment that has a close association with retail is housing. Generally, there is an extensive literature that attempts to understand what features influence the housing market. In particular economists and policy makers have an interest in what characteristics help to determine the ‘price’ of housing, represented as either a sales price or a rental price. It is known that these prices can be influenced by many factors: on a macro-scale, such as interest rates or land availability; on a micro-scale, such as quality of local provision or access to services; and also by individual properties, for example the size and up-keep of the property. In this study we focus on the influence of differing brands of local grocery retail provision on the private residential rental market in Great Britain (GB).

Grocery provision is not uniform in GB. Retailers employ various strategies to expand their provision in an effort to increase their share of the market. These strategies vary by retailer, resulting in a complex geography of provision by both format (e.g. hypermarket vs convenience) and brand. Larger stores are generally found in the suburbs and outskirts of large conurbations whereas smaller stores are generally found in central urban and rural locations, with the former driven by cost and availability of space and the latter driven by less concentrated demand. By brand, the largest multiple retailers have commanded greatest market share in cities and larger towns, whereas smaller retailers (including the Co-operative group and symbol groups, which are independent retailers that are a members of a larger organisation (Institute of Grocery Distribution, 2012)) have greater market share in more rural locations (Hood et al., 2016). Within these different broad location types, a spatial battle based on a specific target market also exists, with retailers targeting different sections of the population, ranging from the affluent customer base of ‘premium’ retailers such as Waitrose through to the traditional blue collar customer base of the discount retailers Aldi and Lidl (Thompson et al., 2012).

There is clearly the possibility that these strategies introduce some form of selection bias in where grocery retailers may choose to locate their stores, and since house prices or rental values could also be another way of making such differentials between neighbourhoods then this means that there is the potential for confounding to exist, which occurs when a factor (here the grocery retail provision) affects not only the treatment assignment (choice of store location) but also the outcomes (residential rental prices). There is therefore a potential for a non-random assignment of retail stores to neighbourhoods and this may introduce bias in any estimate derived from any traditional hedonic model, since a difference in the outcome between neighbourhoods with a retail band and those without may be caused by a factor that predicts probability of retail store location rather than the effect of the retail brand itself.

In this study the technique of propensity score matching is used to control for this potential selection bias (Rosenbaum & Rubin, 1983). Section two of the article contains a review of the literature on the relationship between retail provision and the housing market. The third section describes the method of propensity score matching and is followed by a section that introduces the data used in the modelling. Section five presents the results of the model, which are further discussed in the final section.

## Literature review

Much of the early house price modelling literature focussed on the monocentric urban model in which prices were hypothesised to decrease when moving away from the Central Business District (Chica Olmo, 1995; Orford, 2017). More recently, a wide range of location factors have been hypothesised (and found) to have an impact on house prices, in varying ways, at varying spatial scales (Orford, 2002). These studies attempt to capture the premium consumers are willing to pay for positive amenities (or are willing to pay to avoid disamenities). Within this change of focus, several studies have investigated the capitalisation of retail provision in housing prices, ranging from the general availability of retail opportunities or larger types of provision such as shopping centres, through to specific studies on localised grocery provision.

Therefore retail opportunities are an important aspect of the built environment and play a notable role in shaping the desirability of neighbourhoods (M. Jang & Kang, 2015), hence the possibility of capitalisation in housing and rental prices. On a general level, Barreca et al. (2020) found a positive correlation between retail vibrancy (measured as a combination of the provision of a variety of retail opportunities) and house listing price in Turin, Italy, although they later found an insignificant, but positive impact of the variable in a multivariate model. Moreover, Song and Sohn (2007) found positive house price capitalisation from greater accessibility to retail provision in Oregon, USA, whilst Law (2017) found a significant, small, positive effect of retail stores (multiple sectors) on house prices in London. Glaeser et al. (2001) highlights the wider benefit of increased retail provision, particularly in attracting customers and other businesses into a locality, with the consumer potentially benefitting from convenience, cost savings and increased choice.

Moving onto studies with a focus on a more specific aspect of retail provision reveals a wide range of research focused on the impact of shopping centres on housing prices. These include studies across a range of continents and contexts including in Florida (Sirpal, 1994), Quebec (Des Rosiers et al., 1996), Zurich (Stadelmann, 2010) and Nigeria (Aliyu et al., 2011), all of which found evidence of a positive association between provision of shopping centres (or a negative association to distance), highlighting the value placed on them by housing consumers. Importantly for this study, both Des Rosiers et al. (1996) and Sirpal (1994) found the positive impact to be conditioned by the size of the retail opportunity, suggesting the quantity (which could also be a proxy for quality through choice) is an important component of retail opportunities.

Of direct relevance to this research are those studies which focus on the premium in housing or rental prices associated with the provision (in quality and/or quantity) of grocery retailing. In North America, Cerrato Caceres and Geoghegan (2017) found the addition of a large grocery store very nearby (0–400 m) and nearby (400–800 m) was associated with 7% and 4% increases in house prices respectively in Massachusetts. Similarly Kang (2018) found appreciation of house prices with hypermarket grocery provision up to a certain distance in Seoul, South Korea, and Chiang et al. (2015) find a small positive association between house prices and convenience store (7-Eleven) provision close by (within 100 m) in Taipei Metropolis. These studies highlight the importance of both quantity and accessibility of available retail provision. Indeed Clarke et al. (2006) found that the largest driver of patronage to grocery stores was convenience and/or location, highlighting the importane of having a preferred brand available nearby with an associated premium envisaged for more desirable brands.

In a United Kingdom (UK) context, the national press often promotes the existence of a “Waitrose Effect”, said to result from the presence of the premium grocery retailer Waitrose increasing the price paid for housing in surrounding neighbourhoods (Alder, 2017; Burridge, 2018), with similar effects reported in other countries (Humphries & Rascoff, 2015). These types of studies are centred on variation in house prices depending on the brand of local grocery stores, but commonly suffer from methodological issues in either poorly defining the catchment of a store or, highly problematically, attributing the whole premium in house price to the presence of the grocery brand. Conversely, Clark et al. (2021) did control for a number of locational and structural factors when investigating the association between grocery brand and rental prices in the private rental submarket in England, finding differentials in association, with the greatest premium indeed associated with “Luxury” brands (e.g. Waitrose and M&S). In a different European context, Kurvinen and Wiley (2019) also used propensity score matching to investigate the impact of new retail provision on the sales price of individual properties in the Helsinki Metropolitan Area. There they found an impact (undifferentiated by brand) of + 1.5% within ½km of a new retail development and a more modest 0.6% within 1 km.

Consensus generally exists in the house price literature that the housing market is not a single entity, but rather made up of a number of subsets (submarkets) with their own characteristics and potential drivers of house price capitalisation, although how to define and identify each submarket remains a source of debate (Bangura & Lee, 2020). As with Clark et al. (2021), the focus here is on prices in the private rental sector, although here we focus on the whole of GB rather than just England. The private rental sector is a large submarket in GB and is increasing in size (Wilcox et al., 2017) and is therefore worthy of attention.

Homing in on the impact of one dimension of housing price requires controlling for a number of other covariates, each of which are generally either structural or locational in nature (Can, 1992; Singla & Bendigiri, 2019). Structurally, consensus has been reached on the main factors influencing price; notably that the age, type and size of the property are important. The latter has been specified by count of number of rooms (Ahmed et al., 2014; McCord et al., 2014) or by the floor area of the property (Löchl, 2010; McCluskey et al., 2013) in previous studies of rental price. Less consensus exists on which location variables to use, with a range of amenities and disamenities found to condition housing prices.

As noted by Clark et al. (2021), these include variables related to: the general character of the neighbourhood (Baron & Kaplan, 2010; Chica-Olmo et al., 2013; Heng et al., 1997; Kain & Quigley, 1970); amenities and services in a local area such as the quality and/or accessibility of schools (McCord et al., 2014; Zheng et al., 2016), parks (Del Giudice et al., 2017; Hoshino & Kuriyama, 2009) and transport infrastructure (Gibbons & Machin, 2005; Bohman & Nilsson, 2016; Dubé et al., 2018; Chica‐Olmo et al., 2019); the absence of specific disamenities such as pollution (Hanna, 2007) or crime (Oduwole & Eze, 2013); measures of accessibility related to both national and regional economic hubs (Adair et al., 2000; Waddell et al., 1993); and the local level of accessibility of the built environment (in space syntax studies such as Law (2017) and Xiao et al. (2016)).

Large grocery retailers employ location planning teams that consider a number of factors when choosing store locations. Many of these overlap with impacts on housing prices discussed so far, thus introducing potential selection bias into models of the impact of grocery amenities on housing costs. Many of these factors are encapsulated in their use of geodemographic systems capturing a range of demographic, economic or behavioural characteristics of potential customers and potential store location neighbourhoods (see Rains, (2020) for an example from Sainsbury’s location planning team). This also translates into varying brand affinity by different social, demographic and economic groups within the population (Pechey & Monsivais, 2015; Thompson et al., 2012). Additionally, housing variables themselves are known to form part of location strategy (Zentes et al., 2017), making recognition of this potential for selection bias particularly important.

In an attempt to overcome some of the issues of using an hedonic approach to this problem (Rosen, 1974), this paper uses an approach with the ability to control for selection bias, namely propensity score matching (Rosenbaum & Rubin, 1983).

## Methodology

Ideally, one approach to identify causal effects is to conduct a randomised-control trial experiment. Couched in a medical context, this is usually conducted by firstly allocating trial subjects randomly to either a treatment or a control (or placebo) group before the experiment begins. The impact of the treatment can then be estimated by the difference in the outcome of interest between the treated and control groups. Commonly however, only observational experiments are available, where the ‘experimenter’ has no control or influence on how or which trial subjects are allocated to either group. This impediment raises some issues, particularly the presence of confounding effects, which cannot be controlled for (Austin, 2011). The concern is that if the group of subjects that have undergone a treatment do not resemble the control group for some co-variates, then a bias is introduced into any outcome measures. There are various techniques available to address this concern. Of these, propensity score analysis is the technique adopted in this study.

### Propensity score analysis

The goal of propensity score analysis is to make the treatment and control groups similar when described by these co-variates (Rosenbaum & Rubin, 1983). The stages to this analysis are summarised here:

1. Choose the primary treatment effect
2. Estimate the propensity score weights
3. Evaluate the quality of the weights
4. Estimate the treatment effect

#### Choose the primary treatment effect

This treatment effect can usually be measured as either an average treatment effect on those who received the treatment (ATT) or as a population average treatment effect on both those treated and not treated (ATE).

#### Estimate the propensity score weights

The next step is to calculate a set of observation weights which are applied to subjects in the control group so that when the weighted summary statistics are calculated for each co-variate, they resemble the same statistics calculated on the treatment group. The first task is to estimate a model that provides the estimated probability that a subject will undergo the treatment, here denoted as $\hat{p}$, given the cofounders. Depending on the treatment effect desired, these probabilities are converted to observation weights; for ATT those in the treatment group are given a weight of 1.0 whilst those in the control group are weighted by $\hat{p}$/(1-$\hat{p}$), whilst for ATE, those treated are weighted by $\hat{p}$ and those in the control by 1/(1-$\hat{p}$). A point to note is that these weights are defined by not just one co-variate, but typically using an assortment of co-variates.

The method is largely agnostic to the manner in which the $\hat{p}$ is estimated, concentrating instead on the ability of the weights to make the treated and weighted control groups appear similar, see 3.1.3 below. Commonly a logistic model is used to derive these weights but more recently machine learning techniques have been found to be easier to apply and perform better in arriving at balancing scores (McCaffrey et al., 2013).

#### Evaluate the quality of the weights

Balance tables are available that show how well each weighted co-variate compares between the treatment and control group. The headline measure is the standardised difference between each group (Austin, 2009), which is a measure of agreement between means (an Effect Size, denoted as es). Also, rather than using a point estimate, measures are available to quantify and test the agreement between the distribution of values in the treatment and control group (from Kolmogorov–Smirnov, denoted as ks). In practice the quality is monitored using both criteria, each measured at their mean and maximum values (i.e. es.mean, es.max, ks.mean, ks.max) (Belitser et al., 2011).

#### Estimate the treatment effect

If this weighting exercise is a success, the treatment effect can be estimated by the difference in the weighted outcome of interest between the treated and control groups. Alternatively, rather than a simple difference in weighted outcomes, a weighted regression approach can be used. In this regression, the y variable is the outcome and the explanatory variable is a binary variable set to 0 if the subject is in the control group and 1 if they are in the treatment group, with the weights being those estimated at step 3.1.2. The parameter associated with this binary variable is the treatment effect, and its associated standard error gives an indication of the significance of the effect.

If any of the co-variates in Sect. 3.1.3 have a standardised effect size greater than 0.20, (a rule of thumb often cited in the literature, e.g. McCaffrey et al. (2013)), then they can be used as additional terms in the regression to produce doubly-robust estimates (Bang & Robins, 2005), which are consistent if either the propensity score weights are estimated correctly or the regression model used to estimate the weighted outcome is specified correctly.

#### Observations

In arriving at the balancing scores, the outcome of interest is not used, helping to avoid modelling decisions that are driven by the desired outcome. There are some drawbacks to propensity score analysis. The main one is that the effective sample size (ESS) of the control group is reduced as part of this process. Calculations are available that demonstrate how great this reduction is (Ridgeway et al., 2017), but if the initial unweighted control group is sufficiently large, this drawback is not so critical.

### Estimation software

The R software package (R Core Team, 2020) offers a number of techniques for estimating causal effects using propensity score analysis (Keller & Tipton, 2016; Leite, 2016). In this study the ‘Toolkit for Weighting and Analysis of Non-equivalent Groups’ (TWANG) approach will be used (Ridgeway et al., 2017). This approach uses Gradient Boosted Random Forests (GBRF) to derive the balancing scores that function as the weights. TWANG has been mainly applied in the medical (Pedersen et al., 2017; J. B. Jang et al., 2019) and public health domains (Cohen et al., 2013; Holliday et al., 2020; Patorno et al., 2013), although some (mainly in the United States of America) have applied propensity score analysis to the housing market (Aratani, 2011; Lim et al., 2014; Locke et al., 2017; Nanda & Ross, 2012; Paredes, 2011; Pollack et al., 2010).

Of the two alternative primary treatment effects to estimate, ATT and ATE, here the ATT will be estimated. This is because we are interested in what the effect on average rental prices is for those neighbourhoods that have the presence of a rental brand and we are not interested in the general effect, over the entire population, of a particular retail brand.

### Estimation framework

Here we fit nine models in total; one for each retail brand or grouping. The first model has as its treatment the presence or absence of a discounter brand (i.e. Aldi or Lidl) and uses the co-variates described in Sect. 4.3. The second model has the presence or absence of a freezer store as its treatment, and so on through the remaining brands.

Each of these nine TWANG models are estimated in two stages, the first stage is to estimate the propensity score weights and the second is to use these to estimate a weighted regression model. Optimality is monitored using all four criteria available in TWANG (es.mean, es.max, ks.mean, ks.max). The iteration which minimises each stopping criterion is adopted as the optimal solution for that criterion. The stopping criterion of the four that yields the largest ESS is chosen to derive the propensity scores.

For the weighted regression step a quasi-poisson formulation is used with clustered standard errors. The poisson model is adopted to reflect that the median rental prices are positively skewed with a potential for over-dispersion. This formulation means that any differences in rental prices are best interpreted as percentages. Standard errors are clustered by local government authority in which the neighbourhood belongs to account for the fact that such authorities set local property taxes and have some role in private rental regulation (Balchin & Rhoden, 2019).

## Data

### Rental data

Information on the level of neighbourhood rental prices is provided by the Urban Big Data Centre (2020) and is derived from the Zoopla Property listing web site (Zoopla, 2020). These data are aggregated to Middle Layer Super Output Area (MSOA) geography in England and Wales (Office for National Statistics, 2020a) and Intermediate Zones in Scotland (National Records of Scotland, 2020a) and provided as the mean rental listing price, the median listing price and a count of rental property listings. These rental data are provided by quarter of the year, from 2011 to 2016. Given the critical importance of this rental data to this study, the unit of analysis adopted are these MSOA’s/IZ’s, and it is these areas that define our neighbourhoods (there are 8,480 such neighbourhoods in total and MSOA’s have a mean size of 3,244 households and IZ’s have 1,855 households).

In these data the rental listing price is summarised as both a mean and a median. Whilst computationally the mean listing price has advantages, the median is much more robust to outliers, and there are some outliers present in these data. If we compare the ratio of the mean to the median, the mean is generally about 22% higher than the median, indicating a sizeable positive skew to these data. However, there are 3,203 neighbourhoods out of 8,480 (38%) where the mean rental price for at least one quarter is more than 33% greater than the median. The causes of these high means could be the presence of a particularly high value listing appearing on one rare occasion or errors in the listing price. To minimise the impact of these outliers, the outcome used for this study is the median rental listing price.

**Fig. 1:**
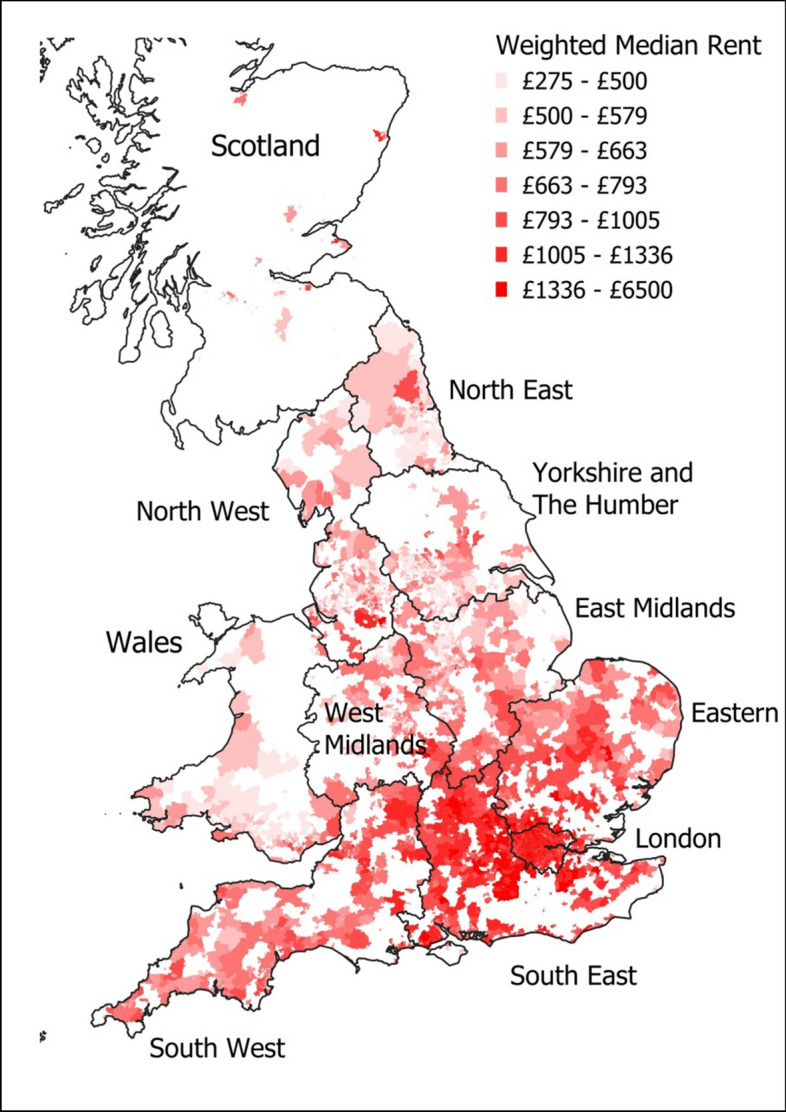
Distribution of weighted median rental prices

In appearance these data resemble (using a pseudo-medical analogy) partial panel data with multiple treatments and varying doses. The data is a partial panel, over a number of quarters, but for some neighbourhoods there are quarters with no data, since there were no properties listed for rent during those months,. The treatment applied to each neighbourhood is the presence of each retail brand (see 4.2), which can be applied in various doses, here measured as a count of the number of each retailers’ stores in the neighbourhood’s catchment. This structure is not amenable to direct analysis using currently available software, and therefore requires some re-structuring. Firstly the partial panel nature is collapsed into a single weighted median (using the R package matrixStats (Bengtsson, 2020)). Secondly the varying doses are collapsed to the presence or absence of a retail brand rather than a count of the brand. The multiple treatment aspect of the data is retained, allowing for an account of the competition effects between retail brands. Figure 1 maps the weighted median rental price for those neighbourhoods for which we have data. It can be seen here that there is good representation of urban neighbourhoods in these data, but in more rural areas, and much of Scotland, there are fewer neighbourhoods with rental data.

### Retail data

Information on the retail brand, location, size band and opening date is obtained from GEOLYTIX (2020a) using their open retail point data (GEOLYTIX, 2020b). The precise opening date is only available for stores opened post-2014. It can be assumed that stores with missing opening dates were open by the end of 2014, and to account for this imprecision, we restrict our analysis to the period that best overlaps our rental data; from 2015 quarter 1 to 2016 quarter 4. In reality, since there is a planning and construction lead time for retail stores, it is possible for a store to have an influence on the neighbourhood rental market before its actual recorded opening date. Such an impact is reported by Humphries and Rascoff (2015) and Kurvinen and Wiley (2019). This later study used a time horizon of 6 months to identify the effect of the temporal proximity of house sales to new retail developments, and report in Exhibit 5 that some positive effects where seen 6 months prior to a new retail development. To capture this dynamic for our study, a store is anticipated to have an effect on its neighbourhood for 6 months prior to its opening date.

Some aggregation is applied to create nine retail brands or groupings (Hood et al., 2016). Firstly there are separately the ‘Big-4’ retailers: ASDA, Morrisons, Sainsbury’s and Tesco. Then there are Aldi and Lidl, grouped together as ‘Discounters’. A number of retailers are similar in character, in that much of their produce is sold in freezers (Iceland, Farmfoods, Heron Foods and Jack Fulton) and these are grouped together as ‘Freezer’ brands. Both Budgens and Spar stores are similar in nature being convenience stores so are also grouped together. The Co-operative group is kept as a separate brand since it has its own substantial presence in the UK grocery market, serving both urban and rural locations. The final grouping is the ‘Premium’ retailers, Waitrose, Marks and Spencer and Booths. The geographic spread of these retail brands is shown in supplementary maps S1 to S9.

To determine which retail stores are in the catchment of each of our neighbourhoods, the crow-fly distance from the population weighted centroid of the neighbourhood to each retail store is calculated. A store is defined as being part of the neighbourhood if this distance is less than some threshold, which varies by four store sizes. To help determine what these thresholds might be, data from the National Travel Survey (Department for Transport 2020), table NTS0403, shows that people in Great Britain travel, on average, a road distance of 4 miles for shopping. However this is for all types of shopping and an analysis conducted for this study using record level participant’s data with shopping trips that have a main destination purpose of food shopping, shows that for such trips this road distance is a more modest 2 miles (3.2 km). With this in mind, the thresholds used in this study for the various stores sizes are given in Table 1 .

Here it should be noted that in general these crow-fly distances will be an underestimate of the actual road distance, meaning that for example, some large stores with a road distance greater than say 4.8 km may have a crow-fly distance less than 4.8 km and thus be included in the neighbourhood’s catchment. This buffer approach was adopted in preference to an approach that only identifies stores within the neighbourhood - given that our neighbourhoods are small, it is feasible that shoppers would consider stores that are outside their own neighbourhood, but within some travel distance.

### Co-variate data

As highlighted previously, studies have identified a range of factors that have been shown to have an influence on private rental housing and these can be summarised in terms of the nature of the property itself (e.g. its size or amenities), the nature of the neighbourhood (e.g. levels of affluence or crime), or access to facilities (e.g. transport hubs, greenspace and also retail options). Here we will describe co-variate data that captures each of these influences.

#### Property data

Since the data on rental listing prices is aggregated from many property listings, no accurate notion of the type or size of property is possible. However, it may be useful to know the mix of rental properties in each neighbourhood, and this information is available from the 2011 census. Tables DC4402EW (England and Wales) and LC4402SC (Scotland) provide the percentage mix of private rental property types in the neighbourhood, here categorised as detached, semi-detached, terraced and flats (apartments). Corresponding tables LC4404EW and LC4404SC provide information on the percentage mix of property sizes, measured as the number of rooms (excluding bathrooms, toilets, halls or landings, or rooms that can only be used for storage) and categorised as 1 room, 2 or 3 rooms, 4 or 5 rooms, and 6 or more rooms.

#### Affluence

Capturing the affluence of residents in a neighbourhood can be a challenge since there is no accepted definition of what affluence is precisely. It could simply be derived from the average income of households in the neighbourhood, to something that is a composite of various indicators of prosperity. Whilst small area estimates of income (Scottish Government/Riaghaltas na h-Alba, 2017; Office for National Statistics, 2020b) and deprivation (Llywodraeth Cymru/Welsh Government, 2019; Ministry of Housing, 2019; Scottish Government/Riaghaltas na h-Alba, 2020) are available, they are inconsistently derived across the three home nations of GB making them unsuitable for this analysis. Instead the commercial ACORN (CACI, 2020) classification of postcodes for the whole of the UK is used to measure neighbourhood affluence. This classification allocates postcodes a label based on knowledge of local demographics, social factors, population, and consumer behaviour. Here the top level with six categories is used: ‘Affluent Achievers’, ‘Rising Prosperity’, ‘Comfortable Communities’,’Financially Stretched’,’Urban Adversity’, and’Not Private Households’. The percentage mix of postcodes, and hence ACORN categories, within each neighbourhood are calculated (using a lookup of postcodes to neighbourhoods (Office for National Statistics, 2018)), plus an additional category is created for postcodes that do not have an ACORN category.

#### Neighbourhood environment

Whilst the affluence measure tells us something about the nature of the residents in a neighbourhood, some additional information on the physical character of the neighbourhood would be insightful. Such aspects include the access to leisure opportunities, health services, green space and the level of air quality or crime. In this study a composite indicator ‘Access to Healthy Assets and Hazards’ (AHAH) is used which scores the healthiness of areas on four domains (Green et al., 2018). These domains are: retail, for example the presence of gambling outlets, pubs and fast-food retailers; health, including the ease of access to general practitioner surgeries, chemists and hospitals; environment, measured by amount of green and blue space; and the quality of the air. These scores are provided at a smaller population geography than our neighbourhoods, that of lower level super output areas (LSOA’s) or data zones in Scotland (DZ). Since LSOA’s/DZ’s nest within our neighbourhoods, to arrive at a score for our neighbourhoods, the constituent LSOA’s/DZ’s 2015/2016 mid-year population estimates are used to calculate a population weighted score (Office for National Statistics, 2020c; National Records of Scotland, 2020b). Larger scores represent neighbourhoods that have poorer health-related environments.

#### Access to facilities

London is regarded as a ‘hot-spot’ for the property market (Office for National Statistics, 2020d). To capture this gradient in property prices a crow-fly distance of the neighbourhood’s population weighted centroid from central London is measured, separately as a distance north/south and east/west. This distance is logged so that the further from London, the differential effect of this term for a given change in distance diminishes. The logarithm of the crow-fly distance to the nearest large employment centre (an LSOA/DZ with 5000 or more jobs) is used to reflect ease of access to employment opportunities (Department for Transport, 2015). The final facility included is the crow-fly distance to the nearest mainline railway or underground station, to capture the availability of travel options.

#### A sense check on the co-variates

To ensure that these co-variates have the potential to influence the outcome, a regression model is estimated, where the outcome is the neighbourhood rental price and the regressors are each of these co-variates. This approach is not ideal, since as discussed at the start, any estimates from such a model may be subject to selection bias, but notwithstanding this, an expectation is that these co-variates will be of the correct sign and be significant.

### Retail competition

A final consideration is to ensure that when investigating the impact of a particular brand, and looking for similar neighbourhoods, proper account is taken of all the retail options for the neighbourhood. Thus the outcome is measured against areas with a similar retail mix of competing brands. For example, if a discounter retailer tends to locate in neighbourhoods that also contain a considerable number of freezer type stores but few premium stores, then it is important that due consideration is given to this aspect by the up-weighting of similar neighbourhoods with freezer type stores and a down-weighting of neighbourhoods with premium stores. In effect here we are controlling for any spatial heterogeneity and interactions in store locations by introducing competitor stores’ presence as co-variates.

### Data Availability

In designing this study, we have tried to use open sources of data. The rental and retail data are provided as download links from their respective web sites. Almost all of the co-variate data are also freely available with the exception of the ACORN classification which is commercial, but available to accredited academics (UK Data Service, 2017).

## Results

There is rental listing data for at least one quarter for 5221 of the 8480 neighbourhoods in GB. The ‘Statistic’ column in Table 2 shows summary statistics for each of the balancing co-variates. By way of explanation: the average rent is £867; the mean proportion of detached properties in each neighbourhood is 11.2%; there is a mean of 1.9% of properties with one room; on average, neighbourhoods have 15.7% of their postcodes classified as Affluent Achievers; the mean composite AHAH score is 22.69; and the mean (logged) east/west distance from central London is 3.93.

**Table 1:** Catchment thresholds for each store size

Size	Store area	Threshold crow fly-distance
Small	Less than 280m^2^	800 m
Medium small	280m^2^ to 1400m^2^	1600 m
Medium large	1400m^2^ to 2800m^2^	3200 m
Large	Greater than 2800m^2^	4800 m

The hedonic regression results in Table 2 show that neighbourhoods with higher proportions of semi-detached and terraced rental properties have lower rents, and those with larger percentages of properties with more rooms have higher rents. As affluence decreases then so do the rents. Surprisingly as the unheathiness of the neighbourhood increases then so do the rental values, but this effect is only significant at the 10% level. Greater distances from London, employment centres and railway/underground stations all result in lower rents. With the exception of the AHAH parameter (which is insignificant at the 95% level), these parameters have expected signs.

**Table 2:** Parameter estimates from hedonic investigation

Co-variate	Summary	Estimate	se	t-ratio	Pr(> 0)
Intercept	£867	7.1292	0.3425	20.82	0.0000***
Detached (%)	11.2%				
Semi-detached (%)	22.4%	− 0.0041	0.0018	− 2.37	0.0178*
Terraced (%)	26.1%	− 0.0055	0.0016	− 3.47	0.0005***
Flats (%)	40.4%	0.0016	0.0016	1.01	0.3102
1 room (%)	1.9%				
2 or 3 rooms (%)	23.3%	0.0039	0.0029	1.36	0.1742
4 or 5 rooms (%)	51.0%	0.0065	0.0029	2.23	0.0260*
6 rooms (%)	23.8%	0.0144	0.0033	4.30	0.0000***
Affluent Achievers (%)	15.7%				
Rising Prosperity (%)	7.2%	− 0.0012	0.0008	− 1.62	0.1061
Comfortable Communities (%)	17.2%	− 0.0034	0.0007	− 5.12	0.0000***
Financially Stretched (%)	13.9%	− 0.0033	0.0008	− 3.91	0.0001***
Urban Adversity (%)	12.1%	− 0.0030	0.0007	− 4.44	0.0000***
Not Private Households (%)	6.5%	− 0.0057	0.0010	− 5.69	0.0000***
Not Found (%)	27.4%	− 0.0022	0.0005	− 4.36	0.0000***
AHAH (score)	22.69	0.0028	0.0016	1.74	0.0811
East or West of London (log km)	3.93	− 0.1025	0.0107	− 9.60	0.0000***
North or South of London (log km)	3.91	− 0.1023	0.0091	− 11.18	0.0000***
Distance to large employment (log km)	1.13	− 0.0394	0.0079	− 5.00	0.0000***
Distance to railway/underground (log km)	0.34	− 0.0250	0.0063	− 4.00	0.0001***

Note Signif. codes: 0 ‘***’ 0.001 ‘**’ 0.01 ‘*’ 0.05 ‘.’ 0.1 ‘ ’1

Turning to the propensity score analysis results, the outcome of the estimation is reported in supplementary figures S1 to S9 using the standard graphs provided by the TWANG package and the balance tables from the propensity score weightings are reported in supplementary Tables S1 to S9. The GBRF models have primarily achieved good balancing scores, with only some co-variates in the models for Sainsbury’s and the Premium group of retailers showing poor balance for some co-variates (with a standardised effect size greater than 0.20), and as a consequence these co-variates are used for the doubly-robust estimation for these two models (see the footnote to Table 5).

To see the impact on the sample sizes of the propensity score analysis, the ESS for each of the stopping criteria are given in Table 3. The process of deriving the propensity score has caused a substantial reduction in some of the sample sizes, and reflect the challenge required to make the neighbourhoods in the control group appear like those in the treatment group. The Spar/Budgens group has the largest unweighted sample size, with 4,124 control neighbourhoods without such a store in their catchment, and this sizeable number of control areas has made the process of deriving propensity score weights a relatively easy task, hence the corresponding large ESS when mean effect size (ES Mean) is used as the stopping criterion. By contrast, there are few neighbourhoods without a Tesco store in their catchment, meaning that using these 1,150 available control areas, the calculation of weights has been a challenge and the ESS is considerably reduced, to a percentage in the low to mid-teens. Whilst there is no set rule for choosing one stopping criterion over another, a desire to use the maximum ESS possible to provide the estimated control group outcome is a sensible rule. So for each retail brand or grouping, the stopping criteria that has the largest ESS, and is therefore the one used to derive the propensity score weights, is highlighted in bold.

**Table 3:** Effective sample sizes from each stopping criteria (ESS and (% of N)) with the criteria with the largest ESS shown in bold

Retailer/Grouping	ES Mean	ES Max	KS Mean	KS Max	N Control
Discount	977.3 (37.1%)	981.0 (37.2%)	1003.4 (38.1%)	**1006.7 (38.2%)**	2635
Freezer	632.9 (24.2%)	607.2 (23.2%)	633.1 (24.2%)	**760.0 (29.0%)**	2618
Budgens/Spar	**3353.4 (81.3%)**	3130.9 (75.9%)	3338.4 (81.0%)	3254.5 (78.9%)	4124
Co-op	**1536.5 (60.5%)**	1534.2 (60.4%)	1530.2 (60.2%)	1535.1 (60.4%)	2540
ASDA	**636.5 (34.2%)**	535.2 (28.7%)	613.4 (32.9%)	599.7 (32.2%)	1863
Morrisons	1079.4 (47.3%)	**1081.0 (47.4%)**	1079.8 (47.4%)	1061.6 (46.6%)	2280
Sainsbury's	181.5 (11.5%)	203.4 (12.9%)	213.2 (13.5%)	**239.8 (15.2%)**	1574
Tesco	200.6 (17.4%)	227.1 (19.7%)	**235.8 (20.5%)**	200.1 (17.4%)	1150
Premium	717.5 (26.5%)	644.7 (23.8%)	765.4 (28.3%)	**828.3 (30.6%)**	2709

N Control is the number of neighbourhoods in the control group, i.e. those that do not have the retailer or the group in their catchment

As outlined in Sect. 3.3 the primary treatment effects are obtained in two stages, the first stage is to derive weights for our data, based on the propensity scores from GBRF models (as described above). These weights are then used in a regression equation where the y-variate is the weighted median rental price and the explanatory variables are, firstly a binary variable to indicate if the observation (i.e. neighbourhood) has the retailer present (i.e. is ‘treated’), and secondly (in a doubly –robust framework) any co-variates with sizeable standardised differences. It is the regression estimate from the binary variable in this formulation that provides the estimate of the percentage impact of the presence of the retailer on rental prices. To illustrate this second stage, the output from the weighted quasi-poisson regression Eq. (1) for the Premium brand of retail stores is shown in Table 4, where the estimated impact of having a Premium store present in the neighbourhood is significant at an estimated value of 10.05%, whilst also controlling for the proportion of flats in the neighbourhood and two of the ACORN the designations.

1 $$y_{i} = \alpha + \beta T_{i} + \mathop \sum \limits_{{j \in co - {\text{var}} iates }} \gamma_{j} C_{j,i}$$

where y_i_ is the weighted median rental price for neighbourhood i; α is the intercept; T_i_ = 0 if the retailer does not have a store in neighbourhood i’s catchment; = 1 if the retailer does have a store in neighbourhood i’s catchment; β the estimated percentage impact of the presence of the retailer on the weighted median rental price; C_i_ are a series of j co-variates whose standardised differences are greater than 0.20; γ_j_ are the estimate impacts of the co-variate on the weighted median rental price.

**Table 4:** Regression output for Premium brand stores

Regression term	Estimate	Std. Error	t-vale	Pr(> t)
Intercept	6.4774	0.0662	97.84	0.0000
Premium presence	0.1005	0.0299	3.36	0.0008
Flats	0.0037	0.0012	3.11	0.0018
ACORN: Rising Prosperity	0.0111	0.0010	10.86	0.0000
ACORN: Comfortable Communities	0.0005	0.0014	0.38	0.7007

Running such models for all nine retail groupings provides the estimates listed in Table 5, ordered by the estimated percentage impact of the retailer on rental prices.

**Table 5:** Parameter estimates form the propensity score analysis

Retail brand/Group	Summary^1^	Estimate	se	t-ratio	Pr(> 0)
Freezer	50.0%	− 5.13%	0.0353	− 1.455	0.1458
ASDA	64.0%	− 3.38%	0.0496	− 0.682	0.4953
Discounter	50.0%	− 1.64%	0.0268	− 0.610	0.5421
Budgens/Spar	21.0%	− 1.33%	0.0342	− 0.389	0.6970
Morrisons	56.0%	− 0.59%	0.0472	− 0.125	0.9008
Sainsbury's ^2^	70.0%	1.56%	0.0291	0.535	0.5925
Tesco	78.0%	3.07%	0.0668	0.459	0.6463
Co-op	51.0%	3.72%	0.0285	1.302	0.1929
Premium ^3^	48.0%	10.05%	0.0299	3.362	0.0008***

^1^The percentage of neighbourhood with a store of this brand in its catchment

^2^A doubly-robust estimate that includes: Flats; One Room; East/West of London; Rising Prosperity; and the AHAH score

^3^A doubly-robust estimate that includes: Flats; Rising Prosperity; Comfortable Communities

The Freezer group of retailers has the largest negative (but insignificant) impact on rental prices in those neighbours with such a retailer. Further negative, and insignificant, impacts are estimated for ASDA, the Discounters, Budgens/Spars and Morrisons. However, for Sainsbury’s, Tesco, the Co-operative group and the Premium retailers, the impacts are positive, but only positive and significant for the Premium group. The ordering in this table reflects a general understanding of the perceived ‘attractiveness’ of retail brands in the UK (see Thompson et al. (2012) who used opinion poll data to profile UK retailers), although other considerations, such as ‘value for money’ might produce different rankings. These results indicate that whilst there are some impacts of retail brand on residential private rents, the effect is not generally significant, except for the Premium grouping of retailers where the effective is positive, large and significant.

## Discussion

This article has estimated the impact of the presence of retail brands on the average rental price for residential properties in neighbourhoods. We found a significant, large, positive effect for the presence of a Premium grocery retailer on residential rental prices. Whilst impacts in line with previous research into brand attractiveness (although not concerning housing prices) were found for other brands/store types (see Thompson et al., 2012), these were not significant. In terms of scale, compared to a related study of the Finnish housing market by Kurvinen and Wiley (2019), we find larger impacts (both positive and negative, although insignificant for many brands), which is perhaps to be expected when capturing grocery availability by a heterogeneous collection of brands, rather than by undifferentiated presence. The GB grocery retail market is also characterised by far greater choice in retailer than the Finnish market, which is oligopolistic and dominated by two retail brands, together controlling nearly 83% of the market between them (Finnish Grocery Trade, 2020). Also here we have a different housing market, rental rather than sales.

Methodologically, the technique of propensity score matching has been used to control for selection-bias where the outcome of interest here, rental prices, may also influence where brands chose to locate. In deriving the propensity scores for this method, good balance was achieved for many of the co-variates used, providing confidence that the comparison in outcome between the treated and ‘control’ group is valid. For some brands and for some co-variates where balance was not achieved a doubly-robust approach was used to provide consistent estimators.

We also had to simplify the structure of the data to make it amenable to analysis. No software package that we are aware of is able to recognise the complex nature of a partial panel multiple dose treatments that best typifies these data. In simplifying these data for analysis, we have also reduced the influence of outlying observations making the models estimated more robust.

Growth in grocery retail e-commerce for some retail brands possibly means that store access is becoming less important for shoppers, but ‘geography to delivery’ networks also exist and studies have shown that these can reflect the existing store networks for many retailers (Clarke et al., 2015). Even in an era of ‘omni-channel retailing’ store location can still be critical, especially so if these services are fulfilled by a ‘pick and pack in store’ on-line distribution strategy (e.g. Sainsbury’s), where on-line orders are sourced from in store inventory; or by click-and-collect, where on-line orders are picked in store by a member of staff, but collected by the customer (Mou et al., 2018). These dynamics could be further explored in future work on house and rental prices, both taking into account that different consumer types (e.g. by age) exhibit varying rates of uptake/dependence on different e-commerce services (Hood et al., 2020), and the variable impact of location on retail footfall rates depending on context, as exemplified by different patterns of home working during the Covid-19 pandemic (e.g. see Kirk and Gutiérrez (2020)).

Whilst compiling the data set for this study, the spatial relationship between co-variates has been of paramount importance, be it the establishment of catchments for neighbourhoods or various proximity measures (e.g. to Central London or transport access points), the regression model used has not explicitly recognised these proximities (as Montero-Lorenzo et al. (2009) did in a study of commercial property rental prices in Toledo, Spain). There are such models available, for example spatial error or lagged models (Anselin, 2009) or geographically weighted regression (GWR) (Brunsdon et al., 1998), that incorporate the spatial arrangement of terms within a regression model. However it is unclear how, especially in terms of GWR, the spatial and propensity score weights would interact. Thus in light of the current lack of understanding of this issue, we have not added this level of complexity to our analysis.

The findings of this research allow local planning officers to further understand the impact of planning applications, particularly of Premium brand retailers, on the built environment and specifically the demand and affordability of housing for rent. Higher rental prices on locations where a Premium retailer establishes a presence is consistent with the process of retail gentrification (Hubbard, 2018), but one that does not just apply to residential property sales prices but also demonstrably applies here for rents too. Whilst those who are asset rich would welcome this boost to their capital and income, this process can have negative effects, pricing out established families and communities (Paccoud, 2017). Additionally, King (2018) identified (also using propensity score matching) a link between food insecurity and housing instability, and it is arguably the case for GB that the private rented housing market is the most insecure housing market (Clarke et al., 2017), possibly compounding already challenging housing issues for private rental tenants.

## Supplementary Information

Below is the link to the electronic supplementary material.

Supplementary file1 (DOCX 52 KB)

Supplementary file2 (PDF 1017 KB)

Supplementary file3 (PDF 2462 KB)

## Data Availability

The data used in this study are available to researchers on application from various sources as referenced in the article.

## Declarations

### Code availability

The R code for the analysis can be obtained on request from the authors.

## References

[CR2] Adair A, McGreal S, Smyth A, Cooper J, Ryley T (2000). House prices and accessibility: The testing of relationships within the Belfast urban area. Housing Studies.

[CR3] Ahmed S, Rahman MM, Islam S (2014). House rent estimation in Dhaka city by multi layer perceptions neural network. International Journal of u-and e-Service, Science and Technology.

[CR4] Alder, D. (2017). The Waitrose effect: boom times for homeowners but evictions for tenants. https://www.theguardian.com/inequality/2017/oct/02/the-waitrose-effect-boom-times-for-homeowners-but-evictions-for-tenants.

[CR5] Aliyu, A. A., Kasim, R., & Martin, D. (2011). Effect of Kasuwan Laushi Super Market on Surrounding Residential Accommodations in Bauchi Metropolis, Nigeria. In *Proceedings of International Conference on Environment and Industrial Innovation (ICEII 2011).*

[CR6] Anselin L (2009). Spatial regression. The SAGE Handbook of Spatial Analysis.

[CR7] Aratani Y (2011). Socio-demographic variations of homeowners and differential effects of parental homeownership on offspring's housing tenure. Housing Studies.

[CR8] Austin PC (2009). Using the standardized difference to compare the prevalence of a binary variable between two groups in observational research. Communications in Statistics-Simulation and Computation.

[CR9] Austin PC (2011). An introduction to propensity score methods for reducing the effects of confounding in observational studies. Multivariate Behav Res.

[CR10] Balchin, P., & Rhoden, M. (2019). *Housing policy: an introduction*: Routledge.

[CR11] Bang H, Robins JM (2005). Doubly robust estimation in missing data and causal inference models. Biometrics.

[CR12] Bangura M, Lee CL (2020). House price diffusion of housing submarkets in Greater Sydney. Housing Studies.

[CR13] Baron, M. G., & Kaplan, S. (2010). *The impact of ‘studentification’ on the rental housing market.* Jönköping, Sweden: Paper presented at the 50th Congress of the European Regional Science Association: “Sustainable Regional Growth and Development in the Creative Knowledge Economy”.

[CR14] Barreca A, Curto R, Rolando D (2020). Urban vibrancy: An emerging factor that spatially influences the real estate market. Sustainability.

[CR15] Belitser SV, Martens EP, Pestman WR, Groenwold RH, de Boer A, Klungel OH (2011). Measuring balance and model selection in propensity score methods. Pharmacoepidemiology and Drug Safety.

[CR16] Bengtsson, H. (2020). matrixStats: Functions that apply to rows and columns of matrices (and to Vectors). R package version 0.56.0.

[CR17] Black, C., Broadstock, D. C., Collins, A., & Hunt, L. C. (2007). The derived demand for traffic at food superstores in the UK: A semi-parametric regression approach. *International Journal of Transport Economics/Rivista internazionale di economia dei trasporti*, 403–427.

[CR18] Bohman H, Nilsson D (2016). The impact of regional commuter trains on property values: Price segments and income. Journal of Transport Geography.

[CR20] Brunsdon C, Fotheringham S, Charlton M (1998). Geographically weighted regression. Journal of the Royal Statistical Society: Series D (The Statistician).

[CR21] Burridge, N. (2018). High-end supermarkets boost house prices by £40,000. https://www.zoopla.co.uk/discover/property-news/top-end-supermarkets-boost-house-prices-by-40-000/#Bqzuhi5j8YgMCTA7.97.

[CR22] Ministry of Housing, C. L. G. (2019). English indices of deprivation 2019. https://www.gov.uk/government/statistics/english-indices-of-deprivation-2019.

[CR23] CACI (2020). The smarter consumer classification. https://acorn.caci.co.uk/.

[CR24] Can A (1992). Specification and estimation of hedonic housing price models. Regional Science and Urban Economics.

[CR25] Cerrato Caceres B, Geoghegan J (2017). Effects of new grocery store development on inner-city neighborhood residential prices. Agricultural and Resource Economics Review.

[CR26] Chiang Y-H, Peng T-C, Chang C-O (2015). The nonlinear effect of convenience stores on residential property prices: A case study of Taipei Taiwan. Habitat International.

[CR27] Chica Olmo J (1995). Spatial estimation of housing prices and locational rents. Urban Studies.

[CR28] Chica-Olmo J, Cano-Guervos R, Chica-Olmo M (2013). A coregionalized model to predict housing prices. Urban Geography.

[CR29] Chica-Olmo J, Cano-Guervos R, Tamaris-Turizo I (2019). Determination of buffer zone for negative externalities: Effect on housing prices. The Geographical Journal.

[CR30] Clark S, Hood N, Birkin M (2021). A hedonic model of the association between grocery brand provision and residential rental prices in England. International Journal of Housing Markets and Analysis.

[CR31] Clarke A, Hamilton C, Jones M, Muir K (2017). Poverty, evictions and forced moves.

[CR32] Clarke G, Thompson C, Birkin M (2015). The emerging geography of e-commerce in British retailing. Regional Studies, Regional Science.

[CR33] Clarke I, Hallsworth A, Jackson P, De Kervenoael R, Del Aguila RP, Kirkup M (2006). Retail restructuring and consumer choice 1. Long-term local changes in consumer behaviour: Portsmouth. 1980–2002. Environment and Planning A.

[CR34] Cohen DA, Lapham S, Evenson KR, Williamson S, Golinelli D, Ward P (2013). Use of neighbourhood parks: Does socio-economic status matter? A four-city study. Public Health.

[CR35] Del Giudice V, De Paola P, Manganelli B, Forte F (2017). The monetary valuation of environmental externalities through the analysis of real estate prices. Sustainability.

[CR1] Department for Transport. (2015). Accessibility Statistics: Travel time calculation methodology. https://assets.publishing.service.gov.uk/government/uploads/system/uploads/attachment_data/file/474271/accessibility-statistics-travel-time-calculation-methodology.pdf.

[CR36] Department for Transport. (2020). National Travel Survey: 2019. https://www.gov.uk/government/statistics/national-travel-survey-2019.

[CR37] Des Rosiers F, Lagana A, Thériault M, Beaudoin M (1996). Shopping centres and house values: An empirical investigation. Journal of Property Valuation and Investment.

[CR38] Dubé J, Legros D, Devaux N (2018). From bus to tramway: Is there an economic impact of substituting a rapid mass transit system? An empirical investigation accounting for anticipation effect. Transportation Research Part a: Policy and Practice.

[CR39] Finnish Grocery Trade. (2020). Finnish Grocery Trade 2020. https://www.pty.fi/fileadmin/user_upload/tiedostot/Julkaisut/Vuosijulkaisut/EN_2020_vuosijulkaisu.pdf.

[CR40] GEOLYTIX. (2020a). Better decisions where location matters. https://geolytix.co.uk/#.

[CR41] GEOLYTIX. (2020b). GEOLYTIX Retail Points User Guide.

[CR42] Gibbons S, Machin S (2005). Valuing rail access using transport innovations. Journal of Urban Economics.

[CR43] Glaeser EL, Kolko J, Saiz A (2001). Consumer City. Journal of Economic Geography.

[CR44] Green MA, Daras K, Davies A, Barr B, Singleton A (2018). Developing an openly accessible multi-dimensional small area index of ‘Access to Healthy Assets and Hazards’ for Great Britain, 2016. Health and Place.

[CR45] Hanna BG (2007). House values, incomes, and industrial pollution. Journal of Environmental Economics and Management.

[CR46] Heng L, Li V, Skitmore M (1997). Comparative study of analytical rental model and statistical models for predicting house rental levels. Building and Environment.

[CR47] Holliday SB, Troxel W, Haas A, Ghosh-Dastidar MB, Gary-Webb TL, Collins R (2020). Do investments in low-income neighborhoods produce objective change in health-related neighborhood conditions. Health and Place.

[CR48] Hood N, Clarke G, Clarke M (2016). Segmenting the growing UK convenience store market for retail location planning. The International Review of Retail, Distribution and Consumer Research.

[CR49] Hood N, Urquhart R, Newing A, Heppenstall A (2020). Sociodemographic and spatial disaggregation of e-commerce channel use in the grocery market in Great Britain. Journal of Retailing and Consumer Services.

[CR50] Hoshino T, Kuriyama K (2009). Measuring the benefits of neighbourhood park amenities: Application and comparison of spatial hedonic approaches. Environmental and Resource Economics.

[CR51] Hubbard, P. (2018). *Retail gentrification*. Edward Elgar Publishing.

[CR52] Humphries, S., & Rascoff, S. (2015). *Zillow Talk: The New Rules of Real Estate*: Grand Central Publishing.

[CR53] Institute of Grocery Distribution. (2012). Symbol groups: Market overview. https://www.igd.com/articles/article-viewer/t/symbol-groups-market-overview/i/15516.

[CR54] Jang JB, Schuler MS, Evans-Polce RJ, Patrick ME (2019). College attendance type and subsequent alcohol and marijuana use in the US. Drug and Alcohol Dependence.

[CR55] Jang M, Kang C-D (2015). Retail accessibility and proximity effects on housing prices in Seoul, Korea: A retail type and housing submarket approach. Habitat International.

[CR56] Kain JF, Quigley JM (1970). Evaluating the quality of the residential environment. Environment and Planning A.

[CR57] Kang C-D (2018). Valuing spatial access to types of retail and effects on the housing price in Seoul, Korea. Journal of Urban Planning and Development.

[CR58] Keller B, Tipton E (2016). Propensity score analysis in R: A software review. Journal of Educational and Behavioral Statistics.

[CR59] King C (2018). Food insecurity and housing instability in vulnerable families. Review of Economics of the Household.

[CR120] Kirk, A., & Gutiérrez, P. (2020). How Britain’s high streets are recovering after lockdown-visual analysis. The Guardian on-line. https://www.theguardian.com/world/ng-interactive/2020/aug/31/how-britains-high-streets-are-recovering-after-lockdown-visual-analysis.

[CR60] Kurvinen A, Wiley J (2019). Retail development externalities for housing values. Journal of Housing Research.

[CR61] Law S (2017). Defining Street-based Local Area and measuring its effect on house price using a hedonic price approach: The case study of Metropolitan London. Cities.

[CR62] Leite, W. (2016). *Practical propensity score methods using R*: Sage Publications.

[CR63] Lim S, Marcus SM, Singh TP, Harris TG, Levanon Seligson A (2014). Bias due to sample selection in propensity score matching for a supportive housing program evaluation in New York City. PLoS ONE.

[CR64] Llywodraeth Cymru/Welsh Government. (2019). Welsh Index of Multiple Deprivation. https://statswales.gov.wales/Catalogue/Community-Safety-and-Social-Inclusion/Welsh-Index-of-Multiple-Deprivation.

[CR65] Löchl, M. (2010). *Application of spatial analysis methods for understanding geographic variation of prices, demand and market success*. ETH Zurich.

[CR66] Locke CM, Butsic V, Rissman AR (2017). Zoning effects on housing change vary with income, based on a four-decade panel model after propensity score matching. Land Use Policy.

[CR67] McCaffrey DF, Griffin BA, Almirall D, Slaughter ME, Ramchand R, Burgette LF (2013). A tutorial on propensity score estimation for multiple treatments using generalized boosted models. Statistics in Medicine.

[CR68] McCluskey W, McCord M, Davis P, Haran M, McIlhatton D (2013). Prediction accuracy in mass appraisal: A comparison of modern approaches. Journal of Property Research.

[CR69] McCord M, Davis P, Haran M, McIlhatton D, McCord J (2014). Understanding rental prices in the UK: A comparative application of spatial modelling approaches. International Journal of Housing Markets and Analysis.

[CR70] Montero-Lorenzo J-M, Larraz-Iribas B, Páez A (2009). Estimating commercial property prices: An application of cokriging with housing prices as ancillary information. Journal of Geographical Systems.

[CR71] Mou S, Robb DJ, DeHoratius N (2018). Retail store operations: Literature review and research directions. European Journal of Operational Research.

[CR72] Nanda A, Ross SL (2012). The impact of property condition disclosure laws on housing prices: Evidence from an event study using propensity scores. The Journal of Real Estate Finance and Economics.

[CR73] Oduwole H, Eze H (2013). A hedonic pricing model on factors that influence residential apartment rent in Abuja satellite towns. Mathematical Theory and Modeling.

[CR74] Office for National Statistics. (2020a). Census Geography. https://www.ons.gov.uk/methodology/geography/ukgeographies/censusgeography.

[CR75] Office for National Statistics. (2020c). Population estimates by output areas, electoral, health and other geographies, England and Wales Statistical bulletins https://www.ons.gov.uk/peoplepopulationandcommunity/populationandmigration/populationestimates/bulletins/annualsmallareapopulationestimates/previousReleases.

[CR76] Office for National Statistics. (2020b). Income estimates for small areas, England and Wales. https://www.ons.gov.uk/employmentandlabourmarket/peopleinwork/earningsandworkinghours/datasets/smallareaincomeestimatesformiddlelayersuperoutputareasenglandandwales.

[CR77] Office for National Statistics. (2020d). Private rental market summary statistics in England: April 2019 to March 2020. https://www.ons.gov.uk/peoplepopulationandcommunity/housing/bulletins/privaterentalmarketsummarystatisticsinengland/april2019tomarch2020.

[CR78] Orford, S. (2017). *Valuing the built environment: GIS and house price analysis*: Routledge.

[CR79] Orford S (2002). Valuing locational externalities: A GIS and multilevel modelling approach. Environment and Planning B: Planning and Design.

[CR80] Paccoud A (2017). Buy-to-let gentrification: Extending social change through tenure shifts. Environment and Planning A.

[CR81] Paredes DJ (2011). A methodology to compute regional housing price index using matching estimator methods. The Annals of Regional Science.

[CR82] Patorno, E., Grotta, A., Bellocco, R., & Schneeweiss, S. (2013). Propensity score methodology for confounding control in health care utilization databases. *Epidemiology, Biostatistics and Public Health, 10*(3).

[CR83] Pechey R, Monsivais P (2015). Supermarket choice, shopping behavior, socioeconomic status, and food purchases. American Journal of Preventive Medicine.

[CR84] Pedersen ER, Parast L, Marshall GN, Schell TL, Neighbors C (2017). A randomized controlled trial of a web-based, personalized normative feedback alcohol intervention for young-adult veterans. Journal of Consulting and Clinical Psychology.

[CR86] Pollack CE, Griffin BA, Lynch J (2010). Housing affordability and health among homeowners and renters. American Journal of Preventive Medicine.

[CR87] Office for National Statistics. (2018). Postcode to Output Area to Lower Layer Super Output Area to Middle Layer Super Ouput Area to Local Authority District (February 2018) Lookup in the UK. https://geoportal.statistics.gov.uk/search?collection=Dataset&sort=name&tags=all(LUP_PCD_OA_LSOA_MSOA_LAD).

[CR88] R Core Team (2020). R: A language and environment for statistical computing.

[CR89] Rains, T. (2020). *Building a suite of store typologies in Retail.* Paper presented at the Spatial Data Science Conference, On-line.

[CR90] National Records of Scotland. (2020a). Scotland's Census. https://www.scotlandscensus.gov.uk/variables-classification/geography.

[CR91] National Records of Scotland. (2020b). Small Area Population Estimates (2011 Data Zone based). https://www.nrscotland.gov.uk/statistics-and-data/statistics/statistics-by-theme/population/population-estimates/2011-based-special-area-population-estimates/small-area-population-estimates.

[CR92] Ridgeway, G., McCaffrey, D., Morral, A., Burgette, L., & Griffin, B. A. (2017). *Toolkit for weighting and analysis of nonequivalent groups: A tutorial for the twang package*. RAND Corporation.

[CR93] Rosen S (1974). Hedonic prices and implicit markets: Product differentiation in pure competition. Journal of Political Economy.

[CR94] Rosenbaum PR, Rubin DB (1983). The central role of the propensity score in observational studies for causal effects. Biometrika.

[CR95] Scottish Government/Riaghaltas na h-Alba. (2017). Small Area Income Estimates. https://www.gov.scot/publications/chma-small-area-income-estimates/.

[CR96] Scottish Government/Riaghaltas na h-Alba. (2020). Scottish Index of Multiple Deprivation 2020. https://www.gov.scot/collections/scottish-index-of-multiple-deprivation-2020/.

[CR97] Singla HK, Bendigiri P (2019). Factors affecting rentals of residential apartments in Pune, India: An empirical investigation. International Journal of Housing Markets and Analysis.

[CR98] Sirpal R (1994). Empirical modeling of the relative impacts of various sizes of shopping centers on the values of surrounding residential properties. Journal of Real Estate Research.

[CR99] Song Y, Sohn J (2007). Valuing spatial accessibility to retailing: A case study of the single family housing market in Hillsboro, Oregon. Journal of Retailing and Consumer Services.

[CR100] Stadelmann D (2010). Which factors capitalize into house prices? A Bayesian averaging approach. Journal of Housing Economics.

[CR101] Thompson C, Clarke G, Clarke M, Stillwell J (2012). Modelling the future opportunities for deep discount food retailing in the UK. The International Review of Retail, Distribution and Consumer Research.

[CR102] UK Data Service. (2017). Acorn Postcode-Level Directory for the United Kingdom, 2017. https://beta.ukdataservice.ac.uk/datacatalogue/studies/study?id=8196.

[CR103] Urban Big Data Centre. (2020). Zoopla Property Data. https://www.ubdc.ac.uk/data-services/data-catalogue/housing-data/zoopla-property-data/.

[CR104] Waddell P, Berry BJ, Hoch I (1993). Housing price gradients: The intersection of space and built form. Geographical Analysis.

[CR105] Wilcox S, Perry J, Stephens M, Williams P (2017). United Kingdon housing review 2017 briefing paper.

[CR106] Wrigley N, Guy C, Lowe M (2002). Urban regeneration, social inclusion and large store development: The Seacroft development in context. Urban Studies.

[CR107] Xiao Y, Orford S, Webster CJ (2016). Urban configuration, accessibility, and property prices: A case study of Cardiff, Wales. Environment and Planning B: Planning and Design.

[CR108] Zentes, J., Morschett, D., & Schramm-Klein, H. (2017). *Retail Branding and Positioning* (Strategic Retail Management): Springer.

[CR109] Zheng S, Xu Y, Zhang X, Wang R (2016). Transit development, consumer amenities and home values: Evidence from Beijing's subway neighborhoods. Journal of Housing Economics.

[CR110] Zoopla. (2020). We know what a home is really worth. https://www.zoopla.co.uk/.

