# Supplementary material for: Identifying the effect of retail brands on private residential rental prices in Great Britain
Source: J Hous Built Environ. 2021 Oct 5;37(3):1489–509. doi: 10.1007/s10901-021-09904-2 (PMC8491747; doi:10.1007/s10901-021-09904-2)
Supplement: Supplementary file 1 — Supplementary file1 (DOCX 52 KB) [file 10901_2021_9904_MOESM1_ESM.docx]

[1] "Table S1 : Discounter"

$unw

tx.mn tx.sd ct.mn ct.sd std.eff.sz stat p ks ks.pval

freezer.count 0.705 0.456 0.296 0.457 0.896 32.375 0.000 0.409 0.696

Budgens.Spar.count 0.233 0.423 0.187 0.390 0.108 4.056 0.000 0.046 0.700

Co.op.count 0.535 0.499 0.492 0.500 0.086 3.108 0.002 0.043 0.703

Asda.count 0.765 0.424 0.523 0.500 0.571 18.883 0.000 0.242 0.696

Morrisons.count 0.680 0.467 0.449 0.497 0.495 17.299 0.000 0.231 0.696

Sainsbury.count 0.790 0.408 0.609 0.488 0.443 14.518 0.000 0.181 0.696

Tesco.count 0.881 0.324 0.680 0.467 0.622 18.139 0.000 0.201 0.696

premium.count 0.553 0.497 0.411 0.492 0.285 10.340 0.000 0.142 0.696

Detached 7.685 6.297 14.623 11.702 -1.102 -26.746 0.000 0.306 0.696

SemiDetached 20.126 13.599 24.553 12.871 -0.326 -12.078 0.000 0.189 0.696

Terraced 28.227 17.317 23.917 14.071 0.249 9.861 0.000 0.141 0.696

Flats 43.962 24.714 36.907 24.148 0.285 10.432 0.000 0.154 0.696

Rooms1 2.092 2.452 1.623 2.466 0.191 6.891 0.000 0.168 0.696

Rooms23 25.404 12.279 21.296 11.315 0.335 12.564 0.000 0.168 0.696

Rooms45 50.758 10.375 51.271 9.116 -0.049 -1.895 0.058 0.052 0.697

Rooms6p 21.746 9.506 25.810 10.676 -0.428 -14.534 0.000 0.161 0.696

Affluent.Achievers 9.604 13.594 21.639 19.498 -0.885 -25.914 0.000 0.315 0.696

Rising.Prosperity 6.860 11.920 7.598 11.758 -0.062 -2.252 0.024 0.113 0.696

Comfortable.Communities 15.257 13.796 19.184 16.159 -0.285 -9.450 0.000 0.103 0.696

Financially.Stretched 15.494 12.659 12.292 12.185 0.253 9.309 0.000 0.163 0.696

Urban.Adversity 16.148 15.060 8.105 12.026 0.534 21.301 0.000 0.326 0.696

Not.Private.Households 7.401 6.069 5.518 5.287 0.310 11.945 0.000 0.183 0.696

Not.Found 29.237 18.503 25.664 17.751 0.193 7.118 0.000 0.112 0.696

ewLondonkm 4.042 1.379 3.827 1.452 0.156 5.483 0.000 0.116 0.696

nsLondonkm 4.019 1.709 3.813 1.656 0.121 4.425 0.000 0.105 0.696

ahah.v2 22.340 6.454 23.031 7.073 -0.107 -3.690 0.000 0.042 0.706

Lemployment 0.932 1.048 1.325 1.061 -0.375 -13.451 0.000 0.194 0.696

raildist 0.147 0.908 0.536 1.082 -0.429 -14.085 0.000 0.202 0.696

$es.mean.ATT

tx.mn tx.sd ct.mn ct.sd std.eff.sz stat p ks ks.pval

freezer.count 0.705 0.456 0.682 0.466 0.051 1.547 0.122 0.023 0.826

Budgens.Spar.count 0.233 0.423 0.237 0.425 -0.008 -0.203 0.839 0.003 1.000

Co.op.count 0.535 0.499 0.501 0.500 0.069 1.832 0.067 0.034 0.362

Asda.count 0.765 0.424 0.759 0.428 0.014 0.412 0.680 0.006 1.000

Morrisons.count 0.680 0.467 0.660 0.474 0.042 1.155 0.248 0.019 0.947

Sainsbury.count 0.790 0.408 0.789 0.408 0.001 0.038 0.970 0.001 1.000

Tesco.count 0.881 0.324 0.878 0.327 0.009 0.287 0.774 0.003 1.000

premium.count 0.553 0.497 0.539 0.499 0.026 0.710 0.478 0.013 1.000

Detached 7.685 6.297 7.935 6.566 -0.040 -1.283 0.199 0.036 0.296

SemiDetached 20.126 13.599 21.102 13.499 -0.072 -1.955 0.051 0.045 0.109

Terraced 28.227 17.317 27.721 16.152 0.029 0.770 0.441 0.038 0.249

Flats 43.962 24.714 43.242 23.783 0.029 0.792 0.428 0.032 0.465

Rooms1 2.092 2.452 2.052 2.432 0.016 0.465 0.642 0.024 0.789

Rooms23 25.404 12.279 25.175 12.216 0.019 0.455 0.649 0.032 0.455

Rooms45 50.758 10.375 50.848 10.249 -0.009 -0.220 0.826 0.033 0.427

Rooms6p 21.746 9.506 21.925 9.350 -0.019 -0.495 0.621 0.023 0.834

Affluent.Achievers 9.604 13.594 10.249 14.207 -0.047 -1.475 0.140 0.030 0.527

Rising.Prosperity 6.860 11.920 6.657 11.305 0.017 0.543 0.587 0.034 0.372

Comfortable.Communities 15.257 13.796 15.688 13.969 -0.031 -0.852 0.394 0.024 0.785

Financially.Stretched 15.494 12.659 15.048 12.298 0.035 1.023 0.306 0.026 0.711

Urban.Adversity 16.148 15.060 15.559 14.800 0.039 1.057 0.290 0.036 0.314

Not.Private.Households 7.401 6.069 7.279 6.313 0.020 0.505 0.614 0.026 0.724

Not.Found 29.237 18.503 29.522 18.599 -0.015 -0.399 0.690 0.024 0.794

ewLondonkm 4.042 1.379 4.056 1.342 -0.010 -0.308 0.758 0.030 0.532

nsLondonkm 4.019 1.709 4.034 1.654 -0.009 -0.246 0.805 0.026 0.718

ahah.v2 22.340 6.454 22.148 6.290 0.030 0.860 0.390 0.029 0.577

Lemployment 0.932 1.048 0.923 1.024 0.009 0.224 0.823 0.024 0.814

raildist 0.147 0.908 0.182 0.885 -0.038 -1.144 0.253 0.037 0.281

$es.max.ATT

tx.mn tx.sd ct.mn ct.sd std.eff.sz stat p ks ks.pval

freezer.count 0.705 0.456 0.682 0.466 0.050 1.532 0.126 0.023 0.835

Budgens.Spar.count 0.233 0.423 0.237 0.425 -0.008 -0.205 0.838 0.003 1.000

Co.op.count 0.535 0.499 0.501 0.500 0.069 1.829 0.067 0.034 0.364

Asda.count 0.765 0.424 0.759 0.428 0.014 0.420 0.674 0.006 1.000

Morrisons.count 0.680 0.467 0.660 0.474 0.042 1.162 0.245 0.019 0.944

Sainsbury.count 0.790 0.408 0.789 0.408 0.001 0.020 0.984 0.000 1.000

Tesco.count 0.881 0.324 0.878 0.327 0.010 0.323 0.747 0.003 1.000

premium.count 0.553 0.497 0.540 0.498 0.025 0.683 0.494 0.013 1.000

Detached 7.685 6.297 7.920 6.572 -0.037 -1.202 0.230 0.034 0.362

SemiDetached 20.126 13.599 21.068 13.518 -0.069 -1.882 0.060 0.044 0.130

Terraced 28.227 17.317 27.693 16.171 0.031 0.811 0.417 0.038 0.246

Flats 43.962 24.714 43.319 23.836 0.026 0.703 0.482 0.030 0.522

Rooms1 2.092 2.452 2.055 2.438 0.015 0.425 0.671 0.024 0.792

Rooms23 25.404 12.279 25.210 12.254 0.016 0.384 0.701 0.031 0.472

Rooms45 50.758 10.375 50.848 10.275 -0.009 -0.220 0.826 0.033 0.401

Rooms6p 21.746 9.506 21.887 9.366 -0.015 -0.388 0.698 0.022 0.861

Affluent.Achievers 9.604 13.594 10.262 14.222 -0.048 -1.505 0.132 0.031 0.505

Rising.Prosperity 6.860 11.920 6.657 11.293 0.017 0.544 0.587 0.035 0.331

Comfortable.Communities 15.257 13.796 15.651 13.964 -0.029 -0.780 0.435 0.023 0.840

Financially.Stretched 15.494 12.659 15.045 12.318 0.035 1.032 0.302 0.027 0.679

Urban.Adversity 16.148 15.060 15.532 14.806 0.041 1.107 0.268 0.037 0.276

Not.Private.Households 7.401 6.069 7.287 6.330 0.019 0.468 0.640 0.025 0.737

Not.Found 29.237 18.503 29.567 18.620 -0.018 -0.462 0.644 0.025 0.742

ewLondonkm 4.042 1.379 4.059 1.344 -0.012 -0.365 0.715 0.029 0.595

nsLondonkm 4.019 1.709 4.037 1.655 -0.011 -0.309 0.757 0.024 0.794

ahah.v2 22.340 6.454 22.128 6.288 0.033 0.950 0.342 0.030 0.530

Lemployment 0.932 1.048 0.923 1.027 0.009 0.229 0.819 0.024 0.787

raildist 0.147 0.908 0.182 0.884 -0.038 -1.147 0.251 0.037 0.275

$ks.mean.ATT

tx.mn tx.sd ct.mn ct.sd std.eff.sz stat p ks ks.pval

freezer.count 0.705 0.456 0.685 0.465 0.043 1.338 0.181 0.020 0.932

Budgens.Spar.count 0.233 0.423 0.237 0.425 -0.009 -0.242 0.809 0.004 1.000

Co.op.count 0.535 0.499 0.495 0.500 0.081 2.162 0.031 0.040 0.188

Asda.count 0.765 0.424 0.760 0.427 0.012 0.360 0.719 0.005 1.000

Morrisons.count 0.680 0.467 0.662 0.473 0.038 1.082 0.279 0.018 0.970

Sainsbury.count 0.790 0.408 0.787 0.409 0.006 0.158 0.874 0.002 1.000

Tesco.count 0.881 0.324 0.876 0.329 0.015 0.486 0.627 0.005 1.000

premium.count 0.553 0.497 0.536 0.499 0.034 0.912 0.362 0.017 0.985

Detached 7.685 6.297 7.863 6.608 -0.028 -0.916 0.360 0.028 0.606

SemiDetached 20.126 13.599 20.978 13.574 -0.063 -1.702 0.089 0.038 0.227

Terraced 28.227 17.317 27.701 16.198 0.030 0.808 0.419 0.039 0.217

Flats 43.962 24.714 43.458 23.940 0.020 0.552 0.581 0.027 0.658

Rooms1 2.092 2.452 2.054 2.455 0.015 0.437 0.662 0.026 0.692

Rooms23 25.404 12.279 25.267 12.293 0.011 0.272 0.786 0.031 0.483

Rooms45 50.758 10.375 50.872 10.308 -0.011 -0.281 0.779 0.035 0.325

Rooms6p 21.746 9.506 21.807 9.452 -0.006 -0.166 0.868 0.027 0.639

Affluent.Achievers 9.604 13.594 10.259 14.274 -0.048 -1.507 0.132 0.030 0.542

Rising.Prosperity 6.860 11.920 6.653 11.241 0.017 0.562 0.574 0.033 0.392

Comfortable.Communities 15.257 13.796 15.461 13.955 -0.015 -0.410 0.682 0.016 0.989

Financially.Stretched 15.494 12.659 15.156 12.493 0.027 0.769 0.442 0.025 0.751

Urban.Adversity 16.148 15.060 15.585 14.966 0.037 1.004 0.315 0.036 0.300

Not.Private.Households 7.401 6.069 7.309 6.424 0.015 0.373 0.709 0.026 0.718

Not.Found 29.237 18.503 29.576 18.637 -0.018 -0.478 0.633 0.026 0.692

ewLondonkm 4.042 1.379 4.067 1.353 -0.018 -0.549 0.583 0.025 0.747

nsLondonkm 4.019 1.709 4.053 1.661 -0.020 -0.573 0.567 0.025 0.754

ahah.v2 22.340 6.454 22.039 6.292 0.047 1.363 0.173 0.034 0.357

Lemployment 0.932 1.048 0.908 1.035 0.023 0.579 0.563 0.023 0.836

raildist 0.147 0.908 0.181 0.884 -0.037 -1.099 0.272 0.039 0.221

$ks.max.ATT

tx.mn tx.sd ct.mn ct.sd std.eff.sz stat p ks ks.pval

freezer.count 0.705 0.456 0.684 0.465 0.045 1.394 0.164 0.021 0.908

Budgens.Spar.count 0.233 0.423 0.237 0.425 -0.009 -0.231 0.817 0.004 1.000

Co.op.count 0.535 0.499 0.496 0.500 0.079 2.124 0.034 0.039 0.204

Asda.count 0.765 0.424 0.760 0.427 0.013 0.398 0.691 0.006 1.000

Morrisons.count 0.680 0.467 0.661 0.473 0.040 1.116 0.264 0.019 0.960

Sainsbury.count 0.790 0.408 0.787 0.409 0.006 0.160 0.873 0.002 1.000

Tesco.count 0.881 0.324 0.876 0.330 0.016 0.500 0.617 0.005 1.000

premium.count 0.553 0.497 0.536 0.499 0.033 0.903 0.366 0.017 0.986

Detached 7.685 6.297 7.881 6.617 -0.031 -1.011 0.312 0.029 0.554

SemiDetached 20.126 13.599 20.999 13.562 -0.064 -1.749 0.080 0.039 0.202

Terraced 28.227 17.317 27.673 16.170 0.032 0.854 0.393 0.040 0.202

Flats 43.962 24.714 43.447 23.910 0.021 0.568 0.570 0.027 0.638

Rooms1 2.092 2.452 2.056 2.455 0.015 0.416 0.678 0.025 0.724

Rooms23 25.404 12.279 25.258 12.272 0.012 0.292 0.770 0.031 0.495

Rooms45 50.758 10.375 50.857 10.293 -0.010 -0.245 0.807 0.035 0.335

Rooms6p 21.746 9.506 21.829 9.437 -0.009 -0.228 0.819 0.026 0.705

Affluent.Achievers 9.604 13.594 10.287 14.284 -0.050 -1.571 0.116 0.031 0.493

Rising.Prosperity 6.860 11.920 6.668 11.266 0.016 0.520 0.603 0.034 0.360

Comfortable.Communities 15.257 13.796 15.506 13.968 -0.018 -0.502 0.616 0.017 0.977

Financially.Stretched 15.494 12.659 15.132 12.468 0.029 0.826 0.409 0.025 0.735

Urban.Adversity 16.148 15.060 15.557 14.938 0.039 1.058 0.290 0.036 0.285

Not.Private.Households 7.401 6.069 7.291 6.403 0.018 0.447 0.655 0.026 0.680

Not.Found 29.237 18.503 29.558 18.638 -0.017 -0.453 0.651 0.026 0.716

ewLondonkm 4.042 1.379 4.064 1.353 -0.016 -0.479 0.632 0.026 0.686

nsLondonkm 4.019 1.709 4.049 1.660 -0.018 -0.512 0.609 0.024 0.806

ahah.v2 22.340 6.454 22.055 6.293 0.044 1.292 0.197 0.033 0.391

Lemployment 0.932 1.048 0.912 1.033 0.020 0.497 0.619 0.022 0.847

raildist 0.147 0.908 0.180 0.884 -0.036 -1.094 0.274 0.038 0.234

[1] "Table S2 : Freezer"

$unw

tx.mn tx.sd ct.mn ct.sd std.eff.sz stat p ks ks.pval

discount.count 0.700 0.458 0.291 0.455 0.892 32.374 0.000 0.409 0.755

Budgens.Spar.count 0.239 0.427 0.181 0.385 0.137 5.180 0.000 0.058 0.755

Co.op.count 0.523 0.500 0.504 0.500 0.037 1.349 0.178 0.019 0.975

Asda.count 0.788 0.409 0.499 0.500 0.706 22.844 0.000 0.289 0.755

Morrisons.count 0.706 0.455 0.421 0.494 0.627 21.723 0.000 0.286 0.755

Sainsbury.count 0.816 0.388 0.582 0.493 0.604 19.084 0.000 0.234 0.755

Tesco.count 0.894 0.308 0.667 0.472 0.736 20.602 0.000 0.227 0.755

premium.count 0.581 0.493 0.382 0.486 0.405 14.732 0.000 0.200 0.755

Detached 6.794 5.646 15.553 11.448 -1.552 -35.098 0.000 0.389 0.755

SemiDetached 18.827 13.722 25.873 12.133 -0.513 -19.652 0.000 0.285 0.755

Terraced 27.726 17.687 24.388 13.717 0.189 7.617 0.000 0.131 0.755

Flats 46.653 25.586 34.186 22.060 0.487 18.854 0.000 0.213 0.755

Rooms1 2.446 2.823 1.267 1.884 0.418 17.740 0.000 0.257 0.755

Rooms23 26.567 12.673 20.112 10.283 0.509 20.204 0.000 0.228 0.755

Rooms45 50.142 10.783 51.887 8.543 -0.162 -6.480 0.000 0.133 0.755

Rooms6p 20.844 9.410 26.733 10.338 -0.626 -21.528 0.000 0.242 0.755

Affluent.Achievers 8.315 12.608 22.998 19.283 -1.165 -32.584 0.000 0.405 0.755

Rising.Prosperity 7.928 13.193 6.540 10.283 0.105 4.239 0.000 0.102 0.755

Comfortable.Communities 13.983 13.678 20.476 15.858 -0.475 -15.847 0.000 0.202 0.755

Financially.Stretched 15.121 12.934 12.642 11.978 0.192 7.185 0.000 0.119 0.755

Urban.Adversity 17.176 15.513 7.030 10.547 0.654 27.622 0.000 0.388 0.755

Not.Private.Households 7.578 6.319 5.330 4.905 0.356 14.353 0.000 0.189 0.755

Not.Found 29.898 18.497 24.983 17.592 0.266 9.839 0.000 0.158 0.755

ewLondonkm 3.877 1.481 3.990 1.356 -0.076 -2.875 0.004 0.085 0.755

nsLondonkm 3.904 1.840 3.925 1.517 -0.012 -0.454 0.650 0.124 0.755

ahah.v2 23.288 7.074 22.093 6.425 0.169 6.393 0.000 0.078 0.755

Lemployment 0.837 1.042 1.422 1.022 -0.561 -20.463 0.000 0.261 0.755

raildist 0.065 0.876 0.621 1.073 -0.634 -20.485 0.000 0.277 0.755

$es.mean.ATT

tx.mn tx.sd ct.mn ct.sd std.eff.sz stat p ks ks.pval

discount.count 0.700 0.458 0.678 0.467 0.050 1.247 0.213 0.023 0.949

Budgens.Spar.count 0.239 0.427 0.230 0.421 0.022 0.494 0.621 0.009 1.000

Co.op.count 0.523 0.500 0.520 0.500 0.006 0.139 0.890 0.003 1.000

Asda.count 0.788 0.409 0.775 0.418 0.032 0.797 0.426 0.013 1.000

Morrisons.count 0.706 0.455 0.693 0.462 0.031 0.757 0.449 0.014 1.000

Sainsbury.count 0.816 0.388 0.818 0.386 -0.005 -0.133 0.894 0.002 1.000

Tesco.count 0.894 0.308 0.889 0.314 0.014 0.413 0.680 0.004 1.000

premium.count 0.581 0.493 0.574 0.495 0.014 0.325 0.745 0.007 1.000

Detached 6.794 5.646 7.359 5.990 -0.100 -2.888 0.004 0.087 0.001

SemiDetached 18.827 13.722 20.574 13.604 -0.127 -2.996 0.003 0.084 0.002

Terraced 27.726 17.687 27.163 16.866 0.032 0.692 0.489 0.029 0.757

Flats 46.653 25.586 44.903 25.180 0.068 1.493 0.136 0.047 0.199

Rooms1 2.446 2.823 2.203 2.619 0.086 1.777 0.076 0.048 0.192

Rooms23 26.567 12.673 25.789 12.256 0.061 1.309 0.191 0.035 0.564

Rooms45 50.142 10.783 51.027 10.251 -0.082 -1.797 0.072 0.050 0.145

Rooms6p 20.844 9.410 20.981 9.438 -0.015 -0.326 0.745 0.023 0.943

Affluent.Achievers 8.315 12.608 9.286 13.395 -0.077 -2.060 0.039 0.036 0.494

Rising.Prosperity 7.928 13.193 7.589 12.759 0.026 0.579 0.563 0.022 0.967

Comfortable.Communities 13.983 13.678 14.943 14.197 -0.070 -1.574 0.116 0.037 0.478

Financially.Stretched 15.121 12.934 15.779 13.232 -0.051 -1.155 0.248 0.033 0.606

Urban.Adversity 17.176 15.513 15.989 14.763 0.077 1.677 0.094 0.049 0.163

Not.Private.Households 7.578 6.319 7.252 6.372 0.052 1.069 0.285 0.037 0.484

Not.Found 29.898 18.497 29.162 18.497 0.040 0.870 0.384 0.041 0.358

ewLondonkm 3.877 1.481 3.906 1.406 -0.020 -0.491 0.623 0.049 0.171

nsLondonkm 3.904 1.840 3.915 1.781 -0.006 -0.129 0.897 0.033 0.639

ahah.v2 23.288 7.074 22.548 6.257 0.105 2.554 0.011 0.065 0.026

Lemployment 0.837 1.042 0.824 0.975 0.012 0.284 0.777 0.023 0.951

raildist 0.065 0.876 0.146 0.870 -0.093 -2.310 0.021 0.061 0.043

$es.max.ATT

tx.mn tx.sd ct.mn ct.sd std.eff.sz stat p ks ks.pval

discount.count 0.700 0.458 0.679 0.467 0.047 1.158 0.247 0.021 0.974

Budgens.Spar.count 0.239 0.427 0.230 0.421 0.023 0.504 0.614 0.010 1.000

Co.op.count 0.523 0.500 0.515 0.500 0.015 0.333 0.739 0.008 1.000

Asda.count 0.788 0.409 0.775 0.418 0.032 0.799 0.424 0.013 1.000

Morrisons.count 0.706 0.455 0.694 0.461 0.027 0.650 0.516 0.012 1.000

Sainsbury.count 0.816 0.388 0.820 0.385 -0.009 -0.237 0.813 0.004 1.000

Tesco.count 0.894 0.308 0.889 0.315 0.016 0.469 0.639 0.005 1.000

premium.count 0.581 0.493 0.576 0.494 0.012 0.264 0.792 0.006 1.000

Detached 6.794 5.646 7.392 5.947 -0.106 -2.992 0.003 0.089 0.001

SemiDetached 18.827 13.722 20.556 13.481 -0.126 -2.946 0.003 0.086 0.001

Terraced 27.726 17.687 27.336 16.885 0.022 0.468 0.639 0.027 0.861

Flats 46.653 25.586 44.717 25.058 0.076 1.615 0.106 0.049 0.176

Rooms1 2.446 2.823 2.195 2.599 0.089 1.800 0.072 0.047 0.225

Rooms23 26.567 12.673 25.697 12.233 0.069 1.416 0.157 0.039 0.446

Rooms45 50.142 10.783 51.043 10.249 -0.084 -1.788 0.074 0.051 0.153

Rooms6p 20.844 9.410 21.066 9.410 -0.024 -0.513 0.608 0.026 0.874

Affluent.Achievers 8.315 12.608 9.405 13.421 -0.086 -2.251 0.024 0.042 0.327

Rising.Prosperity 7.928 13.193 7.486 12.655 0.033 0.740 0.459 0.024 0.931

Comfortable.Communities 13.983 13.678 15.014 14.148 -0.075 -1.666 0.096 0.041 0.371

Financially.Stretched 15.121 12.934 15.703 13.180 -0.045 -1.018 0.309 0.032 0.686

Urban.Adversity 17.176 15.513 16.001 14.752 0.076 1.619 0.105 0.048 0.199

Not.Private.Households 7.578 6.319 7.271 6.317 0.049 1.013 0.311 0.035 0.553

Not.Found 29.898 18.497 29.120 18.464 0.042 0.901 0.368 0.042 0.353

ewLondonkm 3.877 1.481 3.914 1.394 -0.025 -0.630 0.528 0.049 0.178

nsLondonkm 3.904 1.840 3.925 1.770 -0.011 -0.246 0.805 0.033 0.644

ahah.v2 23.288 7.074 22.539 6.228 0.106 2.519 0.012 0.065 0.030

Lemployment 0.837 1.042 0.834 0.976 0.003 0.071 0.944 0.026 0.879

raildist 0.065 0.876 0.146 0.868 -0.092 -2.217 0.027 0.060 0.054

$ks.mean.ATT

tx.mn tx.sd ct.mn ct.sd std.eff.sz stat p ks ks.pval

discount.count 0.700 0.458 0.678 0.467 0.050 1.248 0.212 0.023 0.949

Budgens.Spar.count 0.239 0.427 0.230 0.421 0.022 0.494 0.621 0.009 1.000

Co.op.count 0.523 0.500 0.520 0.500 0.006 0.140 0.889 0.003 1.000

Asda.count 0.788 0.409 0.775 0.418 0.032 0.797 0.426 0.013 1.000

Morrisons.count 0.706 0.455 0.693 0.462 0.031 0.755 0.450 0.014 1.000

Sainsbury.count 0.816 0.388 0.818 0.386 -0.005 -0.132 0.895 0.002 1.000

Tesco.count 0.894 0.308 0.889 0.314 0.014 0.413 0.680 0.004 1.000

premium.count 0.581 0.493 0.574 0.495 0.014 0.326 0.744 0.007 1.000

Detached 6.794 5.646 7.359 5.990 -0.100 -2.885 0.004 0.087 0.001

SemiDetached 18.827 13.722 20.576 13.605 -0.127 -2.999 0.003 0.084 0.001

Terraced 27.726 17.687 27.160 16.862 0.032 0.697 0.486 0.030 0.751

Flats 46.653 25.586 44.905 25.179 0.068 1.491 0.136 0.047 0.199

Rooms1 2.446 2.823 2.203 2.619 0.086 1.778 0.076 0.048 0.191

Rooms23 26.567 12.673 25.789 12.256 0.061 1.308 0.191 0.035 0.564

Rooms45 50.142 10.783 51.028 10.251 -0.082 -1.799 0.072 0.050 0.144

Rooms6p 20.844 9.410 20.980 9.438 -0.014 -0.323 0.747 0.023 0.944

Affluent.Achievers 8.315 12.608 9.285 13.395 -0.077 -2.059 0.040 0.036 0.495

Rising.Prosperity 7.928 13.193 7.589 12.758 0.026 0.580 0.562 0.022 0.966

Comfortable.Communities 13.983 13.678 14.943 14.197 -0.070 -1.573 0.116 0.037 0.478

Financially.Stretched 15.121 12.934 15.780 13.232 -0.051 -1.157 0.247 0.033 0.605

Urban.Adversity 17.176 15.513 15.990 14.764 0.076 1.676 0.094 0.049 0.163

Not.Private.Households 7.578 6.319 7.253 6.374 0.051 1.067 0.286 0.037 0.483

Not.Found 29.898 18.497 29.161 18.499 0.040 0.871 0.384 0.041 0.356

ewLondonkm 3.877 1.481 3.906 1.406 -0.020 -0.494 0.621 0.049 0.172

nsLondonkm 3.904 1.840 3.915 1.781 -0.006 -0.131 0.895 0.032 0.643

ahah.v2 23.288 7.074 22.545 6.258 0.105 2.561 0.010 0.065 0.025

Lemployment 0.837 1.042 0.825 0.975 0.012 0.282 0.778 0.023 0.951

raildist 0.065 0.876 0.146 0.870 -0.093 -2.310 0.021 0.061 0.043

$ks.max.ATT

tx.mn tx.sd ct.mn ct.sd std.eff.sz stat p ks ks.pval

discount.count 0.700 0.458 0.666 0.472 0.074 1.964 0.050 0.034 0.494

Budgens.Spar.count 0.239 0.427 0.227 0.419 0.029 0.710 0.478 0.013 1.000

Co.op.count 0.523 0.500 0.543 0.498 -0.040 -0.978 0.328 0.020 0.965

Asda.count 0.788 0.409 0.767 0.423 0.051 1.346 0.178 0.021 0.957

Morrisons.count 0.706 0.455 0.678 0.467 0.062 1.600 0.110 0.028 0.724

Sainsbury.count 0.816 0.388 0.807 0.395 0.024 0.654 0.513 0.009 1.000

Tesco.count 0.894 0.308 0.883 0.321 0.033 0.985 0.324 0.010 1.000

premium.count 0.581 0.493 0.560 0.496 0.043 1.053 0.292 0.021 0.950

Detached 6.794 5.646 7.288 6.183 -0.088 -2.654 0.008 0.073 0.004

SemiDetached 18.827 13.722 20.560 13.854 -0.126 -3.080 0.002 0.078 0.001

Terraced 27.726 17.687 26.627 16.779 0.062 1.475 0.140 0.038 0.347

Flats 46.653 25.586 45.525 25.559 0.044 1.031 0.303 0.037 0.384

Rooms1 2.446 2.823 2.178 2.619 0.095 2.198 0.028 0.054 0.060

Rooms23 26.567 12.673 26.124 12.515 0.035 0.808 0.419 0.030 0.664

Rooms45 50.142 10.783 51.016 10.307 -0.081 -1.938 0.053 0.056 0.045

Rooms6p 20.844 9.410 20.681 9.709 0.017 0.411 0.681 0.034 0.477

Affluent.Achievers 8.315 12.608 9.186 13.426 -0.069 -2.002 0.045 0.037 0.392

Rising.Prosperity 7.928 13.193 8.129 13.453 -0.015 -0.357 0.721 0.032 0.574

Comfortable.Communities 13.983 13.678 14.863 14.204 -0.064 -1.553 0.121 0.036 0.408

Financially.Stretched 15.121 12.934 16.069 13.499 -0.073 -1.724 0.085 0.041 0.269

Urban.Adversity 17.176 15.513 16.051 15.098 0.073 1.646 0.100 0.051 0.090

Not.Private.Households 7.578 6.319 7.102 6.463 0.075 1.578 0.115 0.047 0.138

Not.Found 29.898 18.497 28.600 18.446 0.070 1.688 0.092 0.053 0.070

ewLondonkm 3.877 1.481 3.842 1.483 0.023 0.586 0.558 0.049 0.110

nsLondonkm 3.904 1.840 3.849 1.801 0.030 0.712 0.477 0.038 0.344

ahah.v2 23.288 7.074 22.501 6.538 0.111 2.772 0.006 0.064 0.014

Lemployment 0.837 1.042 0.827 0.984 0.010 0.251 0.802 0.029 0.674

raildist 0.065 0.876 0.136 0.893 -0.081 -2.126 0.034 0.060 0.030

[1] "Table S4 : Co-op"

$unw

tx.mn tx.sd ct.mn ct.sd std.eff.sz stat p ks ks.pval

discount.count 0.516 0.500 0.473 0.499 0.086 3.108 0.002 0.043 0.571

freezer.count 0.508 0.500 0.489 0.500 0.037 1.349 0.178 0.019 0.904

Budgens.Spar.count 0.222 0.416 0.198 0.398 0.058 2.157 0.031 0.024 0.756

Asda.count 0.659 0.474 0.627 0.484 0.067 2.408 0.016 0.032 0.629

Morrisons.count 0.576 0.494 0.550 0.498 0.052 1.886 0.059 0.026 0.722

Sainsbury.count 0.743 0.437 0.652 0.476 0.207 7.153 0.000 0.091 0.563

Tesco.count 0.804 0.397 0.754 0.431 0.127 4.376 0.000 0.050 0.565

premium.count 0.533 0.499 0.427 0.495 0.212 7.696 0.000 0.106 0.563

Detached 9.355 8.588 13.119 11.048 -0.438 -13.697 0.000 0.168 0.563

SemiDetached 20.629 14.026 24.187 12.492 -0.254 -9.692 0.000 0.144 0.563

Terraced 24.433 15.749 27.761 15.898 -0.211 -7.597 0.000 0.108 0.563

Flats 45.584 26.460 34.932 21.332 0.403 16.054 0.000 0.157 0.563

Rooms1 2.186 2.831 1.506 1.960 0.240 10.144 0.000 0.105 0.563

Rooms23 25.363 12.800 21.185 10.634 0.326 12.858 0.000 0.139 0.563

Rooms45 49.946 10.282 52.148 9.047 -0.214 -8.225 0.000 0.099 0.563

Rooms6p 22.504 10.334 25.162 10.119 -0.257 -9.389 0.000 0.112 0.563

Affluent.Achievers 14.399 16.702 17.028 18.947 -0.157 -5.309 0.000 0.062 0.563

Rising.Prosperity 9.130 13.998 5.229 8.586 0.279 12.209 0.000 0.117 0.563

Comfortable.Communities 16.569 14.478 17.945 15.823 -0.095 -3.273 0.001 0.091 0.563

Financially.Stretched 13.914 12.671 13.840 12.369 0.006 0.211 0.833 0.026 0.711

Urban.Adversity 12.180 14.073 11.992 14.322 0.013 0.479 0.632 0.084 0.563

Not.Private.Households 6.352 5.888 6.555 5.631 -0.035 -1.276 0.202 0.042 0.574

Not.Found 27.456 18.430 27.410 17.985 0.003 0.092 0.927 0.022 0.810

ewLondonkm 3.867 1.510 4.004 1.316 -0.091 -3.499 0.000 0.066 0.563

nsLondonkm 3.814 1.771 4.022 1.584 -0.118 -4.484 0.000 0.079 0.563

ahah.v2 22.755 7.229 22.619 6.276 0.019 0.726 0.468 0.039 0.582

Lemployment 0.976 1.076 1.293 1.045 -0.295 -10.818 0.000 0.131 0.563

raildist 0.258 1.010 0.435 1.019 -0.175 -6.306 0.000 0.080 0.563

$es.mean.ATT

tx.mn tx.sd ct.mn ct.sd std.eff.sz stat p ks ks.pval

discount.count 0.516 0.500 0.483 0.500 0.066 2.070 0.038 0.033 0.229

freezer.count 0.508 0.500 0.505 0.500 0.005 0.148 0.882 0.002 1.000

Budgens.Spar.count 0.222 0.416 0.221 0.415 0.001 0.043 0.966 0.001 1.000

Asda.count 0.659 0.474 0.674 0.469 -0.033 -1.063 0.288 0.016 0.967

Morrisons.count 0.576 0.494 0.592 0.492 -0.033 -1.037 0.300 0.016 0.958

Sainsbury.count 0.743 0.437 0.742 0.438 0.003 0.086 0.932 0.001 1.000

Tesco.count 0.804 0.397 0.814 0.389 -0.024 -0.835 0.404 0.010 1.000

premium.count 0.533 0.499 0.512 0.500 0.041 1.282 0.200 0.020 0.801

Detached 9.355 8.588 10.126 8.839 -0.090 -2.920 0.004 0.060 0.002

SemiDetached 20.629 14.026 21.749 13.689 -0.080 -2.328 0.020 0.049 0.019

Terraced 24.433 15.749 25.079 15.558 -0.041 -1.244 0.214 0.033 0.222

Flats 45.584 26.460 43.046 25.584 0.096 2.558 0.011 0.047 0.026

Rooms1 2.186 2.831 1.995 2.711 0.068 1.544 0.123 0.047 0.028

Rooms23 25.363 12.800 24.622 12.423 0.058 1.558 0.119 0.033 0.227

Rooms45 49.946 10.282 50.900 9.538 -0.093 -2.806 0.005 0.054 0.006

Rooms6p 22.504 10.334 22.483 10.139 0.002 0.060 0.952 0.025 0.572

Affluent.Achievers 14.399 16.702 15.292 17.166 -0.053 -1.735 0.083 0.035 0.179

Rising.Prosperity 9.130 13.998 8.067 12.372 0.076 1.928 0.054 0.030 0.329

Comfortable.Communities 16.569 14.478 16.946 14.649 -0.026 -0.803 0.422 0.041 0.069

Financially.Stretched 13.914 12.671 14.050 12.545 -0.011 -0.343 0.732 0.027 0.479

Urban.Adversity 12.180 14.073 12.147 13.692 0.002 0.075 0.940 0.019 0.867

Not.Private.Households 6.352 5.888 6.275 5.481 0.013 0.439 0.661 0.018 0.917

Not.Found 27.456 18.430 27.223 17.782 0.013 0.414 0.679 0.022 0.699

ewLondonkm 3.867 1.510 3.891 1.491 -0.016 -0.419 0.676 0.035 0.168

nsLondonkm 3.814 1.771 3.863 1.703 -0.028 -0.796 0.426 0.034 0.206

ahah.v2 22.755 7.229 22.120 6.493 0.088 2.501 0.012 0.043 0.048

Lemployment 0.976 1.076 1.065 1.037 -0.083 -2.570 0.010 0.043 0.051

raildist 0.258 1.010 0.301 0.985 -0.043 -1.375 0.169 0.038 0.113

$es.max.ATT

tx.mn tx.sd ct.mn ct.sd std.eff.sz stat p ks ks.pval

discount.count 0.516 0.500 0.483 0.500 0.066 2.069 0.039 0.033 0.230

freezer.count 0.508 0.500 0.505 0.500 0.005 0.150 0.881 0.002 1.000

Budgens.Spar.count 0.222 0.416 0.221 0.415 0.001 0.036 0.971 0.001 1.000

Asda.count 0.659 0.474 0.674 0.469 -0.033 -1.064 0.287 0.016 0.967

Morrisons.count 0.576 0.494 0.592 0.492 -0.033 -1.047 0.295 0.016 0.954

Sainsbury.count 0.743 0.437 0.742 0.438 0.002 0.083 0.934 0.001 1.000

Tesco.count 0.804 0.397 0.813 0.390 -0.023 -0.814 0.416 0.009 1.000

premium.count 0.533 0.499 0.512 0.500 0.041 1.274 0.203 0.020 0.806

Detached 9.355 8.588 10.119 8.833 -0.089 -2.896 0.004 0.060 0.002

SemiDetached 20.629 14.026 21.744 13.690 -0.079 -2.316 0.021 0.049 0.019

Terraced 24.433 15.749 25.077 15.556 -0.041 -1.240 0.215 0.033 0.221

Flats 45.584 26.460 43.059 25.587 0.095 2.543 0.011 0.047 0.027

Rooms1 2.186 2.831 1.995 2.711 0.068 1.543 0.123 0.047 0.028

Rooms23 25.363 12.800 24.624 12.421 0.058 1.553 0.120 0.033 0.233

Rooms45 49.946 10.282 50.902 9.538 -0.093 -2.810 0.005 0.054 0.006

Rooms6p 22.504 10.334 22.479 10.138 0.002 0.071 0.944 0.025 0.580

Affluent.Achievers 14.399 16.702 15.288 17.157 -0.053 -1.727 0.084 0.035 0.176

Rising.Prosperity 9.130 13.998 8.068 12.375 0.076 1.925 0.054 0.030 0.331

Comfortable.Communities 16.569 14.478 16.938 14.641 -0.025 -0.786 0.432 0.041 0.071

Financially.Stretched 13.914 12.671 14.049 12.543 -0.011 -0.339 0.735 0.027 0.479

Urban.Adversity 12.180 14.073 12.146 13.701 0.002 0.078 0.938 0.019 0.867

Not.Private.Households 6.352 5.888 6.277 5.482 0.013 0.431 0.667 0.018 0.912

Not.Found 27.456 18.430 27.235 17.795 0.012 0.391 0.696 0.022 0.721

ewLondonkm 3.867 1.510 3.891 1.491 -0.016 -0.429 0.668 0.035 0.169

nsLondonkm 3.814 1.771 3.864 1.703 -0.028 -0.817 0.414 0.034 0.203

ahah.v2 22.755 7.229 22.117 6.493 0.088 2.510 0.012 0.044 0.048

Lemployment 0.976 1.076 1.064 1.037 -0.082 -2.567 0.010 0.043 0.051

raildist 0.258 1.010 0.301 0.985 -0.043 -1.377 0.169 0.038 0.114

$ks.mean.ATT

tx.mn tx.sd ct.mn ct.sd std.eff.sz stat p ks ks.pval

discount.count 0.516 0.500 0.483 0.500 0.066 2.063 0.039 0.033 0.232

freezer.count 0.508 0.500 0.505 0.500 0.005 0.143 0.886 0.002 1.000

Budgens.Spar.count 0.222 0.416 0.221 0.415 0.001 0.032 0.974 0.000 1.000

Asda.count 0.659 0.474 0.675 0.469 -0.033 -1.076 0.282 0.016 0.964

Morrisons.count 0.576 0.494 0.592 0.491 -0.033 -1.060 0.289 0.016 0.950

Sainsbury.count 0.743 0.437 0.741 0.438 0.003 0.091 0.927 0.001 1.000

Tesco.count 0.804 0.397 0.813 0.390 -0.023 -0.793 0.428 0.009 1.000

premium.count 0.533 0.499 0.512 0.500 0.041 1.286 0.198 0.021 0.797

Detached 9.355 8.588 10.118 8.835 -0.089 -2.887 0.004 0.060 0.002

SemiDetached 20.629 14.026 21.744 13.698 -0.080 -2.313 0.021 0.048 0.020

Terraced 24.433 15.749 25.079 15.563 -0.041 -1.242 0.214 0.033 0.224

Flats 45.584 26.460 43.059 25.601 0.095 2.539 0.011 0.047 0.027

Rooms1 2.186 2.831 1.995 2.715 0.068 1.538 0.124 0.047 0.028

Rooms23 25.363 12.800 24.617 12.426 0.058 1.565 0.118 0.033 0.221

Rooms45 49.946 10.282 50.907 9.544 -0.093 -2.819 0.005 0.054 0.006

Rooms6p 22.504 10.334 22.481 10.141 0.002 0.065 0.948 0.025 0.579

Affluent.Achievers 14.399 16.702 15.290 17.161 -0.053 -1.729 0.084 0.035 0.180

Rising.Prosperity 9.130 13.998 8.073 12.393 0.075 1.909 0.056 0.030 0.337

Comfortable.Communities 16.569 14.478 16.933 14.638 -0.025 -0.775 0.438 0.041 0.075

Financially.Stretched 13.914 12.671 14.057 12.550 -0.011 -0.359 0.719 0.027 0.466

Urban.Adversity 12.180 14.073 12.155 13.710 0.002 0.057 0.955 0.019 0.868

Not.Private.Households 6.352 5.888 6.271 5.478 0.014 0.460 0.646 0.017 0.922

Not.Found 27.456 18.430 27.220 17.787 0.013 0.418 0.676 0.022 0.710

ewLondonkm 3.867 1.510 3.892 1.491 -0.017 -0.449 0.653 0.035 0.168

nsLondonkm 3.814 1.771 3.866 1.704 -0.030 -0.848 0.396 0.034 0.205

ahah.v2 22.755 7.229 22.120 6.501 0.088 2.489 0.013 0.043 0.052

Lemployment 0.976 1.076 1.064 1.037 -0.082 -2.563 0.010 0.043 0.051

raildist 0.258 1.010 0.301 0.986 -0.044 -1.389 0.165 0.038 0.109

$ks.max.ATT

tx.mn tx.sd ct.mn ct.sd std.eff.sz stat p ks ks.pval

discount.count 0.516 0.500 0.483 0.500 0.066 2.066 0.039 0.033 0.231

freezer.count 0.508 0.500 0.505 0.500 0.005 0.145 0.884 0.002 1.000

Budgens.Spar.count 0.222 0.416 0.221 0.415 0.001 0.033 0.974 0.000 1.000

Asda.count 0.659 0.474 0.674 0.469 -0.033 -1.067 0.286 0.016 0.966

Morrisons.count 0.576 0.494 0.592 0.492 -0.033 -1.043 0.297 0.016 0.955

Sainsbury.count 0.743 0.437 0.742 0.438 0.002 0.080 0.936 0.001 1.000

Tesco.count 0.804 0.397 0.814 0.389 -0.024 -0.846 0.398 0.010 1.000

premium.count 0.533 0.499 0.512 0.500 0.041 1.270 0.204 0.020 0.810

Detached 9.355 8.588 10.119 8.837 -0.089 -2.895 0.004 0.060 0.002

SemiDetached 20.629 14.026 21.742 13.690 -0.079 -2.314 0.021 0.049 0.019

Terraced 24.433 15.749 25.078 15.561 -0.041 -1.241 0.215 0.033 0.225

Flats 45.584 26.460 43.060 25.591 0.095 2.542 0.011 0.047 0.027

Rooms1 2.186 2.831 1.995 2.712 0.067 1.539 0.124 0.046 0.028

Rooms23 25.363 12.800 24.627 12.426 0.058 1.547 0.122 0.033 0.232

Rooms45 49.946 10.282 50.901 9.540 -0.093 -2.807 0.005 0.054 0.006

Rooms6p 22.504 10.334 22.476 10.139 0.003 0.078 0.938 0.025 0.585

Affluent.Achievers 14.399 16.702 15.279 17.155 -0.053 -1.711 0.087 0.035 0.182

Rising.Prosperity 9.130 13.998 8.069 12.374 0.076 1.924 0.054 0.030 0.331

Comfortable.Communities 16.569 14.478 16.940 14.649 -0.026 -0.790 0.430 0.041 0.070

Financially.Stretched 13.914 12.671 14.048 12.545 -0.011 -0.338 0.736 0.027 0.481

Urban.Adversity 12.180 14.073 12.145 13.689 0.003 0.080 0.936 0.019 0.870

Not.Private.Households 6.352 5.888 6.277 5.482 0.013 0.429 0.668 0.018 0.911

Not.Found 27.456 18.430 27.242 17.796 0.012 0.379 0.705 0.022 0.725

ewLondonkm 3.867 1.510 3.890 1.491 -0.016 -0.414 0.679 0.035 0.170

nsLondonkm 3.814 1.771 3.863 1.704 -0.028 -0.802 0.422 0.034 0.205

ahah.v2 22.755 7.229 22.118 6.493 0.088 2.505 0.012 0.044 0.048

Lemployment 0.976 1.076 1.064 1.037 -0.082 -2.559 0.011 0.043 0.052

raildist 0.258 1.010 0.301 0.985 -0.043 -1.367 0.172 0.038 0.115

[1] "Table S5 : ASDA"

$unw

tx.mn tx.sd ct.mn ct.sd std.eff.sz stat p ks ks.pval

discount.count 0.589 0.492 0.326 0.469 0.536 19.117 0.000 0.264 0.000

freezer.count 0.611 0.488 0.296 0.457 0.645 23.263 0.000 0.314 0.000

Budgens.Spar.count 0.216 0.412 0.200 0.400 0.039 1.390 0.165 0.016 0.904

Co.op.count 0.526 0.499 0.491 0.500 0.070 2.408 0.016 0.035 0.107

Morrisons.count 0.684 0.465 0.346 0.476 0.726 24.753 0.000 0.338 0.000

Sainsbury.count 0.807 0.394 0.502 0.500 0.773 22.692 0.000 0.305 0.000

Tesco.count 0.871 0.335 0.615 0.487 0.763 20.197 0.000 0.256 0.000

premium.count 0.526 0.499 0.400 0.490 0.251 8.803 0.000 0.125 0.000

Detached 8.081 6.922 16.784 12.140 -1.257 -28.484 0.000 0.368 0.000

SemiDetached 21.033 14.070 24.753 11.790 -0.264 -10.180 0.000 0.192 0.000

Terraced 27.088 17.065 24.184 13.379 0.170 6.792 0.000 0.133 0.000

Flats 43.798 24.911 34.279 23.034 0.382 13.893 0.000 0.171 0.000

Rooms1 2.055 2.469 1.494 2.430 0.227 7.950 0.000 0.172 0.000

Rooms23 24.947 12.286 20.417 10.814 0.369 13.802 0.000 0.170 0.000

Rooms45 51.102 10.467 50.864 8.344 0.023 0.899 0.368 0.089 0.000

Rooms6p 21.896 9.532 27.224 10.778 -0.559 -17.821 0.000 0.215 0.000

Affluent.Achievers 11.229 15.242 23.698 19.415 -0.818 -23.933 0.000 0.344 0.000

Rising.Prosperity 7.647 12.587 6.485 10.330 0.092 3.596 0.000 0.104 0.000

Comfortable.Communities 15.665 14.316 20.076 16.199 -0.308 -9.820 0.000 0.129 0.000

Financially.Stretched 15.058 12.921 11.752 11.475 0.256 9.528 0.000 0.153 0.000

Urban.Adversity 15.444 15.325 6.040 9.191 0.614 27.700 0.000 0.340 0.000

Not.Private.Households 6.784 5.988 5.850 5.288 0.156 5.826 0.000 0.087 0.000

Not.Found 28.174 18.525 26.099 17.564 0.112 4.012 0.000 0.068 0.000

ewLondonkm 3.936 1.447 3.928 1.372 0.005 0.194 0.847 0.104 0.000

nsLondonkm 3.938 1.831 3.872 1.386 0.036 1.469 0.142 0.193 0.000

ahah.v2 22.414 6.544 23.185 7.166 -0.118 -3.842 0.000 0.040 0.045

Lemployment 0.825 0.925 1.680 1.100 -0.925 -28.450 0.000 0.427 0.000

raildist 0.193 0.871 0.615 1.194 -0.484 -13.398 0.000 0.221 0.000

$es.mean.ATT

tx.mn tx.sd ct.mn ct.sd std.eff.sz stat p ks ks.pval

discount.count 0.589 0.492 0.530 0.499 0.120 2.774 0.006 0.059 0.046

freezer.count 0.611 0.488 0.548 0.498 0.130 3.006 0.003 0.063 0.027

Budgens.Spar.count 0.216 0.412 0.247 0.431 -0.075 -1.635 0.102 0.031 0.667

Co.op.count 0.526 0.499 0.572 0.495 -0.091 -2.123 0.034 0.046 0.208

Morrisons.count 0.684 0.465 0.610 0.488 0.159 3.659 0.000 0.074 0.006

Sainsbury.count 0.807 0.394 0.773 0.419 0.086 2.024 0.043 0.034 0.556

Tesco.count 0.871 0.335 0.827 0.379 0.132 3.099 0.002 0.044 0.237

premium.count 0.526 0.499 0.554 0.497 -0.056 -1.317 0.188 0.028 0.776

Detached 8.081 6.922 8.822 7.452 -0.107 -3.040 0.002 0.067 0.015

SemiDetached 21.033 14.070 21.720 13.207 -0.049 -1.141 0.254 0.073 0.006

Terraced 27.088 17.065 26.999 16.025 0.005 0.114 0.909 0.041 0.307

Flats 43.798 24.911 42.459 24.588 0.054 1.184 0.237 0.038 0.418

Rooms1 2.055 2.469 2.062 2.650 -0.003 -0.064 0.949 0.039 0.387

Rooms23 24.947 12.286 24.234 11.537 0.058 1.394 0.163 0.032 0.614

Rooms45 51.102 10.467 51.263 10.082 -0.015 -0.330 0.741 0.038 0.404

Rooms6p 21.896 9.532 22.441 9.652 -0.057 -1.395 0.163 0.029 0.747

Affluent.Achievers 11.229 15.242 13.902 16.183 -0.175 -4.375 0.000 0.116 0.000

Rising.Prosperity 7.647 12.587 8.048 12.358 -0.032 -0.806 0.420 0.091 0.000

Comfortable.Communities 15.665 14.316 16.574 13.968 -0.064 -1.471 0.141 0.064 0.025

Financially.Stretched 15.058 12.921 14.869 13.071 0.015 0.320 0.749 0.032 0.633

Urban.Adversity 15.444 15.325 12.459 13.382 0.195 4.309 0.000 0.112 0.000

Not.Private.Households 6.784 5.988 6.793 5.941 -0.002 -0.035 0.972 0.027 0.823

Not.Found 28.174 18.525 27.356 17.730 0.044 1.108 0.268 0.034 0.535

ewLondonkm 3.936 1.447 3.839 1.397 0.067 1.706 0.088 0.118 0.000

nsLondonkm 3.938 1.831 3.846 1.616 0.051 1.211 0.226 0.101 0.000

ahah.v2 22.414 6.544 21.653 6.474 0.116 2.994 0.003 0.079 0.002

Lemployment 0.825 0.925 0.942 0.966 -0.127 -3.202 0.001 0.094 0.000

raildist 0.193 0.871 0.195 0.929 -0.002 -0.040 0.968 0.034 0.543

$es.max.ATT

tx.mn tx.sd ct.mn ct.sd std.eff.sz stat p ks ks.pval

discount.count 0.589 0.492 0.533 0.499 0.114 2.451 0.014 0.056 0.104

freezer.count 0.611 0.488 0.550 0.498 0.124 2.682 0.007 0.061 0.064

Budgens.Spar.count 0.216 0.412 0.240 0.427 -0.058 -1.178 0.239 0.024 0.948

Co.op.count 0.526 0.499 0.561 0.496 -0.070 -1.493 0.136 0.035 0.618

Morrisons.count 0.684 0.465 0.612 0.487 0.154 3.324 0.001 0.071 0.017

Sainsbury.count 0.807 0.394 0.777 0.417 0.078 1.729 0.084 0.031 0.764

Tesco.count 0.871 0.335 0.830 0.375 0.121 2.709 0.007 0.041 0.419

premium.count 0.526 0.499 0.542 0.498 -0.033 -0.707 0.480 0.016 1.000

Detached 8.081 6.922 8.879 7.320 -0.115 -3.091 0.002 0.077 0.008

SemiDetached 21.033 14.070 22.323 13.329 -0.092 -1.940 0.052 0.087 0.002

Terraced 27.088 17.065 27.017 15.958 0.004 0.085 0.932 0.043 0.358

Flats 43.798 24.911 41.782 24.207 0.081 1.649 0.099 0.045 0.304

Rooms1 2.055 2.469 1.941 2.484 0.046 1.024 0.306 0.055 0.119

Rooms23 24.947 12.286 23.844 11.204 0.090 2.069 0.039 0.049 0.215

Rooms45 51.102 10.467 51.810 9.990 -0.068 -1.364 0.173 0.059 0.079

Rooms6p 21.896 9.532 22.405 9.224 -0.053 -1.295 0.195 0.035 0.623

Affluent.Achievers 11.229 15.242 13.863 15.936 -0.173 -4.078 0.000 0.120 0.000

Rising.Prosperity 7.647 12.587 7.541 11.629 0.008 0.220 0.826 0.081 0.004

Comfortable.Communities 15.665 14.316 17.266 14.377 -0.112 -2.286 0.022 0.076 0.009

Financially.Stretched 15.058 12.921 15.198 13.072 -0.011 -0.216 0.829 0.030 0.802

Urban.Adversity 15.444 15.325 12.672 13.559 0.181 3.685 0.000 0.107 0.000

Not.Private.Households 6.784 5.988 6.675 5.940 0.018 0.396 0.692 0.031 0.754

Not.Found 28.174 18.525 26.786 17.468 0.075 1.825 0.068 0.050 0.191

ewLondonkm 3.936 1.447 3.891 1.364 0.031 0.773 0.439 0.109 0.000

nsLondonkm 3.938 1.831 3.873 1.648 0.036 0.759 0.448 0.085 0.002

ahah.v2 22.414 6.544 21.543 6.134 0.133 3.433 0.001 0.084 0.003

Lemployment 0.825 0.925 0.956 0.942 -0.141 -3.478 0.001 0.098 0.000

raildist 0.193 0.871 0.234 0.904 -0.046 -1.096 0.273 0.036 0.559

$ks.mean.ATT

tx.mn tx.sd ct.mn ct.sd std.eff.sz stat p ks ks.pval

discount.count 0.589 0.492 0.531 0.499 0.118 2.678 0.007 0.058 0.059

freezer.count 0.611 0.488 0.549 0.498 0.127 2.902 0.004 0.062 0.036

Budgens.Spar.count 0.216 0.412 0.244 0.430 -0.069 -1.482 0.139 0.029 0.781

Co.op.count 0.526 0.499 0.570 0.495 -0.089 -2.034 0.042 0.044 0.248

Morrisons.count 0.684 0.465 0.610 0.488 0.159 3.604 0.000 0.074 0.007

Sainsbury.count 0.807 0.394 0.775 0.418 0.083 1.921 0.055 0.033 0.627

Tesco.count 0.871 0.335 0.828 0.378 0.129 3.001 0.003 0.043 0.277

premium.count 0.526 0.499 0.552 0.497 -0.052 -1.196 0.232 0.026 0.863

Detached 8.081 6.922 8.813 7.418 -0.106 -2.968 0.003 0.068 0.016

SemiDetached 21.033 14.070 21.821 13.250 -0.056 -1.281 0.200 0.075 0.006

Terraced 27.088 17.065 26.987 16.056 0.006 0.126 0.900 0.040 0.359

Flats 43.798 24.911 42.378 24.596 0.057 1.227 0.220 0.038 0.421

Rooms1 2.055 2.469 2.044 2.629 0.004 0.096 0.924 0.041 0.332

Rooms23 24.947 12.286 24.162 11.497 0.064 1.514 0.130 0.035 0.546

Rooms45 51.102 10.467 51.382 10.086 -0.027 -0.565 0.572 0.043 0.292

Rooms6p 21.896 9.532 22.411 9.583 -0.054 -1.313 0.189 0.029 0.760

Affluent.Achievers 11.229 15.242 13.857 16.103 -0.172 -4.256 0.000 0.115 0.000

Rising.Prosperity 7.647 12.587 7.985 12.275 -0.027 -0.683 0.495 0.089 0.001

Comfortable.Communities 15.665 14.316 16.686 14.054 -0.071 -1.609 0.108 0.064 0.025

Financially.Stretched 15.058 12.921 14.937 13.097 0.009 0.199 0.842 0.030 0.712

Urban.Adversity 15.444 15.325 12.511 13.424 0.191 4.155 0.000 0.111 0.000

Not.Private.Households 6.784 5.988 6.771 5.945 0.002 0.051 0.960 0.026 0.877

Not.Found 28.174 18.525 27.253 17.679 0.050 1.239 0.215 0.037 0.460

ewLondonkm 3.936 1.447 3.845 1.394 0.063 1.589 0.112 0.117 0.000

nsLondonkm 3.938 1.831 3.847 1.627 0.050 1.162 0.245 0.098 0.000

ahah.v2 22.414 6.544 21.653 6.415 0.116 2.994 0.003 0.080 0.002

Lemployment 0.825 0.925 0.944 0.960 -0.128 -3.227 0.001 0.095 0.000

raildist 0.193 0.871 0.203 0.924 -0.011 -0.272 0.785 0.031 0.678

$ks.max.ATT

tx.mn tx.sd ct.mn ct.sd std.eff.sz stat p ks ks.pval

discount.count 0.589 0.492 0.531 0.499 0.118 2.664 0.008 0.058 0.061

freezer.count 0.611 0.488 0.549 0.498 0.126 2.858 0.004 0.062 0.041

Budgens.Spar.count 0.216 0.412 0.244 0.430 -0.068 -1.439 0.150 0.028 0.810

Co.op.count 0.526 0.499 0.569 0.495 -0.087 -1.973 0.049 0.044 0.280

Morrisons.count 0.684 0.465 0.609 0.488 0.161 3.609 0.000 0.075 0.006

Sainsbury.count 0.807 0.394 0.774 0.419 0.085 1.955 0.051 0.034 0.601

Tesco.count 0.871 0.335 0.828 0.377 0.128 2.960 0.003 0.043 0.297

premium.count 0.526 0.499 0.549 0.498 -0.045 -1.032 0.302 0.023 0.950

Detached 8.081 6.922 8.830 7.404 -0.108 -3.015 0.003 0.070 0.012

SemiDetached 21.033 14.070 21.932 13.265 -0.064 -1.443 0.149 0.078 0.004

Terraced 27.088 17.065 27.033 16.056 0.003 0.068 0.946 0.041 0.341

Flats 43.798 24.911 42.205 24.543 0.064 1.360 0.174 0.041 0.362

Rooms1 2.055 2.469 2.024 2.601 0.013 0.281 0.779 0.044 0.270

Rooms23 24.947 12.286 24.082 11.464 0.070 1.654 0.098 0.037 0.473

Rooms45 51.102 10.467 51.473 10.085 -0.035 -0.739 0.460 0.046 0.220

Rooms6p 21.896 9.532 22.421 9.538 -0.055 -1.338 0.181 0.030 0.746

Affluent.Achievers 11.229 15.242 13.839 16.061 -0.171 -4.201 0.000 0.115 0.000

Rising.Prosperity 7.647 12.587 7.909 12.186 -0.021 -0.529 0.597 0.087 0.001

Comfortable.Communities 15.665 14.316 16.783 14.094 -0.078 -1.735 0.083 0.067 0.021

Financially.Stretched 15.058 12.921 15.013 13.109 0.003 0.073 0.942 0.028 0.814

Urban.Adversity 15.444 15.325 12.566 13.469 0.188 4.021 0.000 0.110 0.000

Not.Private.Households 6.784 5.988 6.749 5.936 0.006 0.134 0.894 0.024 0.926

Not.Found 28.174 18.525 27.142 17.630 0.056 1.386 0.166 0.040 0.382

ewLondonkm 3.936 1.447 3.856 1.387 0.056 1.394 0.163 0.114 0.000

nsLondonkm 3.938 1.831 3.855 1.629 0.045 1.044 0.296 0.094 0.000

ahah.v2 22.414 6.544 21.636 6.363 0.119 3.064 0.002 0.081 0.002

Lemployment 0.825 0.925 0.948 0.958 -0.133 -3.331 0.001 0.097 0.000

raildist 0.193 0.871 0.209 0.922 -0.018 -0.443 0.658 0.032 0.650

[1] "Table S6 : Morrisons"

$unw

tx.mn tx.sd ct.mn ct.sd std.eff.sz stat p ks ks.pval

discount.count 0.598 0.490 0.363 0.481 0.478 17.331 0.000 0.235 0.017

freezer.count 0.625 0.484 0.335 0.472 0.599 21.788 0.000 0.290 0.017

Budgens.Spar.count 0.229 0.420 0.186 0.389 0.104 3.883 0.000 0.044 0.028

Co.op.count 0.525 0.499 0.499 0.500 0.053 1.886 0.059 0.026 0.329

Asda.count 0.781 0.414 0.466 0.499 0.761 24.339 0.000 0.315 0.017

Sainsbury.count 0.794 0.405 0.576 0.494 0.538 17.062 0.000 0.218 0.017

Tesco.count 0.864 0.343 0.671 0.470 0.560 16.428 0.000 0.192 0.017

premium.count 0.562 0.496 0.377 0.485 0.372 13.501 0.000 0.185 0.017

Detached 7.956 6.887 15.353 11.781 -1.074 -26.659 0.000 0.301 0.017

SemiDetached 20.387 13.968 24.905 12.217 -0.323 -12.447 0.000 0.192 0.017

Terraced 26.621 16.652 25.318 14.865 0.078 2.979 0.003 0.099 0.017

Flats 45.036 25.490 34.423 22.217 0.416 16.048 0.000 0.174 0.017

Rooms1 2.196 2.714 1.415 2.031 0.288 11.884 0.000 0.171 0.017

Rooms23 25.606 12.636 20.396 10.362 0.412 16.364 0.000 0.178 0.017

Rooms45 50.772 10.593 51.334 8.564 -0.053 -2.119 0.034 0.082 0.017

Rooms6p 21.427 9.617 26.855 10.381 -0.564 -19.351 0.000 0.202 0.017

Affluent.Achievers 11.420 15.486 21.170 19.210 -0.630 -19.766 0.000 0.269 0.017

Rising.Prosperity 8.043 12.778 6.186 10.425 0.145 5.782 0.000 0.076 0.017

Comfortable.Communities 15.276 14.182 19.771 15.988 -0.317 -10.582 0.000 0.130 0.017

Financially.Stretched 14.583 12.670 12.969 12.276 0.127 4.645 0.000 0.103 0.017

Urban.Adversity 15.403 15.186 7.814 11.467 0.500 20.574 0.000 0.284 0.017

Not.Private.Households 7.062 6.128 5.662 5.155 0.229 8.962 0.000 0.121 0.017

Not.Found 28.214 18.362 26.428 17.974 0.097 3.528 0.000 0.065 0.017

ewLondonkm 3.893 1.458 3.985 1.369 -0.063 -2.339 0.019 0.081 0.017

nsLondonkm 3.944 1.814 3.877 1.503 0.037 1.464 0.143 0.150 0.017

ahah.v2 22.529 6.998 22.896 6.488 -0.052 -1.959 0.050 0.056 0.018

Lemployment 0.871 1.009 1.464 1.060 -0.588 -20.486 0.000 0.263 0.017

raildist 0.166 0.919 0.573 1.092 -0.443 -14.299 0.000 0.181 0.017

$es.mean.ATT

tx.mn tx.sd ct.mn ct.sd std.eff.sz stat p ks ks.pval

discount.count 0.598 0.490 0.580 0.494 0.036 1.024 0.306 0.017 0.965

freezer.count 0.625 0.484 0.592 0.492 0.068 1.979 0.048 0.033 0.346

Budgens.Spar.count 0.229 0.420 0.211 0.408 0.042 1.208 0.227 0.018 0.959

Co.op.count 0.525 0.499 0.530 0.499 -0.010 -0.273 0.785 0.005 1.000

Asda.count 0.781 0.414 0.752 0.432 0.069 2.115 0.034 0.029 0.525

Sainsbury.count 0.794 0.405 0.792 0.406 0.003 0.100 0.920 0.001 1.000

Tesco.count 0.864 0.343 0.840 0.366 0.068 1.927 0.054 0.023 0.779

premium.count 0.562 0.496 0.525 0.499 0.074 2.095 0.036 0.037 0.226

Detached 7.956 6.887 8.299 7.203 -0.050 -1.637 0.102 0.037 0.226

SemiDetached 20.387 13.968 21.084 13.602 -0.050 -1.373 0.170 0.046 0.070

Terraced 26.621 16.652 26.554 16.519 0.004 0.109 0.913 0.022 0.830

Flats 45.036 25.490 44.063 24.942 0.038 1.004 0.315 0.027 0.582

Rooms1 2.196 2.714 2.056 2.432 0.052 1.515 0.130 0.031 0.407

Rooms23 25.606 12.636 24.979 12.017 0.050 1.278 0.201 0.030 0.455

Rooms45 50.772 10.593 50.831 9.739 -0.006 -0.155 0.877 0.032 0.398

Rooms6p 21.427 9.617 22.134 9.449 -0.074 -2.054 0.040 0.045 0.075

Affluent.Achievers 11.420 15.486 12.273 15.649 -0.055 -1.716 0.086 0.054 0.018

Rising.Prosperity 8.043 12.778 7.496 12.261 0.043 1.250 0.211 0.032 0.389

Comfortable.Communities 15.276 14.182 15.395 14.126 -0.008 -0.243 0.808 0.025 0.677

Financially.Stretched 14.583 12.670 14.616 12.447 -0.003 -0.078 0.938 0.020 0.888

Urban.Adversity 15.403 15.186 14.502 14.973 0.059 1.522 0.128 0.037 0.220

Not.Private.Households 7.062 6.128 6.886 6.299 0.029 0.736 0.461 0.033 0.352

Not.Found 28.214 18.362 28.832 18.416 -0.034 -0.951 0.341 0.032 0.369

ewLondonkm 3.893 1.458 3.927 1.445 -0.023 -0.670 0.503 0.051 0.029

nsLondonkm 3.944 1.814 3.928 1.727 0.009 0.254 0.800 0.056 0.013

ahah.v2 22.529 6.998 22.403 6.735 0.018 0.459 0.646 0.034 0.308

Lemployment 0.871 1.009 0.933 0.994 -0.061 -1.712 0.087 0.036 0.255

raildist 0.166 0.919 0.165 0.889 0.002 0.047 0.962 0.034 0.323

$es.max.ATT

tx.mn tx.sd ct.mn ct.sd std.eff.sz stat p ks ks.pval

discount.count 0.598 0.490 0.580 0.494 0.036 1.036 0.300 0.018 0.961

freezer.count 0.625 0.484 0.592 0.492 0.069 1.993 0.046 0.033 0.338

Budgens.Spar.count 0.229 0.420 0.211 0.408 0.042 1.211 0.226 0.018 0.958

Co.op.count 0.525 0.499 0.530 0.499 -0.011 -0.299 0.765 0.005 1.000

Asda.count 0.781 0.414 0.752 0.432 0.070 2.125 0.034 0.029 0.519

Sainsbury.count 0.794 0.405 0.792 0.406 0.003 0.100 0.921 0.001 1.000

Tesco.count 0.864 0.343 0.840 0.366 0.068 1.935 0.053 0.023 0.774

premium.count 0.562 0.496 0.525 0.499 0.074 2.087 0.037 0.037 0.230

Detached 7.956 6.887 8.299 7.204 -0.050 -1.636 0.102 0.037 0.227

SemiDetached 20.387 13.968 21.077 13.598 -0.049 -1.361 0.174 0.046 0.071

Terraced 26.621 16.652 26.555 16.526 0.004 0.107 0.915 0.022 0.830

Flats 45.036 25.490 44.069 24.943 0.038 0.999 0.318 0.027 0.587

Rooms1 2.196 2.714 2.056 2.432 0.052 1.518 0.129 0.031 0.407

Rooms23 25.606 12.636 24.979 12.015 0.050 1.280 0.201 0.030 0.456

Rooms45 50.772 10.593 50.830 9.740 -0.006 -0.154 0.878 0.032 0.399

Rooms6p 21.427 9.617 22.135 9.451 -0.074 -2.058 0.040 0.045 0.075

Affluent.Achievers 11.420 15.486 12.282 15.651 -0.056 -1.735 0.083 0.055 0.016

Rising.Prosperity 8.043 12.778 7.500 12.264 0.043 1.241 0.215 0.032 0.397

Comfortable.Communities 15.276 14.182 15.396 14.122 -0.009 -0.247 0.805 0.026 0.668

Financially.Stretched 14.583 12.670 14.620 12.450 -0.003 -0.086 0.931 0.020 0.890

Urban.Adversity 15.403 15.186 14.490 14.963 0.060 1.545 0.122 0.037 0.214

Not.Private.Households 7.062 6.128 6.885 6.298 0.029 0.742 0.458 0.033 0.352

Not.Found 28.214 18.362 28.827 18.417 -0.033 -0.944 0.345 0.032 0.374

ewLondonkm 3.893 1.458 3.927 1.445 -0.023 -0.662 0.508 0.051 0.030

nsLondonkm 3.944 1.814 3.927 1.727 0.009 0.259 0.795 0.056 0.013

ahah.v2 22.529 6.998 22.400 6.734 0.018 0.472 0.637 0.034 0.304

Lemployment 0.871 1.009 0.933 0.994 -0.061 -1.719 0.086 0.036 0.253

raildist 0.166 0.919 0.164 0.889 0.002 0.050 0.960 0.034 0.322

$ks.mean.ATT

tx.mn tx.sd ct.mn ct.sd std.eff.sz stat p ks ks.pval

discount.count 0.598 0.490 0.580 0.494 0.036 1.030 0.303 0.018 0.963

freezer.count 0.625 0.484 0.592 0.492 0.068 1.983 0.047 0.033 0.344

Budgens.Spar.count 0.229 0.420 0.211 0.408 0.042 1.208 0.227 0.018 0.958

Co.op.count 0.525 0.499 0.530 0.499 -0.010 -0.270 0.787 0.005 1.000

Asda.count 0.781 0.414 0.752 0.432 0.069 2.116 0.034 0.029 0.524

Sainsbury.count 0.794 0.405 0.792 0.406 0.003 0.100 0.920 0.001 1.000

Tesco.count 0.864 0.343 0.840 0.366 0.068 1.930 0.054 0.023 0.777

premium.count 0.562 0.496 0.525 0.499 0.074 2.095 0.036 0.037 0.226

Detached 7.956 6.887 8.299 7.203 -0.050 -1.638 0.101 0.037 0.225

SemiDetached 20.387 13.968 21.086 13.601 -0.050 -1.377 0.168 0.046 0.069

Terraced 26.621 16.652 26.555 16.519 0.004 0.108 0.914 0.022 0.829

Flats 45.036 25.490 44.060 24.940 0.038 1.008 0.314 0.027 0.579

Rooms1 2.196 2.714 2.056 2.432 0.052 1.517 0.129 0.031 0.407

Rooms23 25.606 12.636 24.978 12.017 0.050 1.280 0.200 0.030 0.454

Rooms45 50.772 10.593 50.830 9.740 -0.005 -0.153 0.878 0.032 0.400

Rooms6p 21.427 9.617 22.136 9.452 -0.074 -2.060 0.039 0.045 0.075

Affluent.Achievers 11.420 15.486 12.272 15.649 -0.055 -1.715 0.086 0.054 0.018

Rising.Prosperity 8.043 12.778 7.495 12.259 0.043 1.253 0.210 0.032 0.390

Comfortable.Communities 15.276 14.182 15.397 14.125 -0.009 -0.247 0.805 0.025 0.673

Financially.Stretched 14.583 12.670 14.622 12.450 -0.003 -0.092 0.927 0.021 0.885

Urban.Adversity 15.403 15.186 14.502 14.971 0.059 1.523 0.128 0.037 0.221

Not.Private.Households 7.062 6.128 6.885 6.298 0.029 0.739 0.460 0.033 0.351

Not.Found 28.214 18.362 28.827 18.414 -0.033 -0.944 0.345 0.032 0.373

ewLondonkm 3.893 1.458 3.927 1.445 -0.023 -0.665 0.506 0.051 0.030

nsLondonkm 3.944 1.814 3.927 1.728 0.009 0.262 0.793 0.056 0.013

ahah.v2 22.529 6.998 22.403 6.734 0.018 0.460 0.645 0.034 0.307

Lemployment 0.871 1.009 0.933 0.994 -0.061 -1.713 0.087 0.036 0.256

raildist 0.166 0.919 0.165 0.889 0.002 0.046 0.963 0.034 0.322

$ks.max.ATT

tx.mn tx.sd ct.mn ct.sd std.eff.sz stat p ks ks.pval

discount.count 0.598 0.490 0.581 0.493 0.033 0.954 0.340 0.016 0.982

freezer.count 0.625 0.484 0.591 0.492 0.071 2.037 0.042 0.034 0.312

Budgens.Spar.count 0.229 0.420 0.211 0.408 0.043 1.234 0.217 0.018 0.952

Co.op.count 0.525 0.499 0.529 0.499 -0.008 -0.213 0.831 0.004 1.000

Asda.count 0.781 0.414 0.750 0.433 0.075 2.273 0.023 0.031 0.426

Sainsbury.count 0.794 0.405 0.793 0.405 0.002 0.068 0.946 0.001 1.000

Tesco.count 0.864 0.343 0.840 0.367 0.068 1.932 0.053 0.024 0.771

premium.count 0.562 0.496 0.525 0.499 0.074 2.080 0.038 0.037 0.233

Detached 7.956 6.887 8.325 7.199 -0.054 -1.746 0.081 0.039 0.188

SemiDetached 20.387 13.968 21.187 13.619 -0.057 -1.560 0.119 0.048 0.054

Terraced 26.621 16.652 26.568 16.467 0.003 0.086 0.932 0.022 0.830

Flats 45.036 25.490 43.921 24.904 0.044 1.145 0.252 0.030 0.483

Rooms1 2.196 2.714 2.041 2.415 0.057 1.685 0.092 0.032 0.381

Rooms23 25.606 12.636 24.885 12.010 0.057 1.459 0.145 0.034 0.315

Rooms45 50.772 10.593 50.913 9.730 -0.013 -0.370 0.711 0.034 0.327

Rooms6p 21.427 9.617 22.161 9.431 -0.076 -2.122 0.034 0.048 0.055

Affluent.Achievers 11.420 15.486 12.320 15.696 -0.058 -1.791 0.073 0.054 0.019

Rising.Prosperity 8.043 12.778 7.407 12.097 0.050 1.467 0.142 0.033 0.344

Comfortable.Communities 15.276 14.182 15.447 14.127 -0.012 -0.349 0.727 0.027 0.603

Financially.Stretched 14.583 12.670 14.653 12.460 -0.006 -0.163 0.871 0.022 0.830

Urban.Adversity 15.403 15.186 14.525 14.998 0.058 1.467 0.142 0.037 0.220

Not.Private.Households 7.062 6.128 6.883 6.278 0.029 0.745 0.456 0.033 0.342

Not.Found 28.214 18.362 28.765 18.396 -0.030 -0.842 0.400 0.032 0.411

ewLondonkm 3.893 1.458 3.934 1.434 -0.028 -0.805 0.421 0.050 0.039

nsLondonkm 3.944 1.814 3.936 1.720 0.004 0.120 0.904 0.055 0.018

ahah.v2 22.529 6.998 22.364 6.706 0.024 0.602 0.547 0.036 0.266

Lemployment 0.871 1.009 0.936 0.995 -0.065 -1.798 0.072 0.038 0.204

raildist 0.166 0.919 0.170 0.891 -0.004 -0.118 0.906 0.030 0.453

[1] "Table S7 : Sainsbury's"

$unw

tx.mn tx.sd ct.mn ct.sd std.eff.sz stat p ks ks.pval

discount.count 0.560 0.496 0.346 0.476 0.432 14.742 0.000 0.214 0.000

freezer.count 0.582 0.493 0.304 0.460 0.564 19.603 0.000 0.278 0.000

Budgens.Spar.count 0.218 0.413 0.191 0.393 0.065 2.244 0.025 0.027 0.389

Co.op.count 0.546 0.498 0.438 0.496 0.216 7.179 0.000 0.108 0.000

Asda.count 0.743 0.437 0.411 0.492 0.761 23.143 0.000 0.332 0.000

Morrisons.count 0.640 0.480 0.386 0.487 0.530 17.397 0.000 0.254 0.000

Tesco.count 0.863 0.344 0.588 0.492 0.799 20.135 0.000 0.275 0.000

premium.count 0.593 0.491 0.221 0.415 0.758 28.093 0.000 0.372 0.000

Detached 8.465 7.416 17.491 12.247 -1.217 -27.173 0.000 0.351 0.000

SemiDetached 20.166 13.647 27.444 11.351 -0.533 -19.965 0.000 0.300 0.000

Terraced 24.686 15.848 29.217 15.596 -0.286 -9.587 0.000 0.155 0.000

Flats 46.683 24.640 25.849 17.631 0.846 34.539 0.000 0.400 0.000

Rooms1 2.297 2.748 0.831 1.108 0.533 27.452 0.000 0.338 0.000

Rooms23 26.109 12.179 16.893 8.544 0.757 31.242 0.000 0.353 0.000

Rooms45 49.660 10.013 54.162 8.348 -0.450 -16.807 0.000 0.210 0.000

Rooms6p 21.934 9.863 28.114 10.043 -0.627 -20.518 0.000 0.255 0.000

Affluent.Achievers 13.471 17.026 20.790 18.739 -0.430 -13.308 0.000 0.229 0.000

Rising.Prosperity 9.187 13.495 2.702 3.728 0.481 26.755 0.000 0.277 0.000

Comfortable.Communities 14.965 14.036 22.508 16.320 -0.537 -15.969 0.000 0.221 0.000

Financially.Stretched 12.829 11.823 16.308 13.712 -0.294 -8.760 0.000 0.128 0.000

Urban.Adversity 13.774 14.967 8.185 11.291 0.373 14.810 0.000 0.216 0.000

Not.Private.Households 6.723 6.051 5.820 4.986 0.149 5.617 0.000 0.076 0.000

Not.Found 29.051 18.596 23.686 16.708 0.289 10.286 0.000 0.177 0.000

ewLondonkm 3.682 1.526 4.516 0.902 -0.546 -24.537 0.000 0.260 0.000

nsLondonkm 3.666 1.786 4.491 1.249 -0.462 -19.102 0.000 0.250 0.000

ahah.v2 22.873 6.910 22.262 6.457 0.088 3.070 0.002 0.051 0.007

Lemployment 0.815 0.934 1.860 1.015 -1.118 -34.953 0.000 0.465 0.000

raildist 0.129 0.877 0.842 1.142 -0.813 -22.105 0.000 0.310 0.000

$es.mean.ATT

tx.mn tx.sd ct.mn ct.sd std.eff.sz stat p ks ks.pval

discount.count 0.560 0.496 0.536 0.499 0.048 0.632 0.528 0.024 1.000

freezer.count 0.582 0.493 0.535 0.499 0.097 1.300 0.194 0.048 0.807

Budgens.Spar.count 0.218 0.413 0.200 0.400 0.044 0.574 0.566 0.018 1.000

Co.op.count 0.546 0.498 0.506 0.500 0.081 1.063 0.288 0.040 0.931

Asda.count 0.743 0.437 0.720 0.449 0.054 0.865 0.387 0.023 1.000

Morrisons.count 0.640 0.480 0.620 0.486 0.042 0.599 0.549 0.020 1.000

Tesco.count 0.863 0.344 0.843 0.364 0.058 0.920 0.358 0.020 1.000

premium.count 0.593 0.491 0.515 0.500 0.159 2.080 0.038 0.078 0.229

Detached 8.465 7.416 9.924 7.662 -0.197 -3.476 0.001 0.150 0.001

SemiDetached 20.166 13.647 22.249 11.782 -0.153 -2.234 0.026 0.132 0.004

Terraced 24.686 15.848 27.837 14.384 -0.199 -3.053 0.002 0.141 0.002

Flats 46.683 24.640 39.990 18.792 0.272 4.161 0.000 0.158 0.000

Rooms1 2.297 2.748 1.706 1.725 0.215 4.178 0.000 0.133 0.004

Rooms23 26.109 12.179 24.145 9.961 0.161 2.317 0.021 0.111 0.026

Rooms45 49.660 10.013 51.052 8.715 -0.139 -2.373 0.018 0.134 0.004

Rooms6p 21.934 9.863 23.096 8.756 -0.118 -1.886 0.059 0.105 0.040

Affluent.Achievers 13.471 17.026 14.503 16.293 -0.061 -0.950 0.342 0.083 0.170

Rising.Prosperity 9.187 13.495 5.856 6.177 0.247 5.415 0.000 0.112 0.024

Comfortable.Communities 14.965 14.036 17.234 12.458 -0.162 -2.645 0.008 0.181 0.000

Financially.Stretched 12.829 11.823 13.599 10.938 -0.065 -1.148 0.251 0.111 0.026

Urban.Adversity 13.774 14.967 13.838 14.659 -0.004 -0.042 0.967 0.044 0.884

Not.Private.Households 6.723 6.051 6.622 5.838 0.017 0.276 0.783 0.057 0.598

Not.Found 29.051 18.596 28.348 17.673 0.038 0.456 0.648 0.038 0.960

ewLondonkm 3.682 1.526 4.004 1.043 -0.211 -3.743 0.000 0.154 0.000

nsLondonkm 3.666 1.786 3.846 1.583 -0.101 -1.341 0.180 0.079 0.226

ahah.v2 22.873 6.910 21.413 5.454 0.211 2.721 0.007 0.120 0.013

Lemployment 0.815 0.934 0.964 0.925 -0.159 -2.434 0.015 0.075 0.276

raildist 0.129 0.877 0.286 0.810 -0.179 -3.340 0.001 0.109 0.030

$es.max.ATT

tx.mn tx.sd ct.mn ct.sd std.eff.sz stat p ks ks.pval

discount.count 0.560 0.496 0.541 0.498 0.039 0.535 0.593 0.019 1.000

freezer.count 0.582 0.493 0.532 0.499 0.103 1.451 0.147 0.051 0.683

Budgens.Spar.count 0.218 0.413 0.202 0.402 0.038 0.534 0.593 0.016 1.000

Co.op.count 0.546 0.498 0.509 0.500 0.074 1.022 0.307 0.037 0.950

Asda.count 0.743 0.437 0.722 0.448 0.048 0.823 0.411 0.021 1.000

Morrisons.count 0.640 0.480 0.620 0.486 0.042 0.623 0.533 0.020 1.000

Tesco.count 0.863 0.344 0.850 0.358 0.038 0.660 0.509 0.013 1.000

premium.count 0.593 0.491 0.521 0.500 0.147 2.033 0.042 0.072 0.257

Detached 8.465 7.416 9.840 7.632 -0.185 -3.401 0.001 0.149 0.000

SemiDetached 20.166 13.647 22.381 11.880 -0.162 -2.505 0.012 0.133 0.002

Terraced 24.686 15.848 27.718 14.425 -0.191 -3.036 0.002 0.134 0.002

Flats 46.683 24.640 40.061 18.658 0.269 4.357 0.000 0.163 0.000

Rooms1 2.297 2.748 1.729 1.777 0.207 3.959 0.000 0.125 0.004

Rooms23 26.109 12.179 24.093 9.905 0.166 2.526 0.012 0.117 0.009

Rooms45 49.660 10.013 51.026 8.832 -0.136 -2.346 0.019 0.132 0.002

Rooms6p 21.934 9.863 23.151 8.775 -0.123 -2.041 0.041 0.103 0.031

Affluent.Achievers 13.471 17.026 14.571 16.324 -0.065 -1.059 0.290 0.084 0.125

Rising.Prosperity 9.187 13.495 5.803 6.173 0.251 5.787 0.000 0.111 0.016

Comfortable.Communities 14.965 14.036 17.354 12.554 -0.170 -2.856 0.004 0.178 0.000

Financially.Stretched 12.829 11.823 13.721 10.988 -0.075 -1.392 0.164 0.109 0.020

Urban.Adversity 13.774 14.967 13.700 14.383 0.005 0.055 0.956 0.043 0.857

Not.Private.Households 6.723 6.051 6.635 6.011 0.015 0.234 0.815 0.051 0.670

Not.Found 29.051 18.596 28.217 17.526 0.045 0.598 0.550 0.036 0.954

ewLondonkm 3.682 1.526 4.018 1.035 -0.220 -4.206 0.000 0.158 0.000

nsLondonkm 3.666 1.786 3.856 1.576 -0.106 -1.494 0.135 0.082 0.144

ahah.v2 22.873 6.910 21.267 5.363 0.232 3.352 0.001 0.130 0.003

Lemployment 0.815 0.934 0.952 0.927 -0.147 -2.281 0.023 0.070 0.282

raildist 0.129 0.877 0.294 0.810 -0.188 -3.624 0.000 0.110 0.018

$ks.mean.ATT

tx.mn tx.sd ct.mn ct.sd std.eff.sz stat p ks ks.pval

discount.count 0.560 0.496 0.540 0.499 0.039 0.560 0.576 0.020 1.000

freezer.count 0.582 0.493 0.528 0.499 0.110 1.571 0.116 0.054 0.579

Budgens.Spar.count 0.218 0.413 0.202 0.402 0.039 0.556 0.578 0.016 1.000

Co.op.count 0.546 0.498 0.509 0.500 0.075 1.060 0.289 0.037 0.933

Asda.count 0.743 0.437 0.723 0.448 0.046 0.799 0.425 0.020 1.000

Morrisons.count 0.640 0.480 0.620 0.486 0.042 0.636 0.525 0.020 1.000

Tesco.count 0.863 0.344 0.852 0.355 0.031 0.553 0.580 0.011 1.000

premium.count 0.593 0.491 0.523 0.500 0.143 2.033 0.042 0.070 0.258

Detached 8.465 7.416 9.851 7.640 -0.187 -3.474 0.001 0.149 0.000

SemiDetached 20.166 13.647 22.438 11.896 -0.166 -2.633 0.008 0.134 0.001

Terraced 24.686 15.848 27.722 14.440 -0.192 -3.096 0.002 0.133 0.001

Flats 46.683 24.640 39.989 18.585 0.272 4.571 0.000 0.167 0.000

Rooms1 2.297 2.748 1.732 1.790 0.205 3.947 0.000 0.123 0.004

Rooms23 26.109 12.179 24.028 9.871 0.171 2.690 0.007 0.119 0.006

Rooms45 49.660 10.013 51.034 8.872 -0.137 -2.376 0.018 0.131 0.002

Rooms6p 21.934 9.863 23.206 8.764 -0.129 -2.177 0.030 0.103 0.025

Affluent.Achievers 13.471 17.026 14.636 16.376 -0.068 -1.140 0.254 0.083 0.117

Rising.Prosperity 9.187 13.495 5.783 6.161 0.252 5.952 0.000 0.111 0.013

Comfortable.Communities 14.965 14.036 17.372 12.566 -0.172 -2.933 0.003 0.177 0.000

Financially.Stretched 12.829 11.823 13.743 10.985 -0.077 -1.458 0.145 0.108 0.017

Urban.Adversity 13.774 14.967 13.679 14.350 0.006 0.073 0.942 0.041 0.864

Not.Private.Households 6.723 6.051 6.656 6.113 0.011 0.172 0.864 0.050 0.671

Not.Found 29.051 18.596 28.131 17.458 0.049 0.687 0.492 0.036 0.952

ewLondonkm 3.682 1.526 4.026 1.031 -0.225 -4.420 0.000 0.161 0.000

nsLondonkm 3.666 1.786 3.860 1.579 -0.109 -1.553 0.120 0.083 0.114

ahah.v2 22.873 6.910 21.211 5.342 0.241 3.573 0.000 0.132 0.002

Lemployment 0.815 0.934 0.950 0.926 -0.145 -2.303 0.021 0.069 0.287

raildist 0.129 0.877 0.296 0.811 -0.191 -3.725 0.000 0.109 0.015

$ks.max.ATT

tx.mn tx.sd ct.mn ct.sd std.eff.sz stat p ks ks.pval

discount.count 0.560 0.496 0.541 0.498 0.038 0.575 0.565 0.019 1.000

freezer.count 0.582 0.493 0.526 0.499 0.115 1.731 0.084 0.057 0.451

Budgens.Spar.count 0.218 0.413 0.204 0.403 0.035 0.524 0.601 0.014 1.000

Co.op.count 0.546 0.498 0.507 0.500 0.078 1.163 0.245 0.039 0.878

Asda.count 0.743 0.437 0.720 0.449 0.054 0.969 0.333 0.024 0.999

Morrisons.count 0.640 0.480 0.615 0.487 0.052 0.817 0.414 0.025 0.998

Tesco.count 0.863 0.344 0.855 0.352 0.022 0.418 0.676 0.007 1.000

premium.count 0.593 0.491 0.524 0.500 0.140 2.108 0.035 0.069 0.225

Detached 8.465 7.416 9.915 7.691 -0.195 -3.715 0.000 0.149 0.000

SemiDetached 20.166 13.647 22.600 12.013 -0.178 -2.973 0.003 0.136 0.000

Terraced 24.686 15.848 27.601 14.492 -0.184 -3.139 0.002 0.127 0.001

Flats 46.683 24.640 39.883 18.578 0.276 4.938 0.000 0.170 0.000

Rooms1 2.297 2.748 1.720 1.813 0.210 4.076 0.000 0.121 0.003

Rooms23 26.109 12.179 23.878 9.850 0.183 3.056 0.002 0.124 0.002

Rooms45 49.660 10.013 51.162 8.960 -0.150 -2.664 0.008 0.134 0.001

Rooms6p 21.934 9.863 23.241 8.766 -0.132 -2.354 0.019 0.101 0.020

Affluent.Achievers 13.471 17.026 14.827 16.521 -0.080 -1.375 0.169 0.085 0.072

Rising.Prosperity 9.187 13.495 5.708 6.133 0.258 6.439 0.000 0.111 0.007

Comfortable.Communities 14.965 14.036 17.434 12.716 -0.176 -3.094 0.002 0.173 0.000

Financially.Stretched 12.829 11.823 13.816 11.075 -0.083 -1.638 0.102 0.104 0.015

Urban.Adversity 13.774 14.967 13.462 14.156 0.021 0.269 0.788 0.035 0.939

Not.Private.Households 6.723 6.051 6.679 6.208 0.007 0.113 0.910 0.045 0.732

Not.Found 29.051 18.596 28.074 17.397 0.053 0.792 0.428 0.034 0.948

ewLondonkm 3.682 1.526 4.041 1.028 -0.235 -4.901 0.000 0.163 0.000

nsLondonkm 3.666 1.786 3.883 1.571 -0.121 -1.836 0.066 0.093 0.038

ahah.v2 22.873 6.910 21.103 5.271 0.256 4.209 0.000 0.138 0.000

Lemployment 0.815 0.934 0.952 0.924 -0.147 -2.466 0.014 0.065 0.287

raildist 0.129 0.877 0.298 0.816 -0.192 -3.859 0.000 0.105 0.013

[1] "Table S8 : Tesco"

$unw

tx.mn tx.sd ct.mn ct.sd std.eff.sz stat p ks ks.pval

discount.count 0.560 0.496 0.267 0.443 0.590 19.279 0.000 0.293 0.000

freezer.count 0.571 0.495 0.241 0.428 0.668 22.322 0.000 0.330 0.000

Budgens.Spar.count 0.220 0.414 0.177 0.381 0.104 3.318 0.001 0.043 0.069

Co.op.count 0.530 0.499 0.457 0.498 0.146 4.391 0.000 0.073 0.000

Asda.count 0.718 0.450 0.377 0.485 0.760 21.463 0.000 0.342 0.000

Morrisons.count 0.624 0.484 0.349 0.477 0.568 17.230 0.000 0.275 0.000

Sainsbury.count 0.773 0.419 0.436 0.496 0.804 21.031 0.000 0.337 0.000

premium.count 0.545 0.498 0.257 0.437 0.578 19.127 0.000 0.288 0.000

Detached 8.871 7.762 19.383 12.577 -1.354 -26.941 0.000 0.398 0.000

SemiDetached 20.694 13.521 28.259 11.219 -0.560 -19.261 0.000 0.301 0.000

Terraced 25.750 16.191 27.121 14.817 -0.085 -2.715 0.007 0.125 0.000

Flats 44.686 24.909 25.237 16.564 0.781 31.110 0.000 0.360 0.000

Rooms1 2.151 2.656 0.807 1.142 0.506 25.109 0.000 0.318 0.000

Rooms23 25.271 12.124 16.462 8.405 0.727 28.210 0.000 0.345 0.000

Rooms45 50.520 10.059 52.777 8.402 -0.224 -7.687 0.000 0.136 0.000

Rooms6p 22.058 9.693 29.954 10.095 -0.815 -23.632 0.000 0.307 0.000

Affluent.Achievers 13.090 16.497 24.838 19.498 -0.712 -18.641 0.000 0.309 0.000

Rising.Prosperity 8.311 12.896 3.414 5.423 0.380 19.002 0.000 0.191 0.000

Comfortable.Communities 15.399 14.166 23.751 16.702 -0.590 -15.464 0.000 0.220 0.000

Financially.Stretched 13.700 12.397 14.508 12.947 -0.065 -1.887 0.059 0.069 0.000

Urban.Adversity 13.705 14.791 6.366 9.915 0.496 19.676 0.000 0.305 0.000

Not.Private.Households 6.780 5.927 5.285 4.981 0.252 8.608 0.000 0.156 0.000

Not.Found 29.014 18.568 21.838 15.674 0.386 13.142 0.000 0.216 0.000

ewLondonkm 3.807 1.496 4.380 0.991 -0.383 -15.283 0.000 0.205 0.000

nsLondonkm 3.758 1.767 4.469 1.204 -0.402 -15.769 0.000 0.225 0.000

ahah.v2 22.675 6.755 22.739 6.878 -0.009 -0.281 0.779 0.027 0.512

Lemployment 0.923 1.010 1.864 0.958 -0.932 -29.073 0.000 0.419 0.000

raildist 0.179 0.921 0.927 1.128 -0.813 -20.645 0.000 0.324 0.000

$es.mean.ATT

tx.mn tx.sd ct.mn ct.sd std.eff.sz stat p ks ks.pval

discount.count 0.560 0.496 0.513 0.500 0.095 1.316 0.188 0.047 0.772

freezer.count 0.571 0.495 0.510 0.500 0.125 1.721 0.085 0.062 0.442

Budgens.Spar.count 0.220 0.414 0.241 0.428 -0.051 -0.568 0.570 0.021 1.000

Co.op.count 0.530 0.499 0.561 0.496 -0.063 -0.916 0.360 0.031 0.989

Asda.count 0.718 0.450 0.648 0.478 0.156 1.991 0.046 0.070 0.292

Morrisons.count 0.624 0.484 0.571 0.495 0.109 1.540 0.124 0.053 0.636

Sainsbury.count 0.773 0.419 0.773 0.419 0.000 0.009 0.993 0.000 1.000

premium.count 0.545 0.498 0.576 0.494 -0.062 -0.930 0.352 0.031 0.990

Detached 8.871 7.762 8.998 7.948 -0.016 -0.250 0.803 0.058 0.516

SemiDetached 20.694 13.521 20.801 13.133 -0.008 -0.099 0.921 0.050 0.700

Terraced 25.750 16.191 26.450 15.136 -0.043 -0.585 0.558 0.065 0.375

Flats 44.686 24.909 43.750 23.766 0.038 0.405 0.686 0.053 0.630

Rooms1 2.151 2.656 2.111 2.588 0.015 0.137 0.891 0.061 0.456

Rooms23 25.271 12.124 25.604 12.706 -0.028 -0.257 0.798 0.058 0.520

Rooms45 50.520 10.059 50.103 10.556 0.041 0.418 0.676 0.058 0.517

Rooms6p 22.058 9.693 22.181 9.467 -0.013 -0.161 0.872 0.041 0.891

Affluent.Achievers 13.090 16.497 14.610 16.658 -0.092 -1.550 0.121 0.122 0.006

Rising.Prosperity 8.311 12.896 9.648 13.635 -0.104 -0.831 0.406 0.062 0.447

Comfortable.Communities 15.399 14.166 15.798 13.162 -0.028 -0.421 0.673 0.081 0.155

Financially.Stretched 13.700 12.397 13.495 11.643 0.017 0.267 0.789 0.039 0.921

Urban.Adversity 13.705 14.791 11.286 11.680 0.164 3.331 0.001 0.076 0.212

Not.Private.Households 6.780 5.927 6.902 6.024 -0.021 -0.299 0.765 0.032 0.988

Not.Found 29.014 18.568 28.262 17.474 0.041 0.605 0.545 0.046 0.790

ewLondonkm 3.807 1.496 3.745 1.399 0.042 0.452 0.651 0.065 0.385

nsLondonkm 3.758 1.767 3.970 1.424 -0.120 -1.711 0.087 0.098 0.047

ahah.v2 22.675 6.755 21.803 5.736 0.129 1.433 0.152 0.088 0.098

Lemployment 0.923 1.010 0.866 1.035 0.056 0.612 0.540 0.051 0.677

raildist 0.179 0.921 0.282 0.909 -0.112 -1.531 0.126 0.100 0.042

$es.max.ATT

tx.mn tx.sd ct.mn ct.sd std.eff.sz stat p ks ks.pval

discount.count 0.560 0.496 0.508 0.500 0.104 1.524 0.128 0.052 0.595

freezer.count 0.571 0.495 0.508 0.500 0.127 1.859 0.063 0.063 0.345

Budgens.Spar.count 0.220 0.414 0.233 0.423 -0.033 -0.407 0.684 0.014 1.000

Co.op.count 0.530 0.499 0.558 0.497 -0.056 -0.862 0.389 0.028 0.994

Asda.count 0.718 0.450 0.653 0.476 0.145 2.003 0.045 0.065 0.307

Morrisons.count 0.624 0.484 0.571 0.495 0.110 1.636 0.102 0.053 0.554

Sainsbury.count 0.773 0.419 0.772 0.420 0.003 0.052 0.959 0.001 1.000

premium.count 0.545 0.498 0.570 0.495 -0.051 -0.794 0.427 0.025 0.999

Detached 8.871 7.762 9.130 7.941 -0.033 -0.542 0.588 0.046 0.733

SemiDetached 20.694 13.521 21.131 13.100 -0.032 -0.430 0.667 0.052 0.594

Terraced 25.750 16.191 26.662 15.070 -0.056 -0.822 0.411 0.067 0.273

Flats 44.686 24.909 43.078 23.416 0.065 0.758 0.449 0.053 0.555

Rooms1 2.151 2.656 2.034 2.528 0.044 0.443 0.658 0.072 0.205

Rooms23 25.271 12.124 25.202 12.472 0.006 0.058 0.954 0.047 0.714

Rooms45 50.520 10.059 50.416 10.452 0.010 0.114 0.909 0.048 0.678

Rooms6p 22.058 9.693 22.348 9.409 -0.030 -0.405 0.685 0.038 0.900

Affluent.Achievers 13.090 16.497 14.766 16.719 -0.102 -1.778 0.075 0.122 0.003

Rising.Prosperity 8.311 12.896 9.145 13.159 -0.065 -0.572 0.567 0.047 0.712

Comfortable.Communities 15.399 14.166 15.948 13.157 -0.039 -0.615 0.538 0.090 0.059

Financially.Stretched 13.700 12.397 13.713 11.641 -0.001 -0.017 0.986 0.046 0.738

Urban.Adversity 13.705 14.791 11.412 11.792 0.155 3.205 0.001 0.070 0.234

Not.Private.Households 6.780 5.927 6.954 6.125 -0.029 -0.435 0.664 0.034 0.957

Not.Found 29.014 18.568 28.063 17.464 0.051 0.799 0.424 0.047 0.717

ewLondonkm 3.807 1.496 3.787 1.378 0.014 0.162 0.871 0.052 0.582

nsLondonkm 3.758 1.767 4.001 1.410 -0.137 -2.133 0.033 0.107 0.013

ahah.v2 22.675 6.755 21.628 5.615 0.155 1.927 0.054 0.096 0.036

Lemployment 0.923 1.010 0.892 1.025 0.031 0.367 0.714 0.040 0.864

raildist 0.179 0.921 0.296 0.904 -0.127 -1.891 0.059 0.106 0.014

$ks.mean.ATT

tx.mn tx.sd ct.mn ct.sd std.eff.sz stat p ks ks.pval

discount.count 0.560 0.496 0.506 0.500 0.107 1.600 0.110 0.053 0.534

freezer.count 0.571 0.495 0.507 0.500 0.131 1.939 0.053 0.065 0.298

Budgens.Spar.count 0.220 0.414 0.231 0.421 -0.027 -0.335 0.738 0.011 1.000

Co.op.count 0.530 0.499 0.557 0.497 -0.056 -0.863 0.388 0.028 0.994

Asda.count 0.718 0.450 0.655 0.476 0.142 2.010 0.044 0.064 0.312

Morrisons.count 0.624 0.484 0.569 0.495 0.113 1.698 0.090 0.055 0.505

Sainsbury.count 0.773 0.419 0.772 0.419 0.001 0.020 0.984 0.000 1.000

premium.count 0.545 0.498 0.568 0.496 -0.047 -0.745 0.456 0.023 1.000

Detached 8.871 7.762 9.165 7.919 -0.038 -0.630 0.529 0.045 0.739

SemiDetached 20.694 13.521 21.231 13.103 -0.040 -0.539 0.590 0.052 0.577

Terraced 25.750 16.191 26.667 15.049 -0.057 -0.849 0.396 0.067 0.252

Flats 44.686 24.909 42.937 23.330 0.070 0.844 0.399 0.054 0.512

Rooms1 2.151 2.656 2.009 2.504 0.053 0.551 0.582 0.074 0.163

Rooms23 25.271 12.124 25.101 12.397 0.014 0.147 0.883 0.043 0.779

Rooms45 50.520 10.059 50.523 10.407 0.000 -0.003 0.997 0.048 0.678

Rooms6p 22.058 9.693 22.366 9.381 -0.032 -0.440 0.660 0.038 0.895

Affluent.Achievers 13.090 16.497 14.857 16.750 -0.107 -1.887 0.059 0.123 0.002

Rising.Prosperity 8.311 12.896 9.018 13.024 -0.055 -0.499 0.618 0.044 0.755

Comfortable.Communities 15.399 14.166 15.990 13.149 -0.042 -0.675 0.500 0.093 0.041

Financially.Stretched 13.700 12.397 13.759 11.669 -0.005 -0.083 0.934 0.047 0.687

Urban.Adversity 13.705 14.791 11.405 11.793 0.156 3.244 0.001 0.069 0.229

Not.Private.Households 6.780 5.927 6.971 6.176 -0.032 -0.482 0.630 0.034 0.950

Not.Found 29.014 18.568 28.000 17.459 0.055 0.868 0.386 0.047 0.701

ewLondonkm 3.807 1.496 3.800 1.371 0.005 0.063 0.950 0.048 0.663

nsLondonkm 3.758 1.767 4.004 1.408 -0.139 -2.223 0.026 0.109 0.010

ahah.v2 22.675 6.755 21.524 5.574 0.170 2.189 0.029 0.099 0.025

Lemployment 0.923 1.010 0.898 1.022 0.024 0.295 0.768 0.037 0.916

raildist 0.179 0.921 0.296 0.903 -0.127 -1.946 0.052 0.106 0.012

$ks.max.ATT

tx.mn tx.sd ct.mn ct.sd std.eff.sz stat p ks ks.pval

discount.count 0.560 0.496 0.513 0.500 0.095 1.314 0.189 0.047 0.772

freezer.count 0.571 0.495 0.510 0.500 0.125 1.718 0.086 0.062 0.443

Budgens.Spar.count 0.220 0.414 0.241 0.428 -0.052 -0.573 0.567 0.021 1.000

Co.op.count 0.530 0.499 0.561 0.496 -0.064 -0.925 0.355 0.032 0.987

Asda.count 0.718 0.450 0.648 0.478 0.156 1.993 0.046 0.070 0.289

Morrisons.count 0.624 0.484 0.571 0.495 0.109 1.530 0.126 0.053 0.644

Sainsbury.count 0.773 0.419 0.773 0.419 -0.001 -0.010 0.992 0.000 1.000

premium.count 0.545 0.498 0.576 0.494 -0.063 -0.946 0.344 0.031 0.988

Detached 8.871 7.762 8.998 7.953 -0.016 -0.248 0.804 0.059 0.510

SemiDetached 20.694 13.521 20.779 13.121 -0.006 -0.079 0.937 0.050 0.701

Terraced 25.750 16.191 26.452 15.142 -0.043 -0.586 0.558 0.065 0.378

Flats 44.686 24.909 43.771 23.767 0.037 0.395 0.693 0.053 0.630

Rooms1 2.151 2.656 2.113 2.590 0.014 0.132 0.895 0.061 0.455

Rooms23 25.271 12.124 25.610 12.711 -0.028 -0.260 0.795 0.058 0.514

Rooms45 50.520 10.059 50.091 10.557 0.043 0.430 0.667 0.059 0.510

Rooms6p 22.058 9.693 22.186 9.470 -0.013 -0.166 0.868 0.041 0.888

Affluent.Achievers 13.090 16.497 14.618 16.671 -0.093 -1.556 0.120 0.122 0.006

Rising.Prosperity 8.311 12.896 9.662 13.648 -0.105 -0.838 0.402 0.062 0.438

Comfortable.Communities 15.399 14.166 15.786 13.159 -0.027 -0.408 0.683 0.081 0.159

Financially.Stretched 13.700 12.397 13.494 11.648 0.017 0.268 0.789 0.039 0.927

Urban.Adversity 13.705 14.791 11.279 11.675 0.164 3.339 0.001 0.076 0.209

Not.Private.Households 6.780 5.927 6.899 6.025 -0.020 -0.292 0.770 0.032 0.987

Not.Found 29.014 18.568 28.262 17.479 0.041 0.604 0.546 0.046 0.790

ewLondonkm 3.807 1.496 3.743 1.399 0.043 0.461 0.645 0.065 0.377

nsLondonkm 3.758 1.767 3.969 1.424 -0.119 -1.702 0.089 0.098 0.047

ahah.v2 22.675 6.755 21.808 5.740 0.128 1.422 0.155 0.088 0.099

Lemployment 0.923 1.010 0.865 1.035 0.057 0.626 0.532 0.052 0.672

raildist 0.179 0.921 0.282 0.909 -0.111 -1.521 0.128 0.099 0.043

[1] "Table S9 : Premium"

$unw

tx.mn tx.sd ct.mn ct.sd std.eff.sz stat p ks ks.pval

discount.count 0.569 0.495 0.427 0.495 0.286 10.340 0.000 0.142 0.479

freezer.count 0.602 0.490 0.402 0.490 0.408 14.734 0.000 0.200 0.479

Budgens.Spar.count 0.243 0.429 0.180 0.384 0.147 5.582 0.000 0.063 0.479

Co.op.count 0.568 0.495 0.463 0.499 0.214 7.697 0.000 0.106 0.479

Asda.count 0.703 0.457 0.588 0.492 0.252 8.781 0.000 0.115 0.479

Morrisons.count 0.658 0.475 0.476 0.500 0.383 13.487 0.000 0.182 0.479

Sainsbury.count 0.861 0.346 0.547 0.498 0.909 26.637 0.000 0.314 0.479

Tesco.count 0.883 0.322 0.684 0.465 0.615 18.015 0.000 0.198 0.479

Detached 7.695 7.322 14.423 11.079 -0.919 -26.063 0.000 0.283 0.479

SemiDetached 17.420 12.854 26.941 12.259 -0.741 -27.348 0.000 0.332 0.479

Terraced 22.541 15.455 29.308 15.629 -0.438 -15.723 0.000 0.207 0.479

Flats 52.344 24.852 29.328 18.615 0.926 37.650 0.000 0.407 0.479

Rooms1 2.653 2.938 1.115 1.617 0.524 23.197 0.000 0.341 0.479

Rooms23 28.228 12.381 18.790 9.569 0.762 30.651 0.000 0.345 0.479

Rooms45 48.098 9.789 53.724 8.923 -0.575 -21.647 0.000 0.229 0.479

Rooms6p 21.021 10.275 26.372 9.664 -0.521 -19.349 0.000 0.232 0.479

Affluent.Achievers 13.740 17.251 17.475 18.258 -0.216 -7.599 0.000 0.127 0.479

Rising.Prosperity 11.433 14.865 3.337 5.824 0.545 25.543 0.000 0.338 0.479

Comfortable.Communities 12.286 13.059 21.832 15.527 -0.731 -24.103 0.000 0.299 0.479

Financially.Stretched 10.973 10.992 16.572 13.237 -0.509 -16.675 0.000 0.218 0.479

Urban.Adversity 12.560 14.332 11.652 14.053 0.063 2.308 0.021 0.087 0.479

Not.Private.Households 7.270 6.150 5.691 5.272 0.257 9.921 0.000 0.138 0.479

Not.Found 31.739 18.935 23.441 16.548 0.438 16.806 0.000 0.225 0.479

ewLondonkm 3.536 1.614 4.302 1.091 -0.475 -19.944 0.000 0.228 0.479

nsLondonkm 3.461 1.829 4.335 1.416 -0.478 -19.197 0.000 0.247 0.479

ahah.v2 23.258 7.311 22.161 6.206 0.150 5.819 0.000 0.068 0.479

Lemployment 0.697 1.027 1.531 0.951 -0.812 -30.378 0.000 0.364 0.479

raildist 0.006 0.860 0.657 1.054 -0.758 -24.555 0.000 0.294 0.479

$es.mean.ATT

tx.mn tx.sd ct.mn ct.sd std.eff.sz stat p ks ks.pval

discount.count 0.569 0.495 0.574 0.495 -0.011 -0.270 0.787 0.006 1.000

freezer.count 0.602 0.490 0.577 0.494 0.052 1.220 0.223 0.025 0.859

Budgens.Spar.count 0.243 0.429 0.203 0.402 0.094 2.305 0.021 0.040 0.316

Co.op.count 0.568 0.495 0.516 0.500 0.106 2.490 0.013 0.052 0.090

Asda.count 0.703 0.457 0.758 0.428 -0.121 -3.307 0.001 0.055 0.064

Morrisons.count 0.658 0.475 0.633 0.482 0.052 1.256 0.209 0.025 0.874

Sainsbury.count 0.861 0.346 0.825 0.380 0.105 2.948 0.003 0.036 0.437

Tesco.count 0.883 0.322 0.862 0.345 0.063 1.547 0.122 0.020 0.972

Detached 7.695 7.322 9.107 7.575 -0.193 -5.055 0.000 0.120 0.000

SemiDetached 17.420 12.854 19.922 12.124 -0.195 -4.867 0.000 0.117 0.000

Terraced 22.541 15.455 24.449 15.293 -0.123 -2.884 0.004 0.077 0.002

Flats 52.344 24.852 46.521 21.967 0.234 5.359 0.000 0.115 0.000

Rooms1 2.653 2.938 2.246 2.632 0.139 2.764 0.006 0.097 0.000

Rooms23 28.228 12.381 26.352 11.158 0.151 3.573 0.000 0.078 0.002

Rooms45 48.098 9.789 49.329 9.287 -0.126 -3.122 0.002 0.056 0.055

Rooms6p 21.021 10.275 22.072 9.559 -0.102 -2.461 0.014 0.070 0.008

Affluent.Achievers 13.740 17.251 14.627 18.171 -0.051 -1.068 0.285 0.040 0.329

Rising.Prosperity 11.433 14.865 7.304 10.493 0.278 6.880 0.000 0.129 0.000

Comfortable.Communities 12.286 13.059 14.944 13.315 -0.204 -4.994 0.000 0.127 0.000

Financially.Stretched 10.973 10.992 12.429 11.242 -0.132 -3.532 0.000 0.099 0.000

Urban.Adversity 12.560 14.332 13.404 14.235 -0.059 -1.512 0.131 0.058 0.045

Not.Private.Households 7.270 6.150 7.373 6.705 -0.017 -0.348 0.728 0.025 0.876

Not.Found 31.739 18.935 29.918 18.327 0.096 2.114 0.035 0.058 0.042

ewLondonkm 3.536 1.614 3.771 1.391 -0.145 -3.440 0.001 0.092 0.000

nsLondonkm 3.461 1.829 3.770 1.630 -0.169 -4.383 0.000 0.095 0.000

ahah.v2 23.258 7.311 22.026 5.930 0.169 4.690 0.000 0.082 0.001

Lemployment 0.697 1.027 0.879 0.935 -0.177 -4.594 0.000 0.086 0.000

raildist 0.006 0.860 0.142 0.871 -0.159 -3.965 0.000 0.087 0.000

$es.max.ATT

tx.mn tx.sd ct.mn ct.sd std.eff.sz stat p ks ks.pval

discount.count 0.569 0.495 0.580 0.494 -0.022 -0.517 0.605 0.011 1.000

freezer.count 0.602 0.490 0.574 0.495 0.057 1.290 0.197 0.028 0.803

Budgens.Spar.count 0.243 0.429 0.211 0.408 0.075 1.679 0.093 0.032 0.648

Co.op.count 0.568 0.495 0.526 0.499 0.086 1.959 0.050 0.043 0.296

Asda.count 0.703 0.457 0.744 0.436 -0.090 -2.261 0.024 0.041 0.338

Morrisons.count 0.658 0.475 0.639 0.480 0.038 0.890 0.374 0.018 0.994

Sainsbury.count 0.861 0.346 0.824 0.381 0.107 2.863 0.004 0.037 0.470

Tesco.count 0.883 0.322 0.858 0.350 0.078 1.695 0.090 0.025 0.897

Detached 7.695 7.322 9.067 7.462 -0.187 -4.798 0.000 0.122 0.000

SemiDetached 17.420 12.854 20.392 12.272 -0.231 -5.428 0.000 0.123 0.000

Terraced 22.541 15.455 24.739 15.142 -0.142 -3.220 0.001 0.090 0.000

Flats 52.344 24.852 45.802 21.855 0.263 5.639 0.000 0.129 0.000

Rooms1 2.653 2.938 2.172 2.535 0.164 3.153 0.002 0.105 0.000

Rooms23 28.228 12.381 25.809 10.890 0.195 4.497 0.000 0.096 0.000

Rooms45 48.098 9.789 49.541 9.156 -0.147 -3.544 0.000 0.068 0.016

Rooms6p 21.021 10.275 22.477 9.652 -0.142 -3.200 0.001 0.087 0.001

Affluent.Achievers 13.740 17.251 14.785 17.784 -0.061 -1.331 0.183 0.060 0.046

Rising.Prosperity 11.433 14.865 7.507 10.823 0.264 5.854 0.000 0.128 0.000

Comfortable.Communities 12.286 13.059 15.230 13.288 -0.225 -5.290 0.000 0.142 0.000

Financially.Stretched 10.973 10.992 12.371 11.037 -0.127 -3.325 0.001 0.102 0.000

Urban.Adversity 12.560 14.332 13.048 13.952 -0.034 -0.873 0.383 0.057 0.070

Not.Private.Households 7.270 6.150 7.159 6.185 0.018 0.395 0.693 0.025 0.895

Not.Found 31.739 18.935 29.901 18.419 0.097 2.018 0.044 0.064 0.030

ewLondonkm 3.536 1.614 3.779 1.386 -0.150 -3.502 0.000 0.090 0.000

nsLondonkm 3.461 1.829 3.751 1.603 -0.158 -4.077 0.000 0.094 0.000

ahah.v2 23.258 7.311 21.786 5.731 0.201 5.745 0.000 0.101 0.000

Lemployment 0.697 1.027 0.887 0.948 -0.184 -4.511 0.000 0.090 0.000

raildist 0.006 0.860 0.165 0.849 -0.185 -4.607 0.000 0.102 0.000

$ks.mean.ATT

tx.mn tx.sd ct.mn ct.sd std.eff.sz stat p ks ks.pval

discount.count 0.569 0.495 0.573 0.495 -0.007 -0.180 0.857 0.004 1.000

freezer.count 0.602 0.490 0.576 0.494 0.053 1.292 0.196 0.026 0.811

Budgens.Spar.count 0.243 0.429 0.199 0.399 0.103 2.611 0.009 0.044 0.200

Co.op.count 0.568 0.495 0.510 0.500 0.118 2.846 0.004 0.058 0.035

Asda.count 0.703 0.457 0.761 0.426 -0.128 -3.620 0.000 0.058 0.035

Morrisons.count 0.658 0.475 0.628 0.483 0.063 1.547 0.122 0.030 0.661

Sainsbury.count 0.861 0.346 0.823 0.381 0.110 3.137 0.002 0.038 0.351

Tesco.count 0.883 0.322 0.863 0.344 0.060 1.543 0.123 0.019 0.979

Detached 7.695 7.322 9.129 7.626 -0.196 -5.226 0.000 0.122 0.000

SemiDetached 17.420 12.854 19.851 12.077 -0.189 -4.879 0.000 0.118 0.000

Terraced 22.541 15.455 24.558 15.306 -0.131 -3.134 0.002 0.080 0.001

Flats 52.344 24.852 46.462 21.940 0.237 5.597 0.000 0.117 0.000

Rooms1 2.653 2.938 2.263 2.643 0.133 2.740 0.006 0.093 0.000

Rooms23 28.228 12.381 26.461 11.215 0.143 3.426 0.001 0.073 0.003

Rooms45 48.098 9.789 49.344 9.352 -0.127 -3.207 0.001 0.054 0.062

Rooms6p 21.021 10.275 21.932 9.489 -0.089 -2.211 0.027 0.068 0.008

Affluent.Achievers 13.740 17.251 14.329 18.096 -0.034 -0.736 0.462 0.030 0.641

Rising.Prosperity 11.433 14.865 7.229 10.449 0.283 7.186 0.000 0.128 0.000

Comfortable.Communities 12.286 13.059 14.920 13.343 -0.202 -5.074 0.000 0.124 0.000

Financially.Stretched 10.973 10.992 12.592 11.402 -0.147 -3.937 0.000 0.101 0.000

Urban.Adversity 12.560 14.332 13.700 14.442 -0.080 -2.041 0.041 0.065 0.013

Not.Private.Households 7.270 6.150 7.435 6.909 -0.027 -0.546 0.585 0.026 0.825

Not.Found 31.739 18.935 29.795 18.278 0.103 2.327 0.020 0.059 0.033

ewLondonkm 3.536 1.614 3.770 1.384 -0.145 -3.542 0.000 0.095 0.000

nsLondonkm 3.461 1.829 3.768 1.639 -0.167 -4.405 0.000 0.094 0.000

ahah.v2 23.258 7.311 22.187 5.996 0.146 4.088 0.000 0.073 0.004

Lemployment 0.697 1.027 0.883 0.929 -0.180 -4.815 0.000 0.089 0.000

raildist 0.006 0.860 0.137 0.880 -0.152 -3.830 0.000 0.083 0.001

$ks.max.ATT

tx.mn tx.sd ct.mn ct.sd std.eff.sz stat p ks ks.pval

discount.count 0.569 0.495 0.567 0.496 0.003 0.073 0.942 0.001 1.000

freezer.count 0.602 0.490 0.572 0.495 0.062 1.566 0.117 0.031 0.593

Budgens.Spar.count 0.243 0.429 0.195 0.396 0.112 2.981 0.003 0.048 0.106

Co.op.count 0.568 0.495 0.504 0.500 0.130 3.217 0.001 0.064 0.011

Asda.count 0.703 0.457 0.762 0.426 -0.130 -3.789 0.000 0.059 0.024

Morrisons.count 0.658 0.475 0.622 0.485 0.075 1.896 0.058 0.036 0.400

Sainsbury.count 0.861 0.346 0.817 0.387 0.130 3.716 0.000 0.045 0.158

Tesco.count 0.883 0.322 0.862 0.345 0.065 1.766 0.077 0.021 0.940

Detached 7.695 7.322 9.192 7.729 -0.204 -5.630 0.000 0.125 0.000

SemiDetached 17.420 12.854 19.802 12.026 -0.185 -4.965 0.000 0.119 0.000

Terraced 22.541 15.455 24.586 15.269 -0.132 -3.310 0.001 0.082 0.000

Flats 52.344 24.852 46.420 21.958 0.238 5.894 0.000 0.116 0.000

Rooms1 2.653 2.938 2.267 2.645 0.131 2.831 0.005 0.090 0.000

Rooms23 28.228 12.381 26.586 11.244 0.133 3.304 0.001 0.067 0.007

Rooms45 48.098 9.789 49.418 9.382 -0.135 -3.486 0.000 0.056 0.039

Rooms6p 21.021 10.275 21.729 9.381 -0.069 -1.810 0.070 0.066 0.009

Affluent.Achievers 13.740 17.251 14.140 18.042 -0.023 -0.521 0.602 0.023 0.886

Rising.Prosperity 11.433 14.865 7.143 10.372 0.289 7.600 0.000 0.127 0.000

Comfortable.Communities 12.286 13.059 14.961 13.349 -0.205 -5.330 0.000 0.125 0.000

Financially.Stretched 10.973 10.992 12.804 11.558 -0.167 -4.483 0.000 0.105 0.000

Urban.Adversity 12.560 14.332 14.028 14.617 -0.102 -2.650 0.008 0.076 0.001

Not.Private.Households 7.270 6.150 7.447 7.073 -0.029 -0.580 0.562 0.028 0.690

Not.Found 31.739 18.935 29.478 18.164 0.119 2.827 0.005 0.063 0.013

ewLondonkm 3.536 1.614 3.782 1.383 -0.152 -3.830 0.000 0.096 0.000

nsLondonkm 3.461 1.829 3.769 1.651 -0.168 -4.476 0.000 0.093 0.000

ahah.v2 23.258 7.311 22.300 6.037 0.131 3.722 0.000 0.065 0.010

Lemployment 0.697 1.027 0.896 0.929 -0.194 -5.311 0.000 0.093 0.000

raildist 0.006 0.860 0.138 0.892 -0.154 -3.946 0.000 0.081 0.001
