# Supplementary material for: Identifying the effect of retail brands on private residential rental prices in Great Britain
Source: J Hous Built Environ. 2021 Oct 5;37(3):1489–509. doi: 10.1007/s10901-021-09904-2 (PMC8491747; doi:10.1007/s10901-021-09904-2)

Key to Supplementary Plots S1 to S9

Each of these plots are provided, as labelled, for the four stopping criteria: es.mean, es.max, ks.mean, and ks.max.

Top left: Convergence plots

These plots show how quickly the Gradient Boosted Random Forest algorithm to estimate the balance score improves as the number of iteration increases.

Top right: Overlapping propensity score distributions

This plot shows how well the distribution of the propensity score for the treatment group (the top box plot) and the weighted control group (the bottom box plot) overlap. Ideally there should be at least some degree of overlap in these distributions, so that effective matching can take place.

Middle left: Reduction is the absolution standardised differences

Each line and pair of circles represents a co-variate used in the GBRF models to estimate the propensity score. The line connects the absolute standardised difference between the treatment and the control group before weighting and the same difference after weighting. Ideally this latter value should be less than 0.2. (the detail on this plot is provided in Supplementary Tables S1 to S9)

Middle Right: Check on the distribution of p values for a t-test

After weighting we would expect the effect size p-values to be larger if balance had been achieved. These QQ plot compare the quantiles of the observed p-values to the quantiles of the uniform distribution (45 degree line). Ideally, the p-values from independent tests in which the null hypothesis is true will have a uniform distribution. Severe deviation of the p-values below the diagonal suggests lack of balance and p-values running at or above the diagonal suggests balance might have been achieved.

Bottom left: Check on the distribution of p values for a ks-test

A similar interpretation to that for the t-test described above. If the hollow points line on or above the 45 degree line then there is good evidence for significant balance.

Bottom right: Distribution of weights

Ideally these weight distributions should be compact and small in magnitude. Large weights suggest that some control observations are making a dis-proportionate contribution to estimating the outcome.

Figure S1 : Discounter

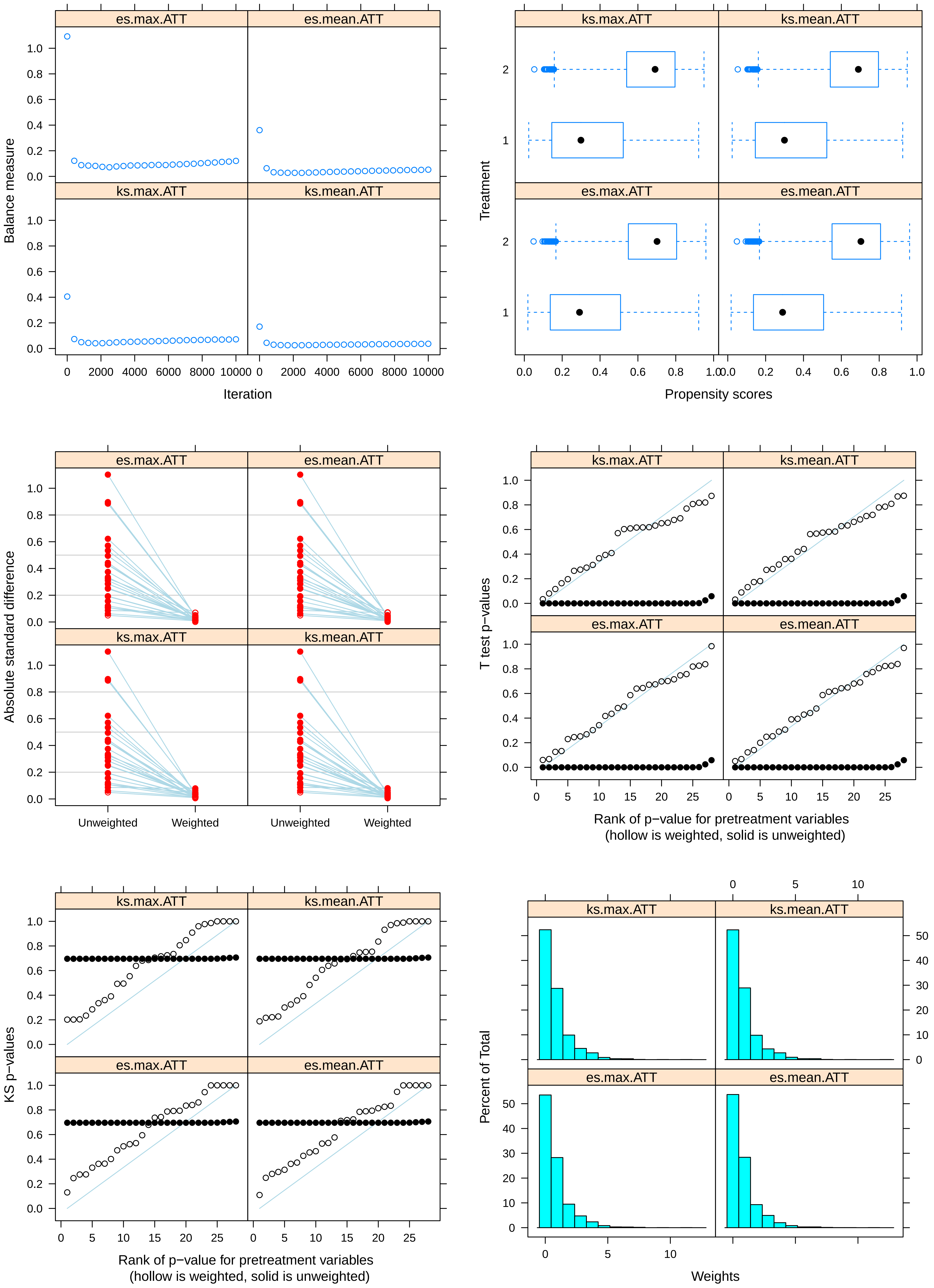

Figure S2 : Freezer

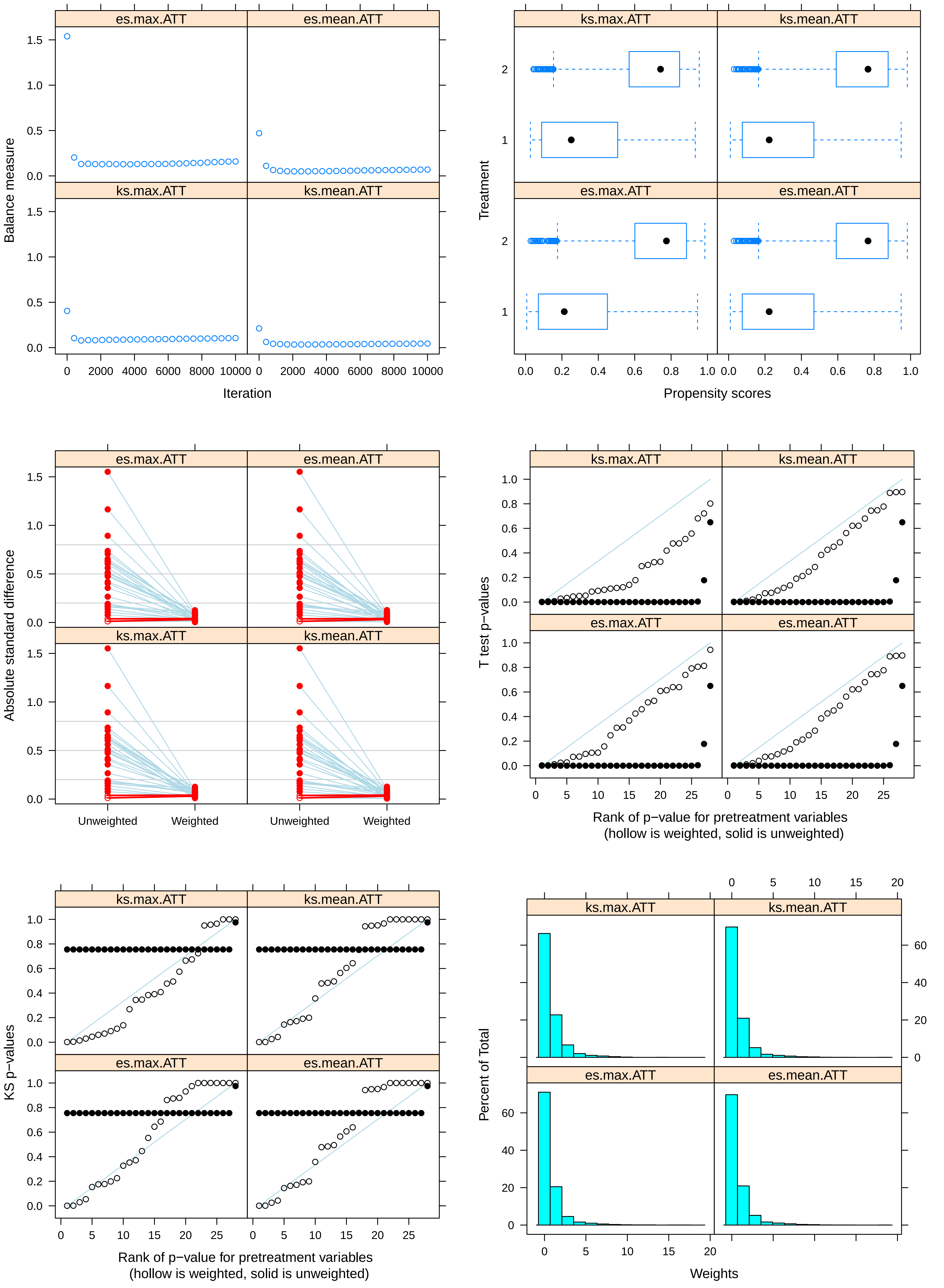

Figure S3 : Budgen/Spar

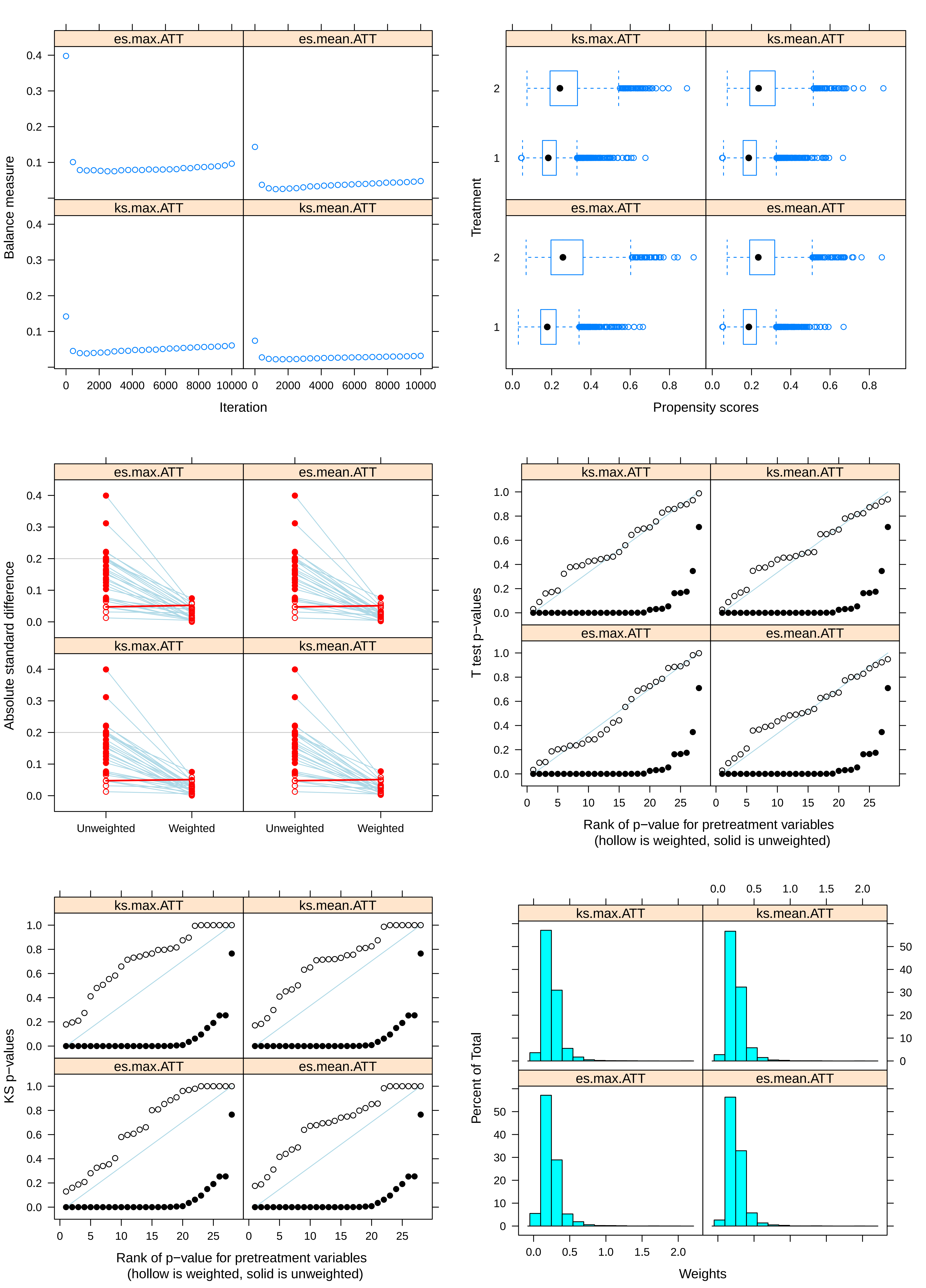

Figure S4 : Co−op

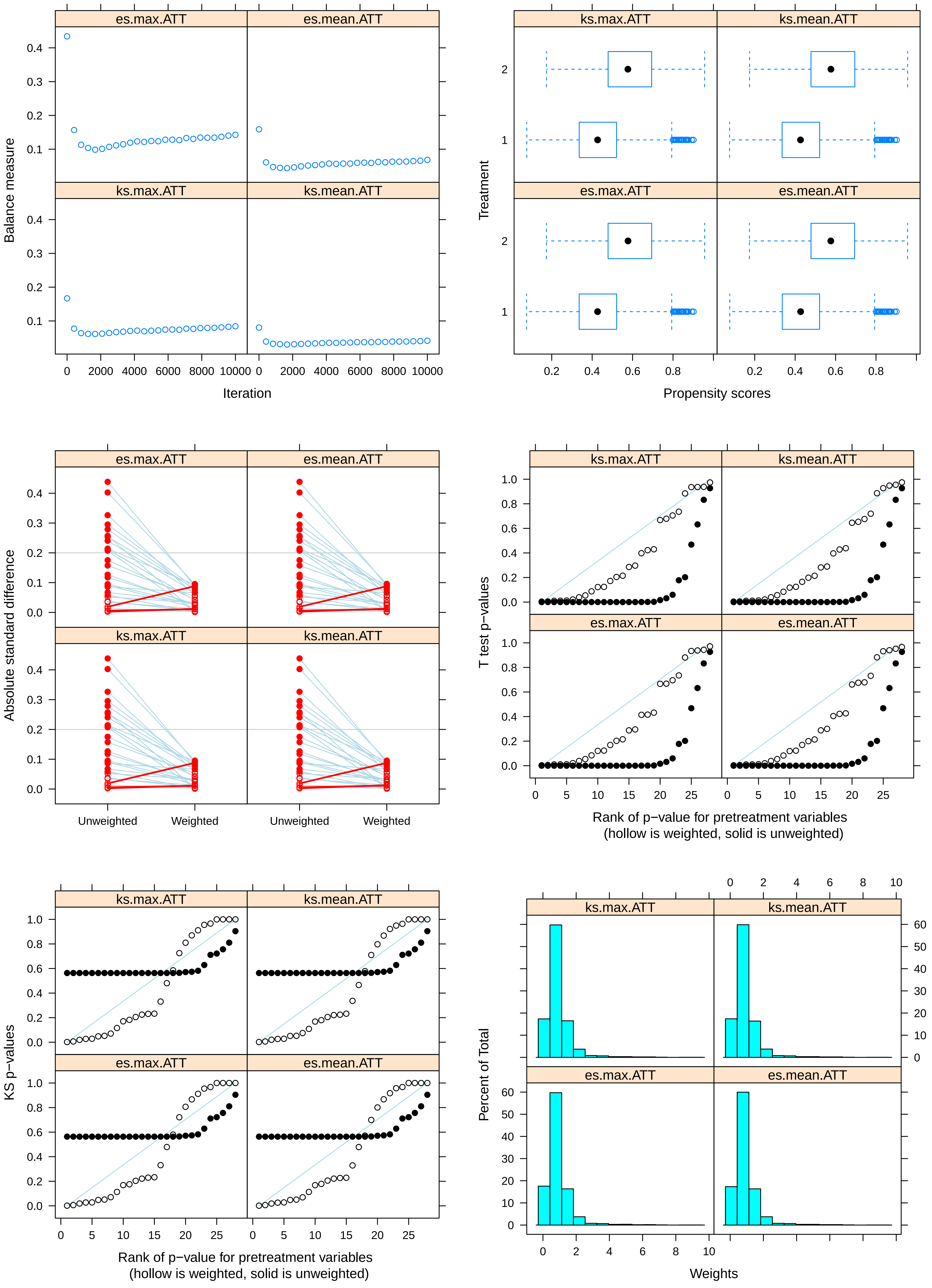

Figure S5 : ASDA

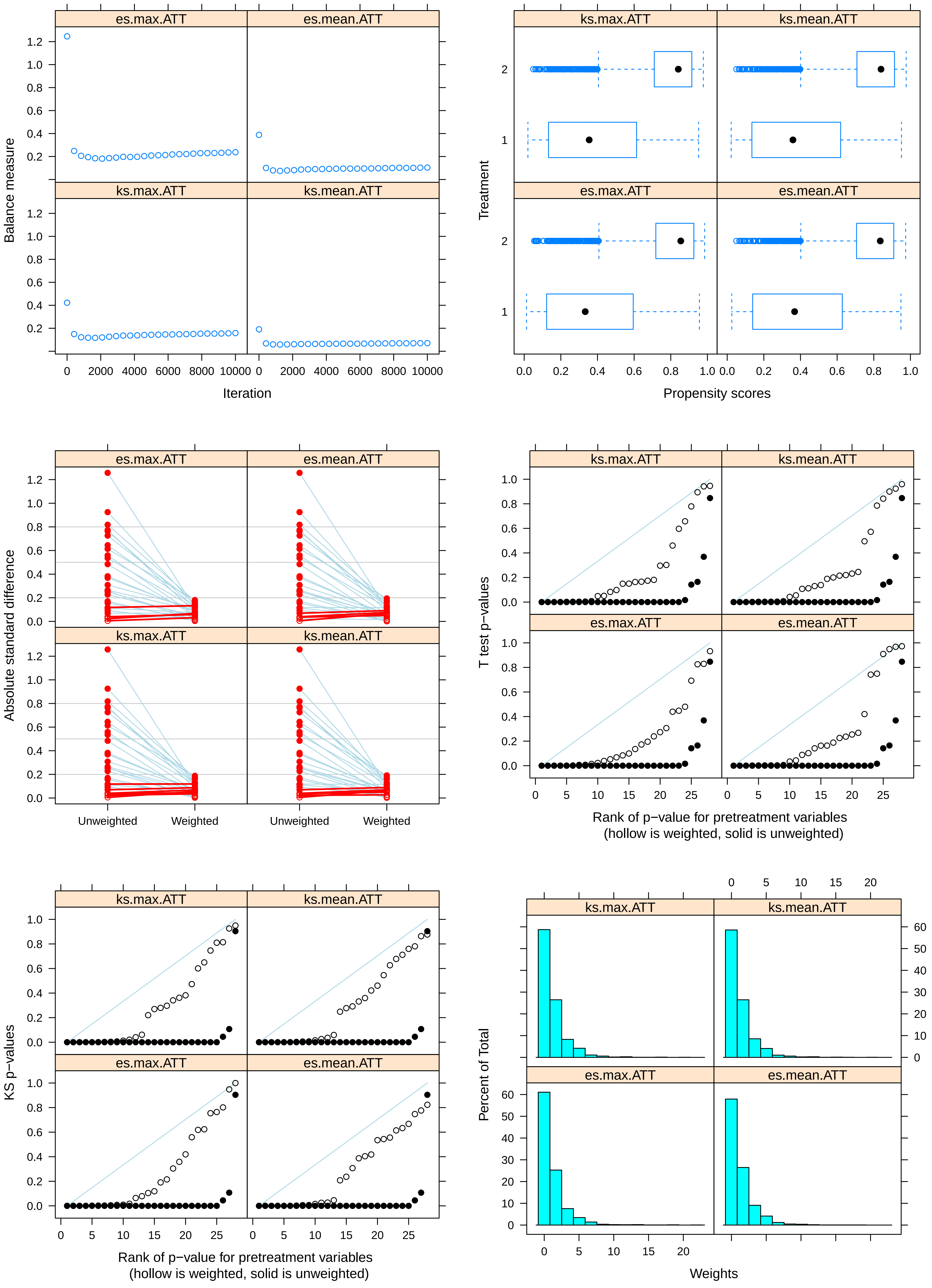

Figure S6 : Morrisons

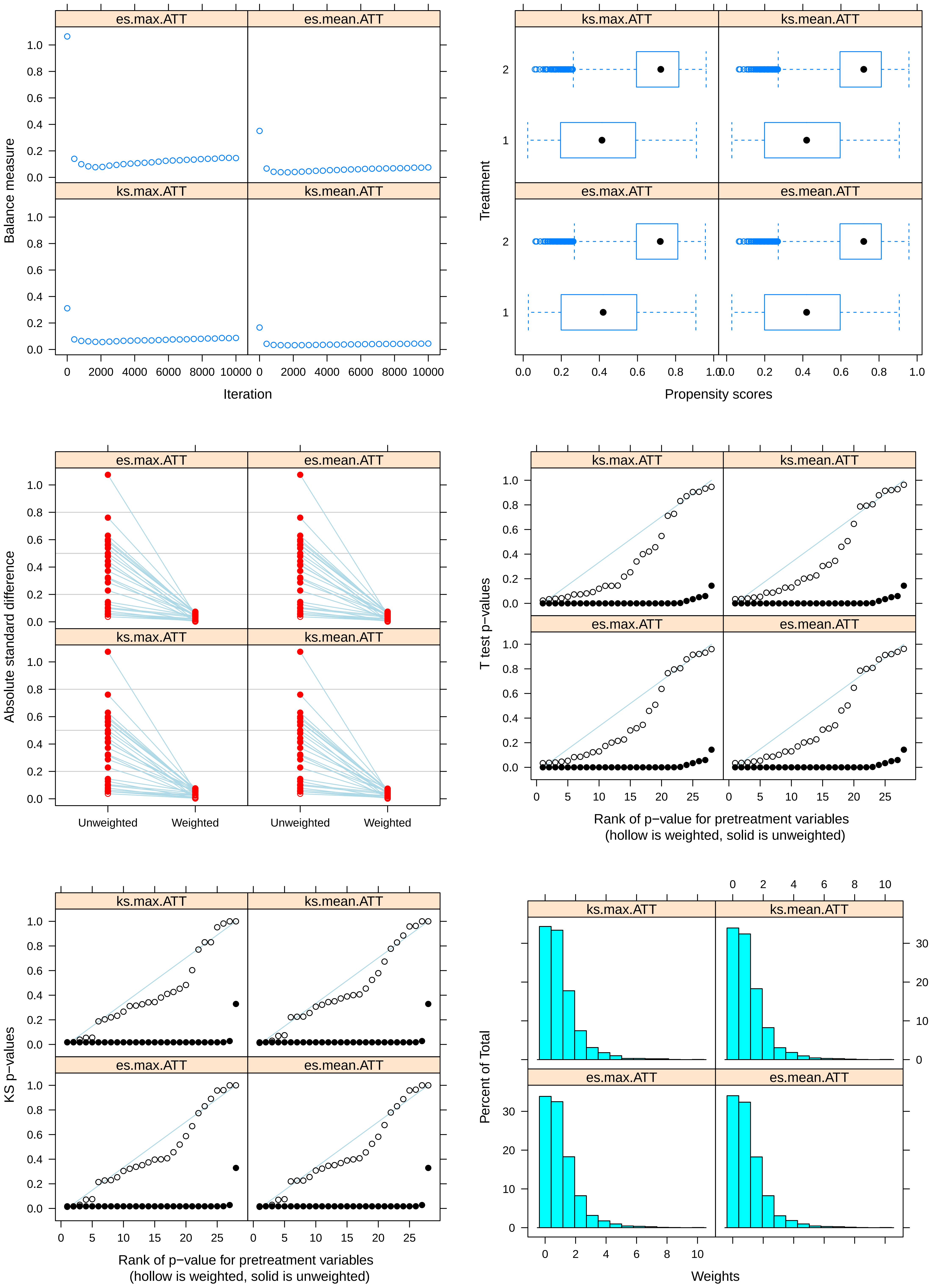

Figure S7 : Sainsbury's

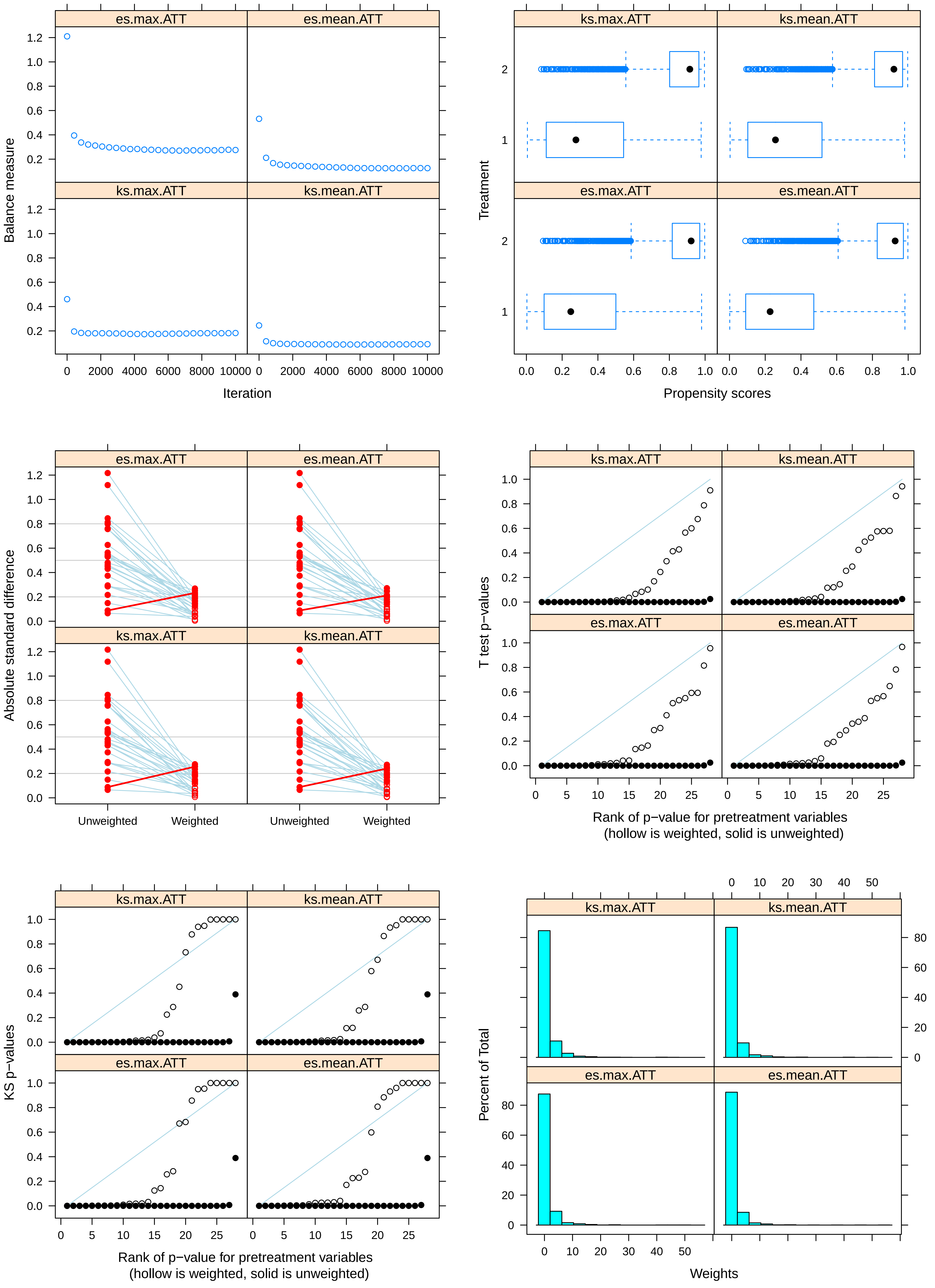

Figure S8 : Tesco

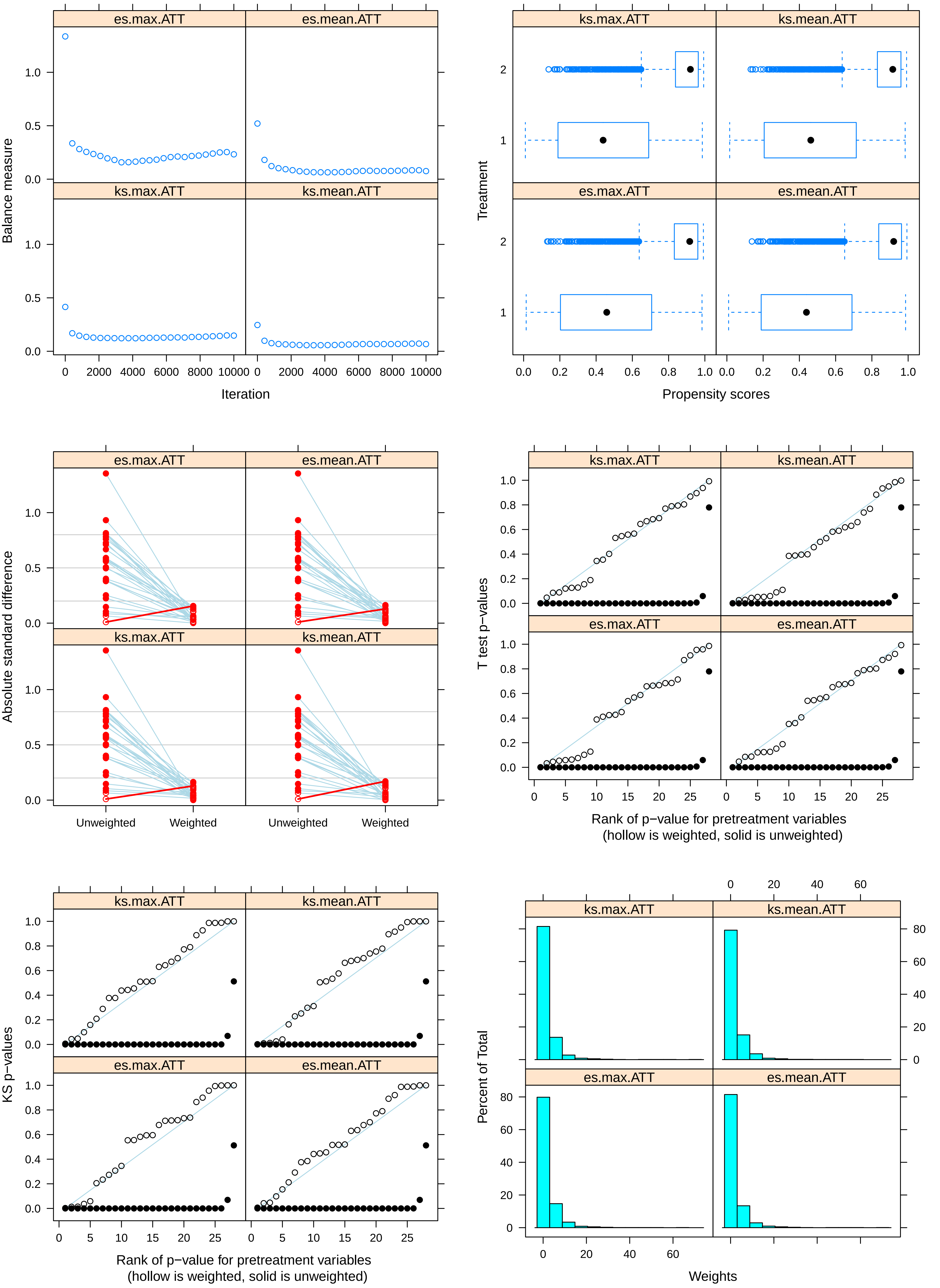

Figure S9 : Premium

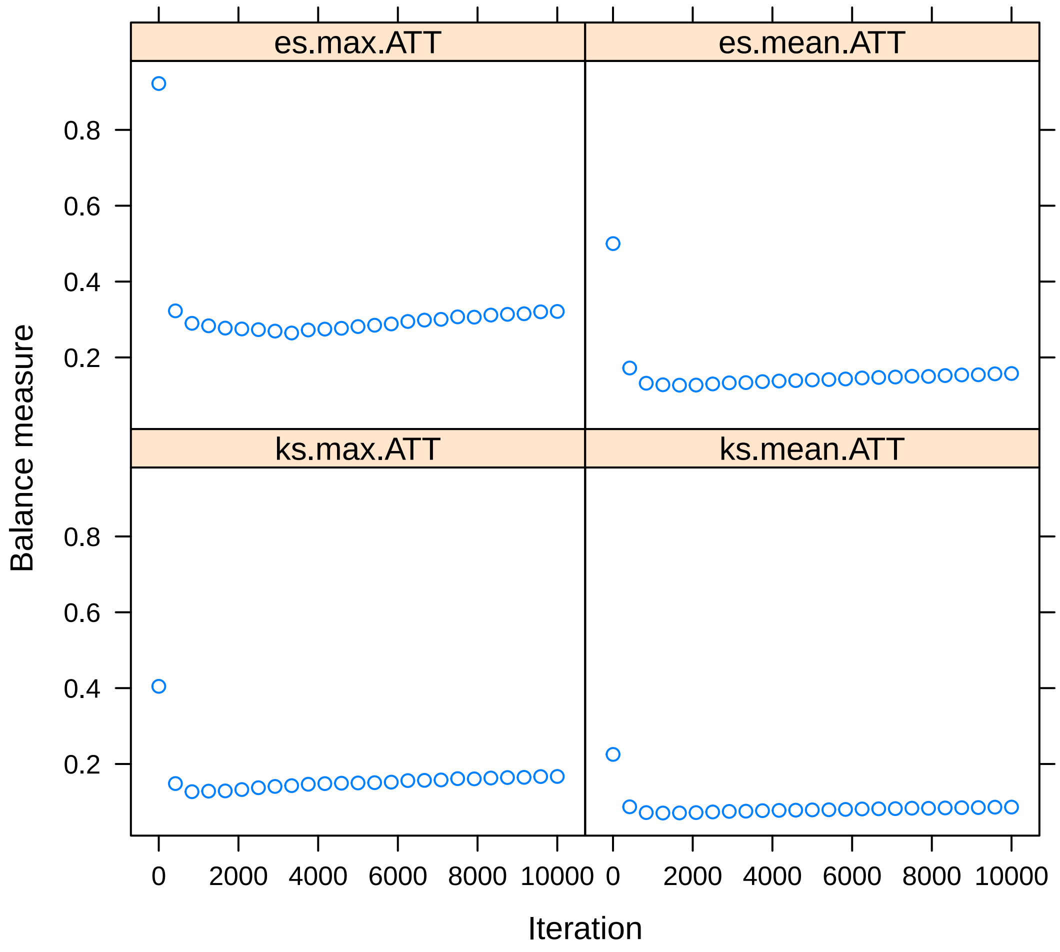

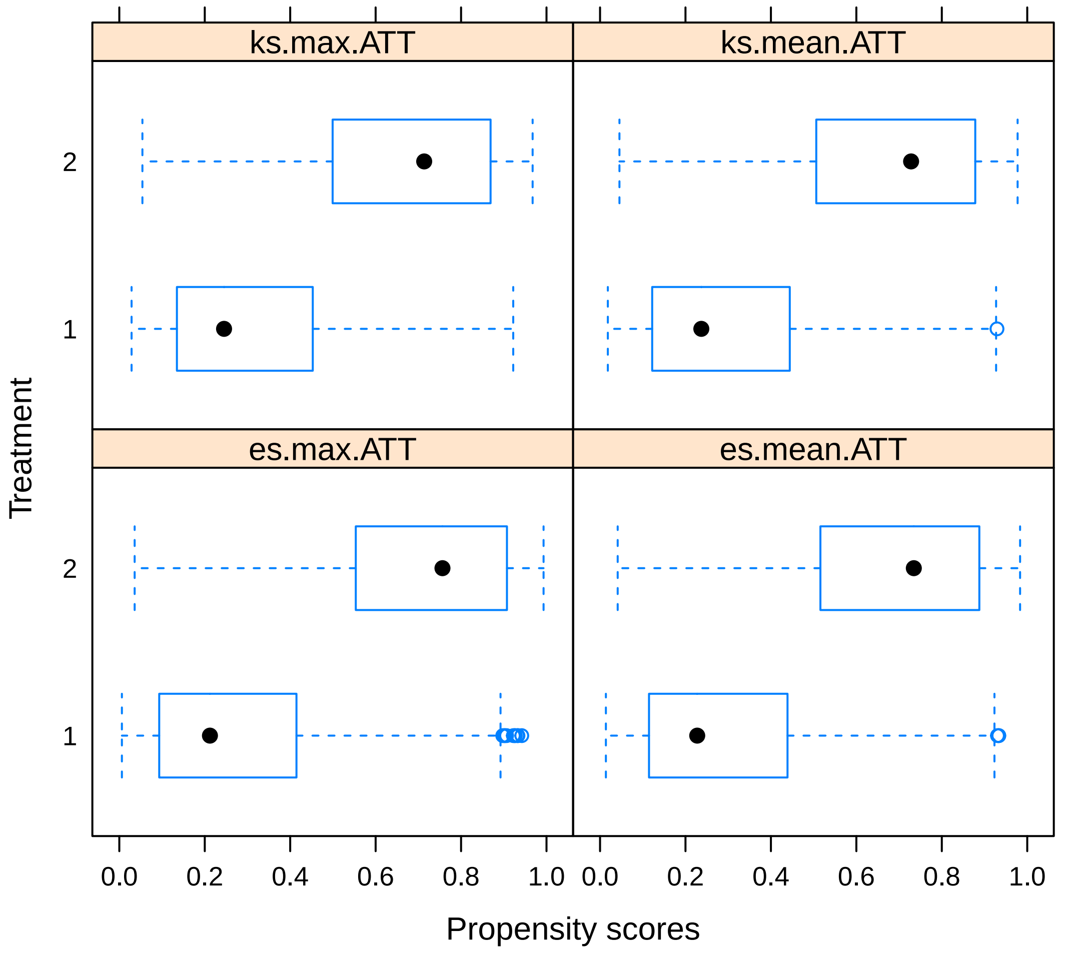

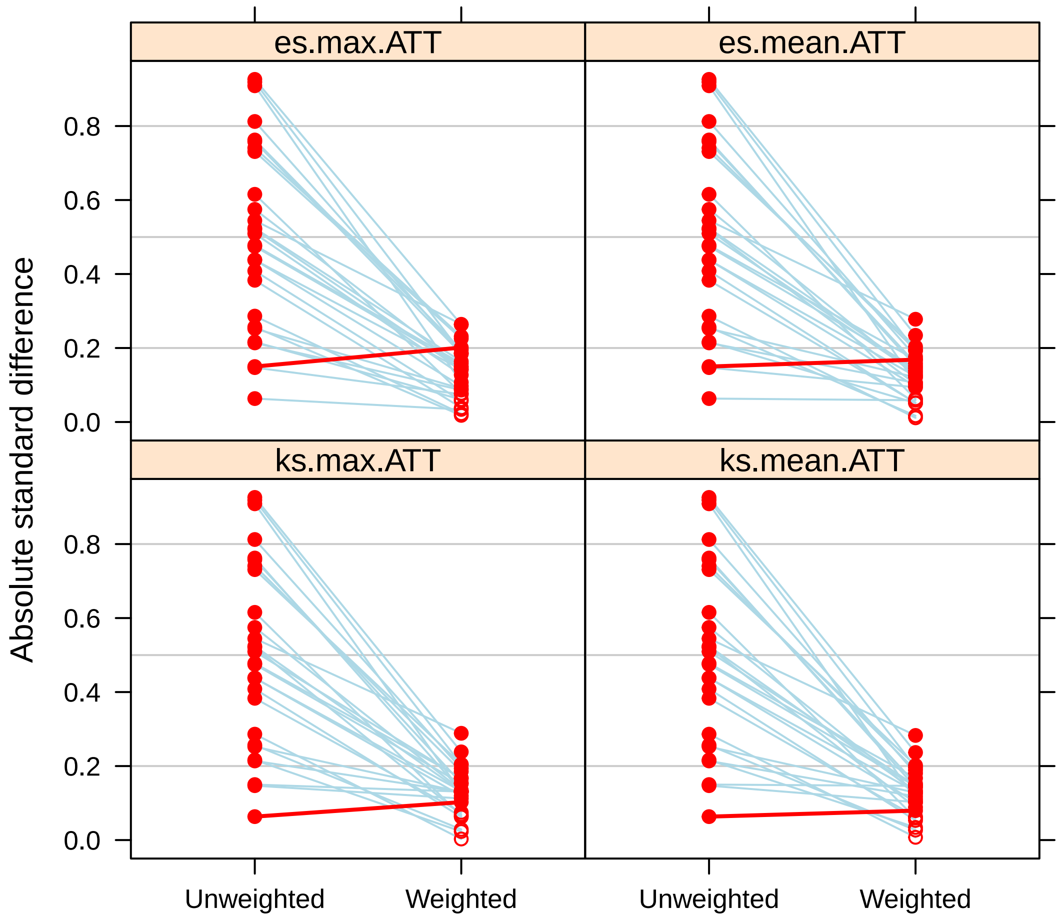

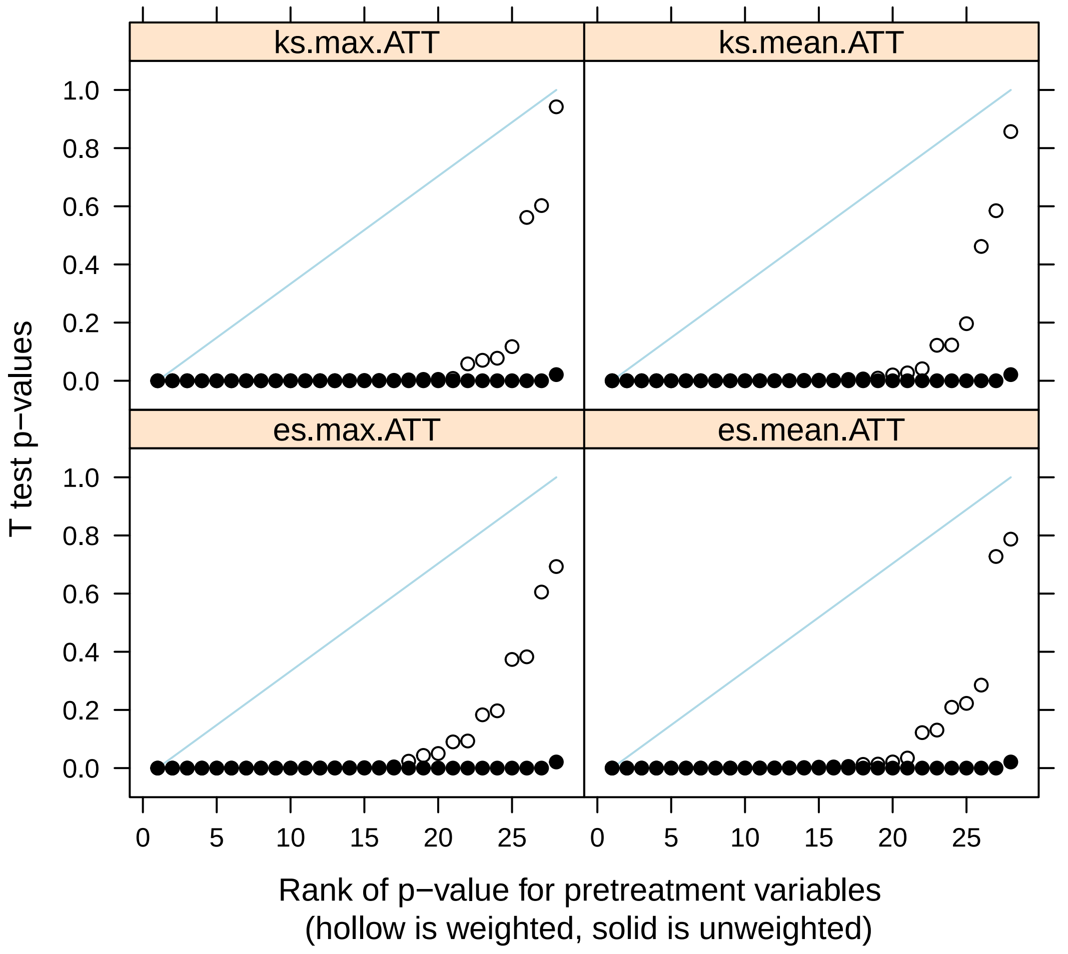

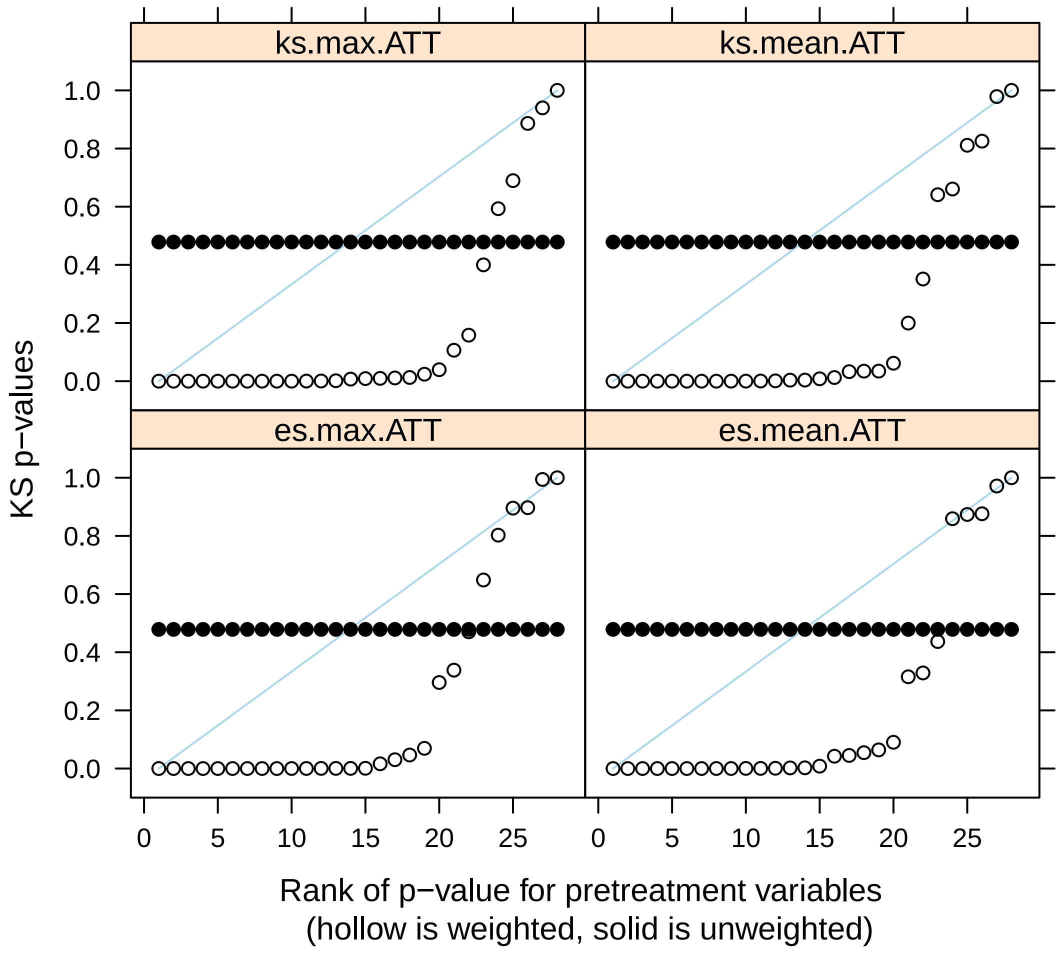

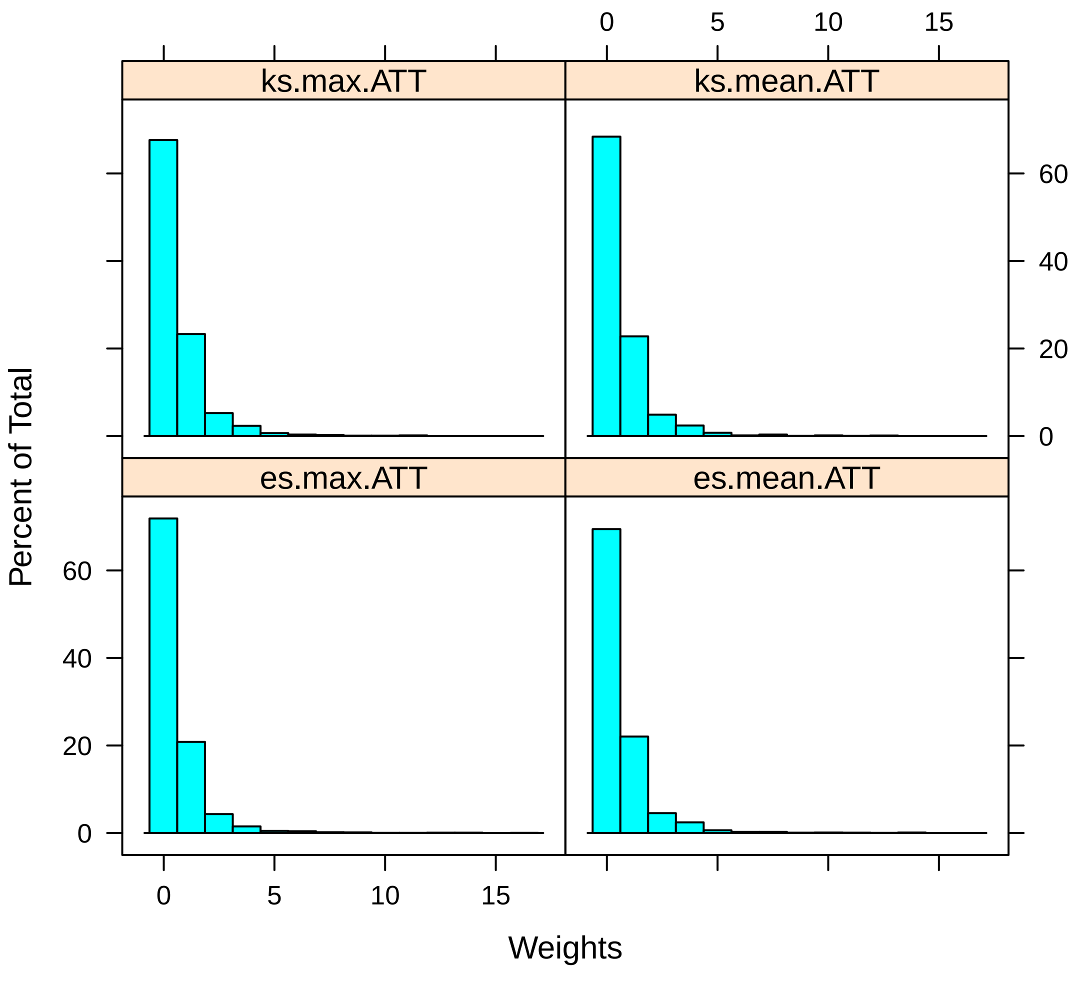

Supplement: Supplementary file 2 — Supplementary file2 (PDF 1017 KB) [file 10901_2021_9904_MOESM2_ESM.pdf]
