# Supplementary figures and images for: Identifying the effect of retail brands on private residential rental prices in Great Britain
Source: J Hous Built Environ. 2021 Oct 5;37(3):1489–509. doi: 10.1007/s10901-021-09904-2 (PMC8491747; doi:10.1007/s10901-021-09904-2)

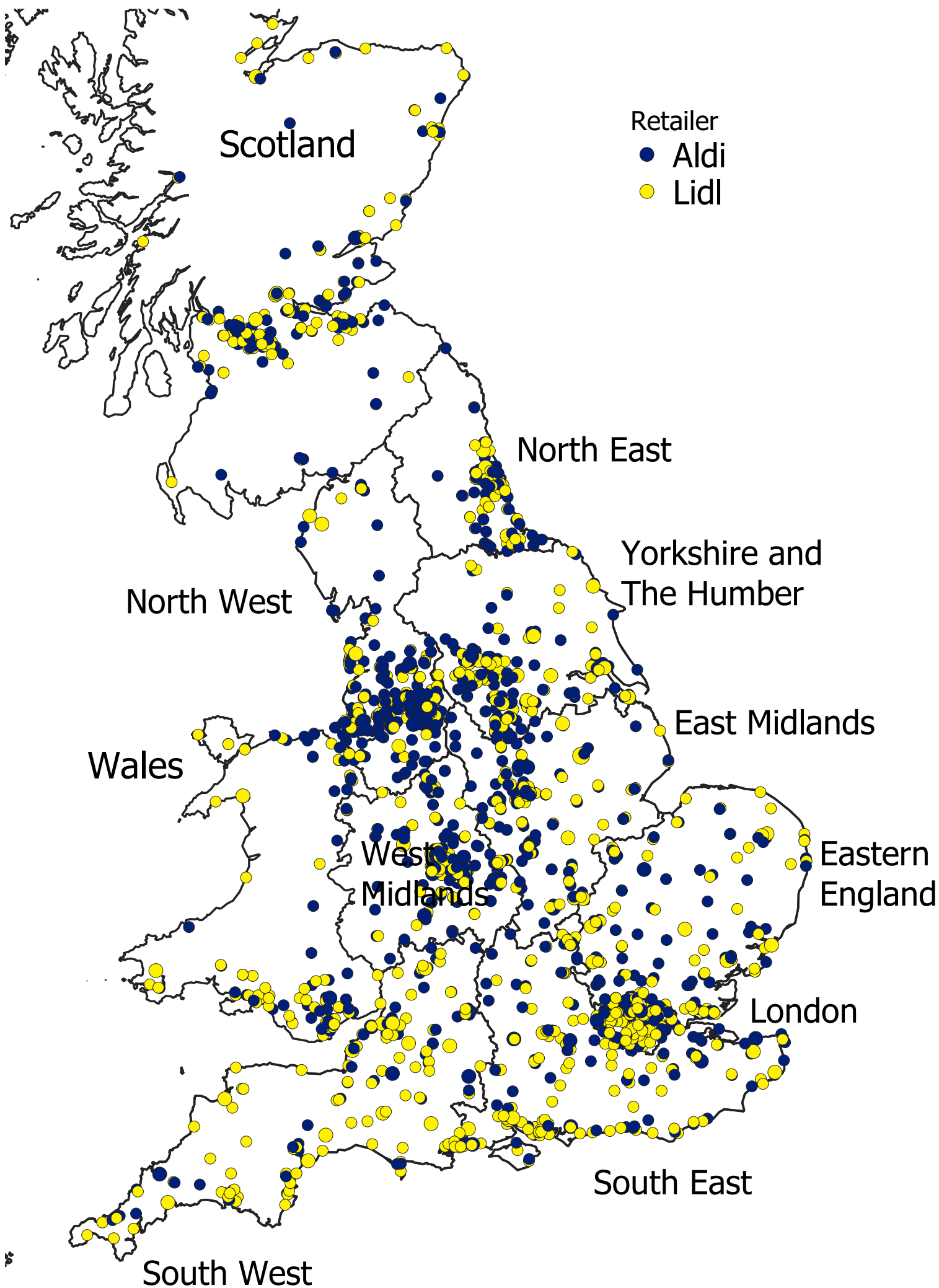

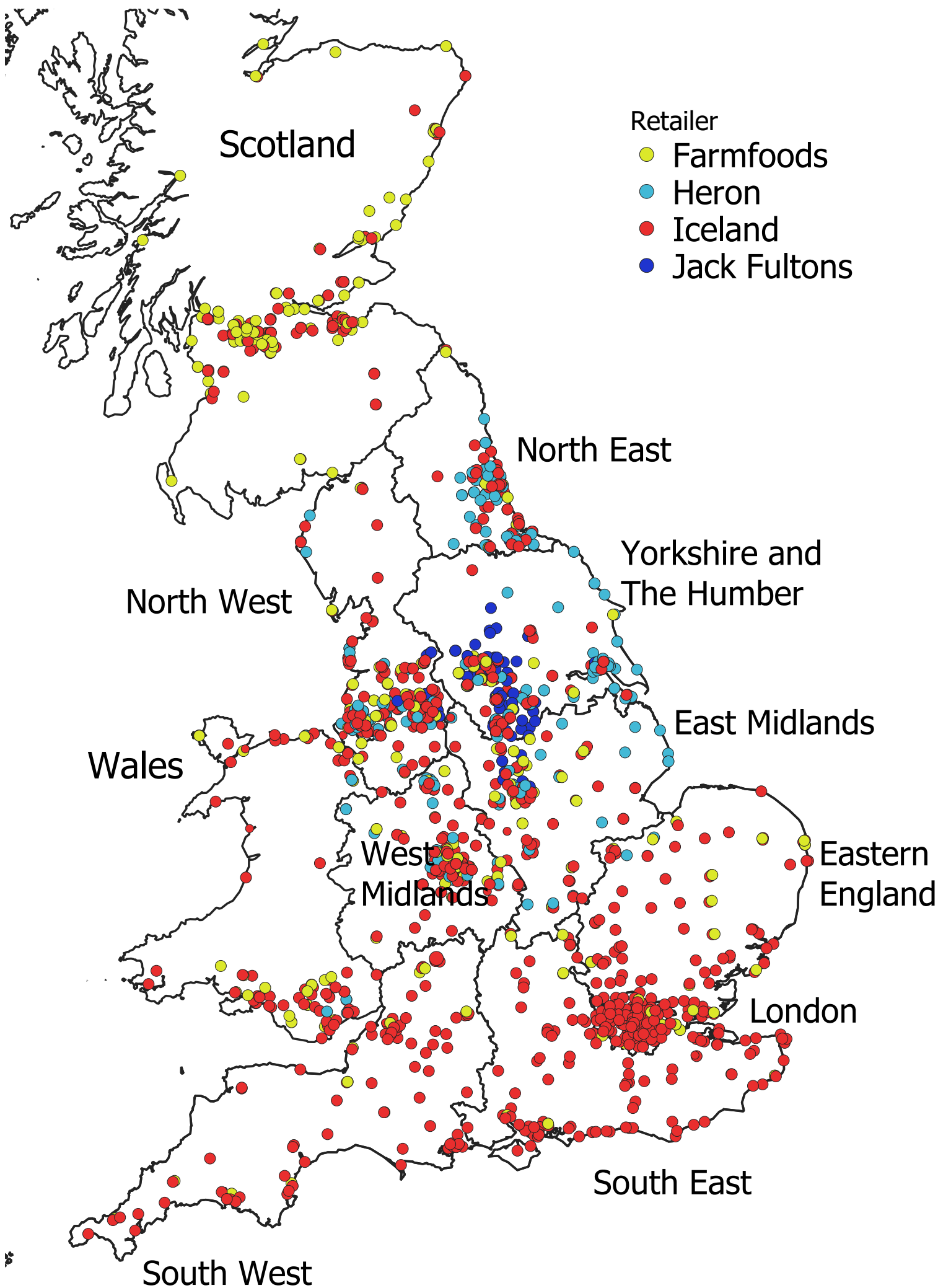

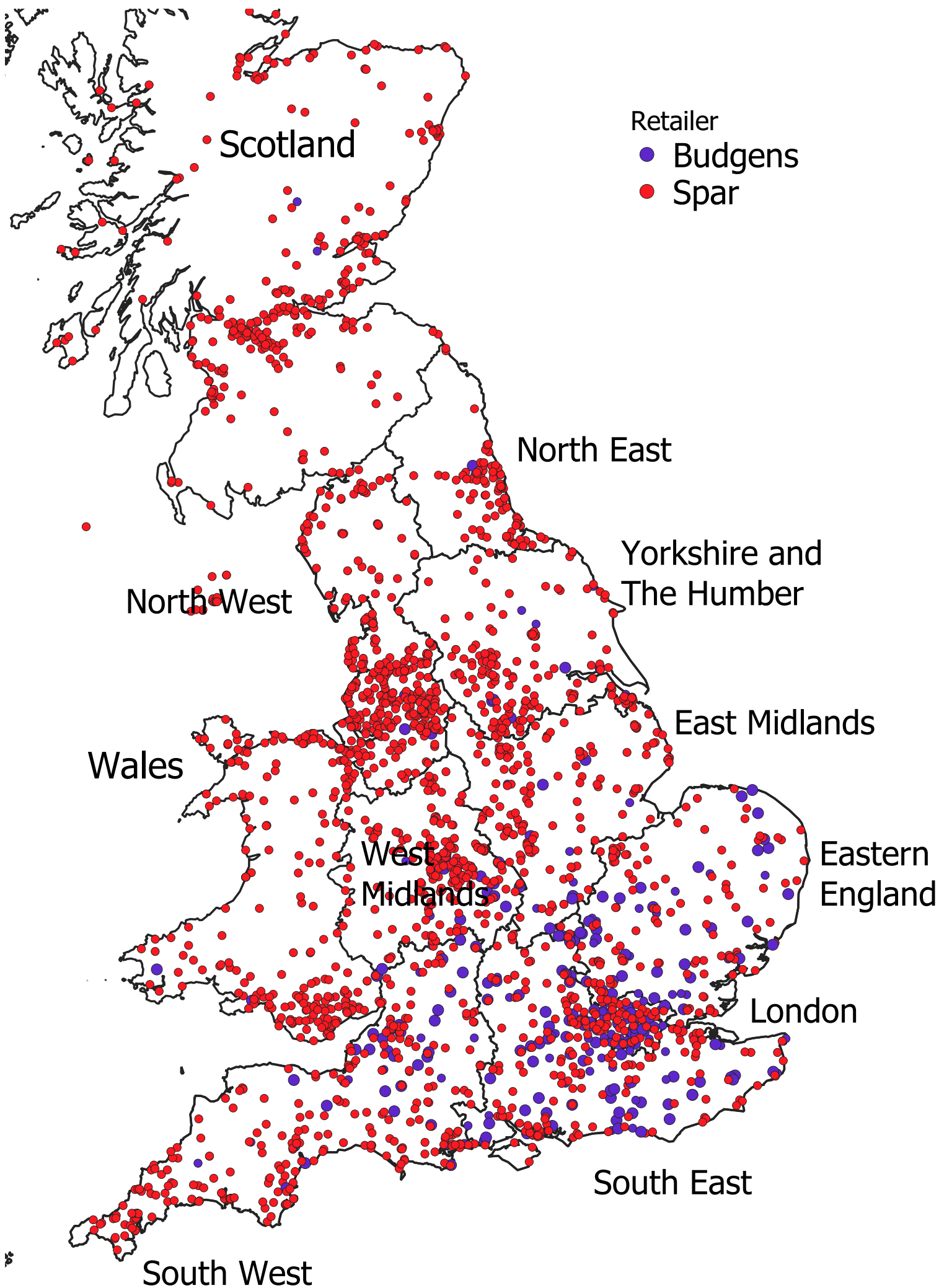

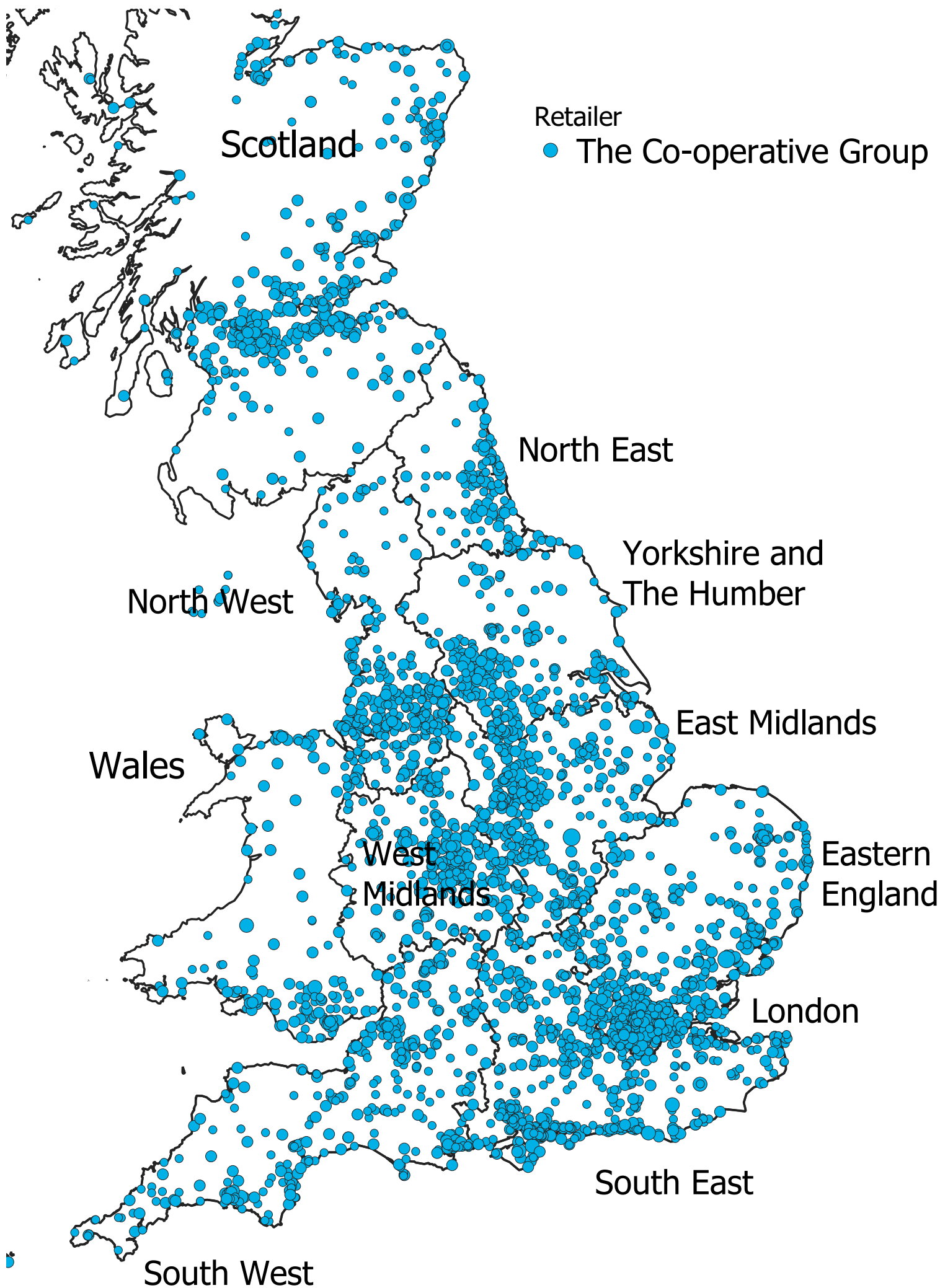

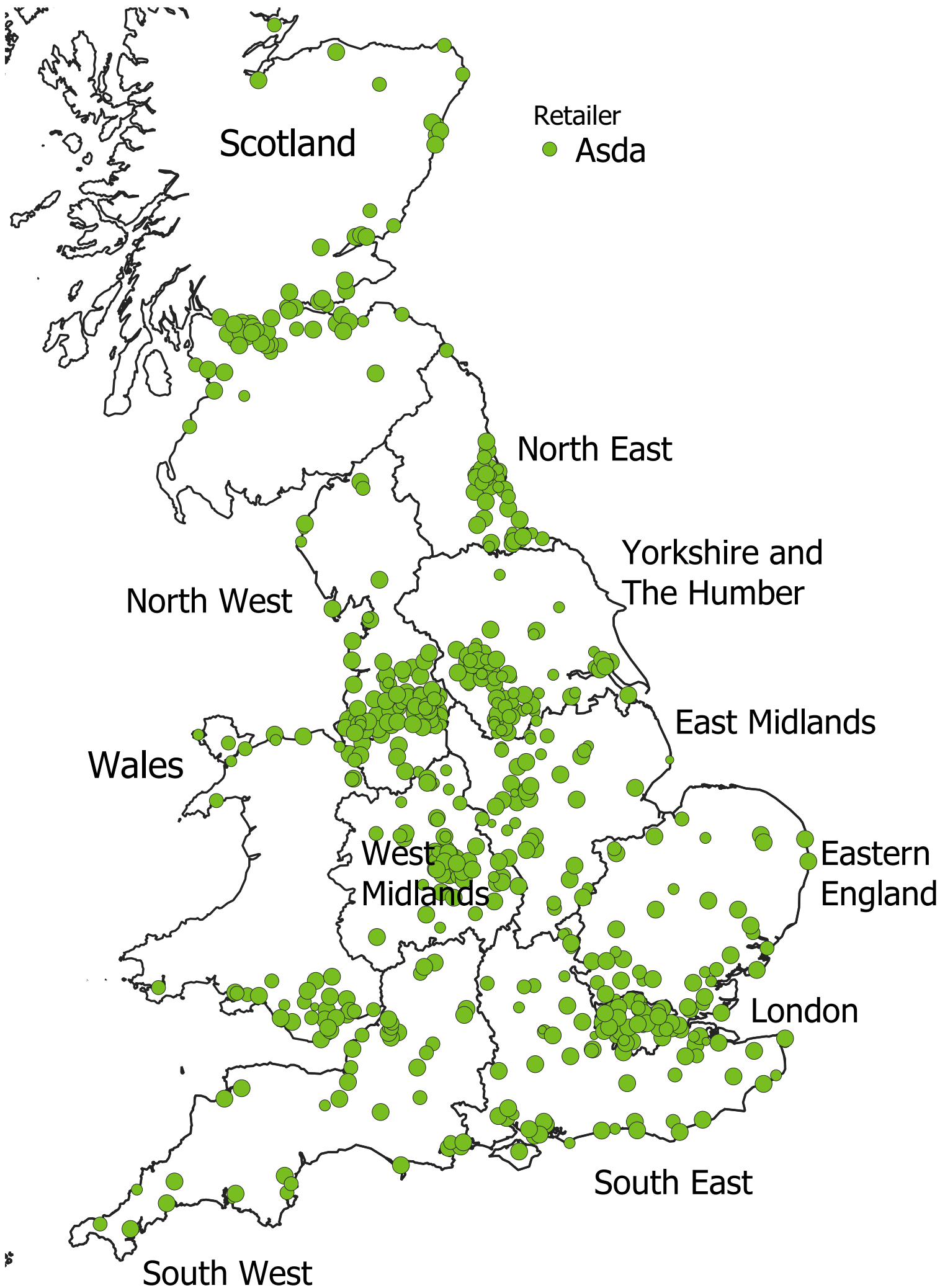

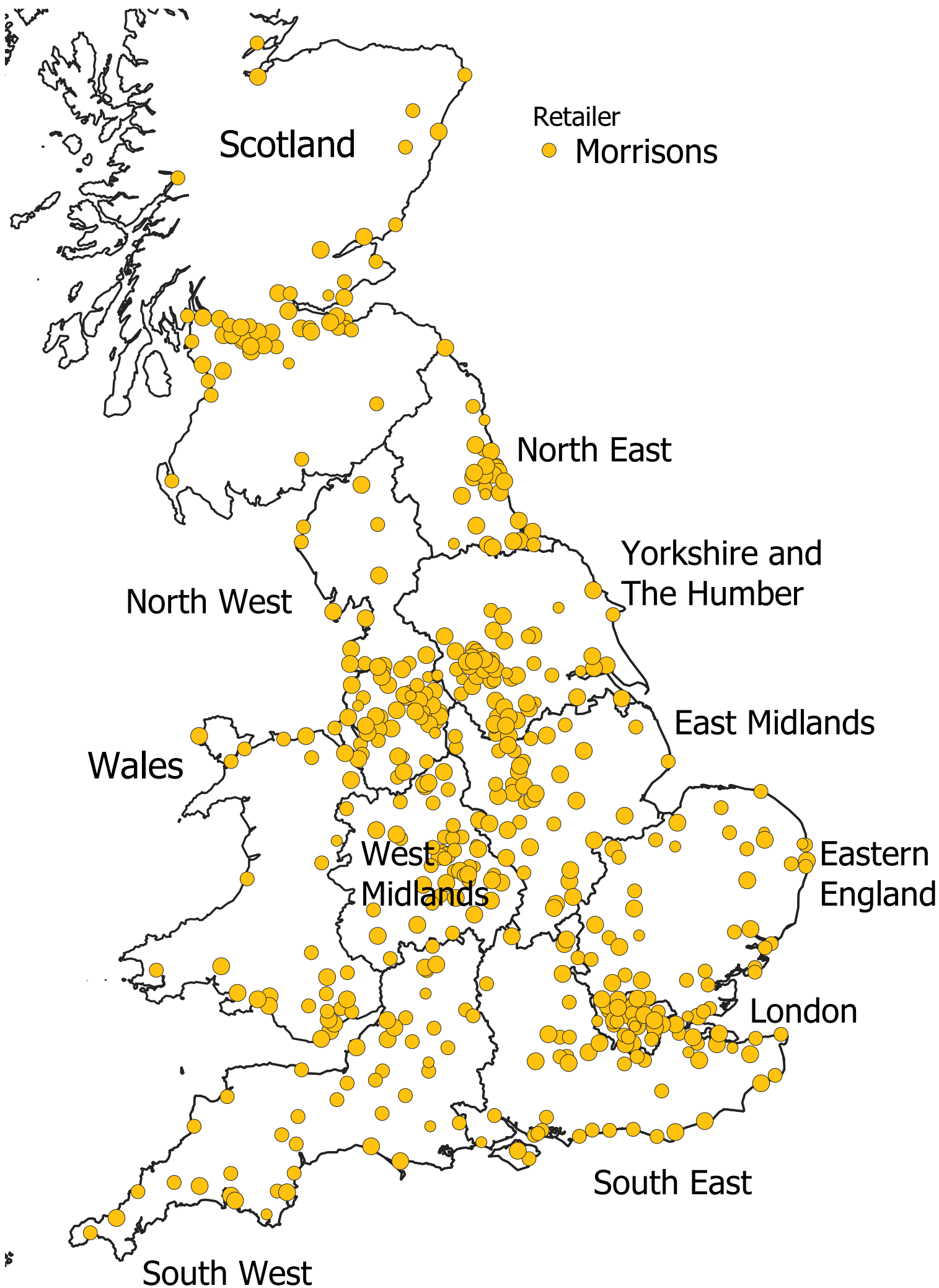

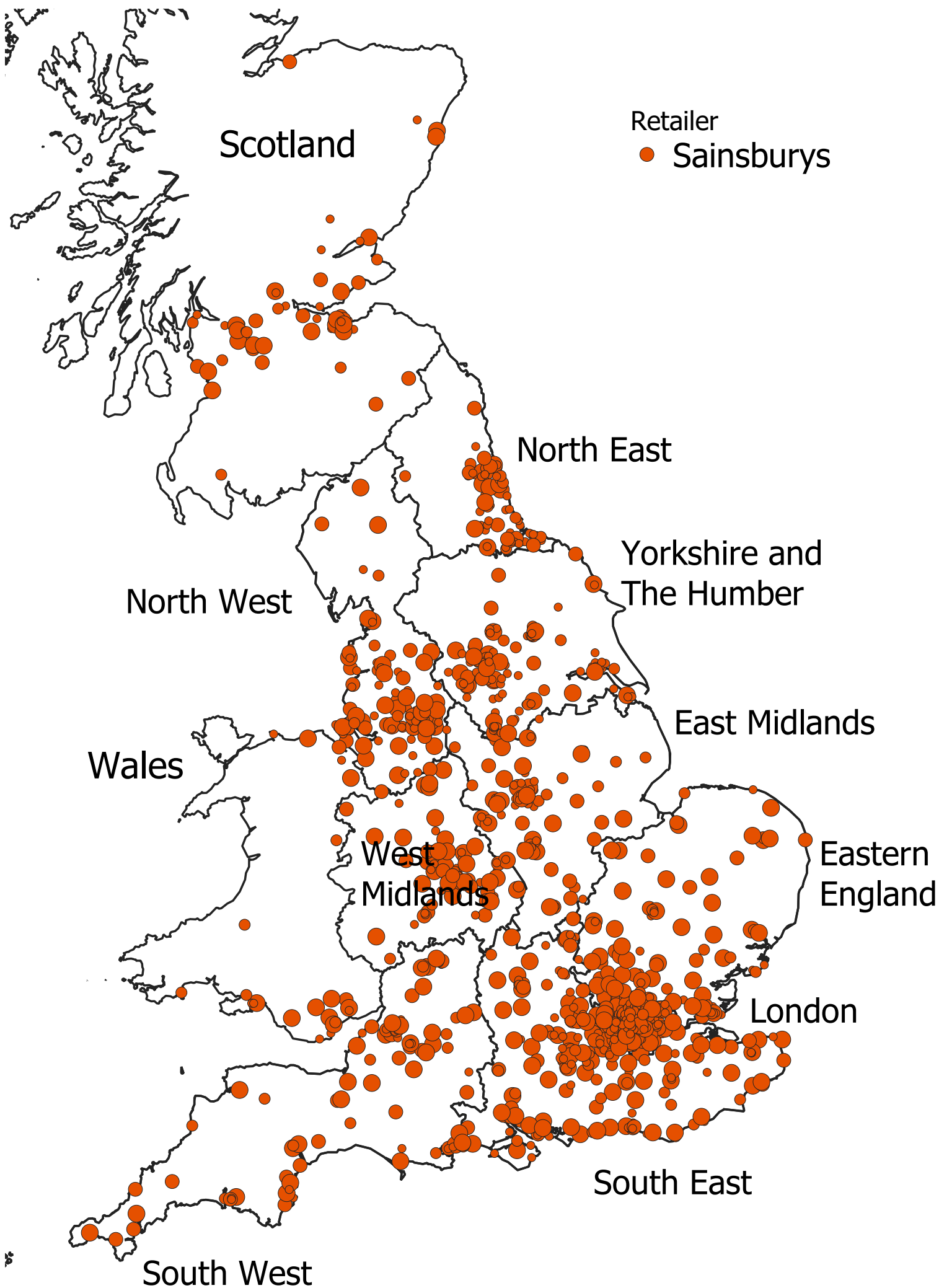

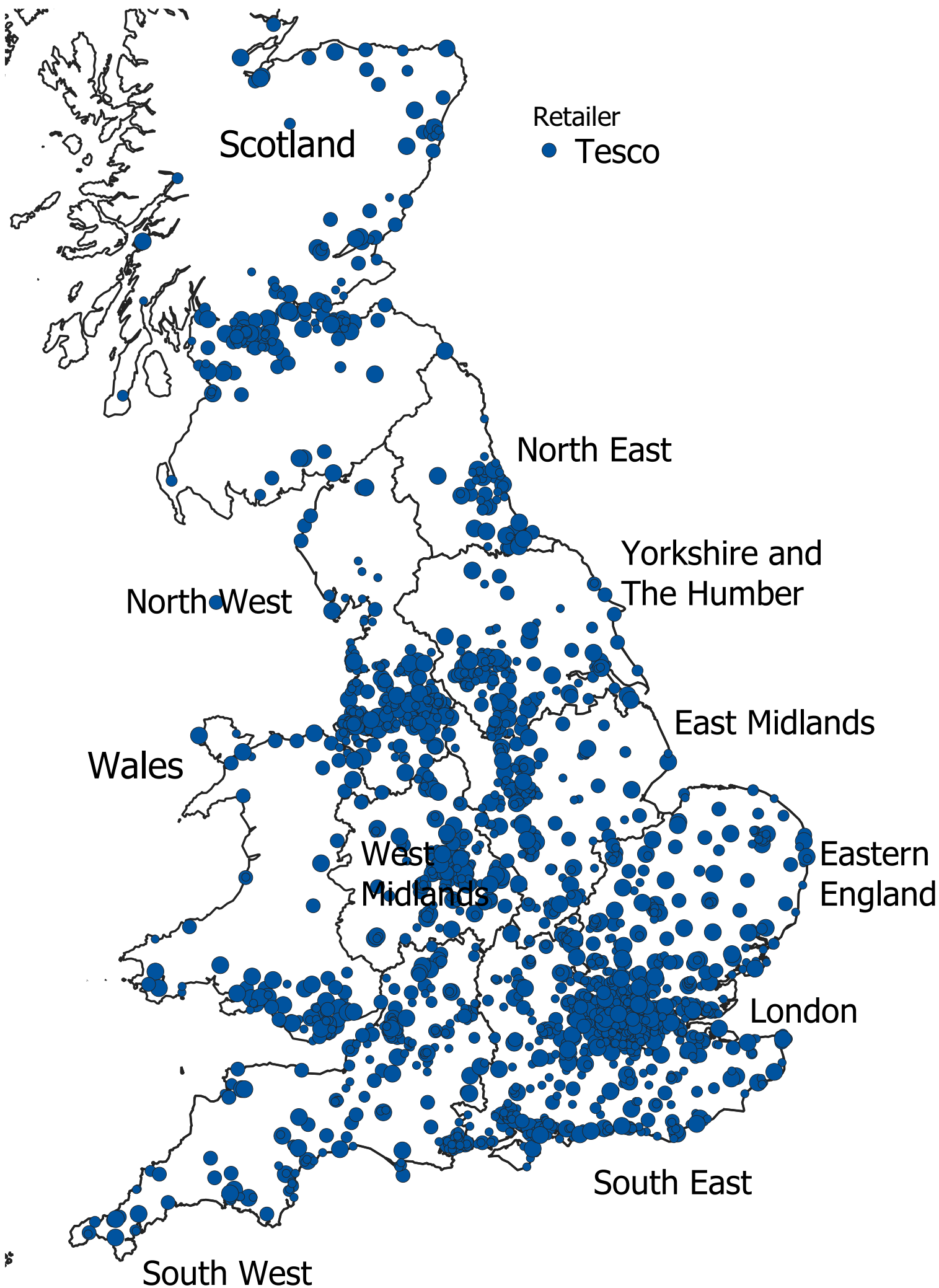

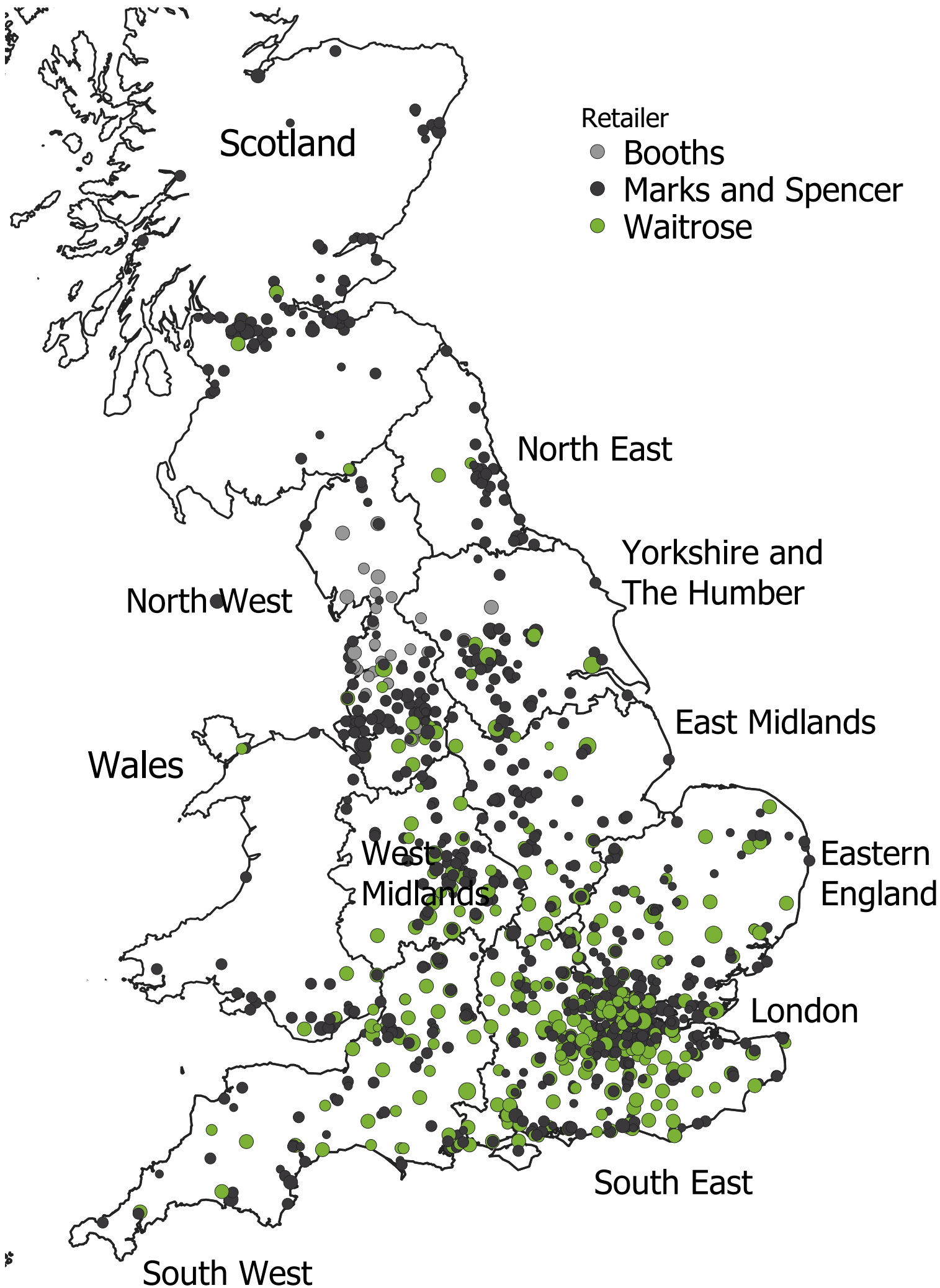

Supplement: Supplementary file 3 — Supplementary file3 (PDF 2462 KB) [file 10901_2021_9904_MOESM3_ESM.pdf]
